# Supplementary material for: Subthreshold micropulse laser versus standard laser for the treatment of central-involving diabetic macular oedema with central retinal thickness of <400µ: a cost-effectiveness analysis from the DIAMONDS trial
Source: BMJ Open. 2023 Oct 18;13(10):e067684. doi: 10.1136/bmjopen-2022-067684 (PMC10603454; doi:10.1136/bmjopen-2022-067684)
Supplement: Supplementary data [file bmjopen-2022-067684supp001.pdf]

Supplemental Tables

Supplemental Table A1: Unit costs

Resource Item	Unit Cost (£, 2020)	Measurement unit	Source
**Staff Costs**			
Consultant	114	per working hour	PSSRU 2020, page 158
Associate specialist/Staff Grade	117	per working hour	PSSRU 2020, page 158
Retina Fellow	50	per working hour	PSSRU 2019, page 158
Ophthalmic photographer/ Imaging technician	52	per working hour	PSSRU 2020, page 148
**Anti-vascular endothelial growth factor costs**			
Ranibizumab	569	Per dose	NHS Reference Costs 2019-20
Aflibercept	634	Per dose	NHS Reference Costs 2019-20
Bevacizumab	277	Per dose	NHS Reference Costs 2019-20

**Laser equipment costs**				
**Laser Type**	**Current Cost[a]**	**Lifespan (years)**	**Total annual discounted costs**	**Cost per patient[b]**
Complete scanning Laser Module TxCell/Haag Streit Fit/ IQ577nm with Micropulse®[c]	£79,800 - purchase price<br>£6,990 – total cost for a 5-year preventative maintenance contract (with the first 2 years being warranty) plus VAT	14	£8,860	£2.95
Nidek GYC-1000 Laser (including installation)[d]	£14,090 - purchase price<br>£3,653 - maintenance over 5 years	7	£3,113	£1.04
Pascal Laser[e]	£51,522 - purchase price (excluding optional extras)<br>£1,548 - maintenance cost	14	£6,266	£2.09
Argon laser (new design high quality ophthalmic laser)	£14,000 – purchase price<br>£809 - maintenance costs	7	£3,010	£1.03

[a] When required, costs were inflated to 2019/20 prices using the NHS Cost Inflation Index (NHSCII). [b] Annual throughput estimate was 3,000: (Personal communication, Noemi Lois, email dated 21 April 2021).[c] Equipment price quotation: Carleton Ltd (via email on 25 July 2016). [d] Equipment price quotation: Birmingham Optical (via email on 25 July 2016). [e] Equipment price quotation: Topcon Ireland Medical (via email on 23 April 2021)
NHS: National Health Service

Reproduced with permission from Lois et al (2022).(1) This is an open access article distributed in accordance with the Creative Commons Attribution 4.0 Unported (CC BY 4.0) license, which permits others to copy, redistribute, remix, transform and build upon this work for any purpose, provided the original work is properly cited, a link to the licence is given, and indication of whether changes were made. See: https://creativecommons.org/licenses/by/4.0/. The table includes minor additions and formatting changes to the original table.

Supplemental Table A2: VisQol scores at baseline, 12 months and 24 months post-randomisation by VisQol dimension and trial group

Timepoint	VisQol Dimension	Subthreshold Micropulse laser (N = 133)		Standard threshold laser (N = 132)		Between-Group Difference (95% CI)	
		n	Unadjusted mean (SD)	n	Unadjusted mean (SD)		p-value
Baseline	Injure	130	0.953 (0.14)	116	0.967 (0.093)	-0.023 (-0.007 to 0.053)	0.141
	Cope	130	0.942 (0.109)	116	0.956 (0.119)	0.001 (-0.025 to 0.028)	0.915
	Friendships	130	0.915 (0.147)	116	0.940 (0.143)	0.003 (-0.031 to 0.037)	0.879
	Assistance	131	0.970 (0.093)	116	0.983 (0.080)	-0.001 (-0.023 to 0.02)	0.904
	Roles	131	0.927 (0.181)	116	0.961 (0.117)	0.025 (-0.007 to 0.057)	0.119
	Confidence	131	0.936 (0.158)	116	0.970 (0.075)	0.015 (-0.012 to 0.041)	0.269
12 months	Injure	130	0.975 (0.082)	109	0.950 (0.177)	-0.018 (-0.05 to 0.014)	0.272
	Cope	130	0.943 (0.105)	109	0.957 (0.121)	-0.007 (-0.036 to 0.021)	0.614
	Friendships	130	0.918 (0.141)	109	0.944 (0.143)	-0.008 (-0.045 to 0.028)	0.650
	Assistance	129	0.969 (0.117)	110	0.979 (0.086)	-0.007 (-0.030 to 0.015)	0.528
	Roles	129	0.953 (0.121)	110	0.966 (0.083)	-0.005 (-0.039 to 0.029)	0.769
	Confidence	129	0.950 (0.122)	110	0.963 (0.076)	-0.005 (-0.033 to 0.023)	0.710
24 months	Injure	113	0.985 (0.047)	112	0.955 (0.159)	0.004 (-0.029 to 0.037)	0.804
	Cope	112	0.963 (0.099)	111	0.956 (0.159)	0 (-0.030 to 0.028)	0.963
	Friendships	130	0.918 (0.127)	111	0.939 (0.136)	-0.005 (-0.042 to 0.032)	0.805
	Assistance	112	0.991 (0.043)	113	0.977 (0.138)	-0.002 (-0.0248 to 0.021)	0.880
	Roles	113	0.966 (0.113)	113	0.953 (0.138)	-0.012 (-0.047 to 0.022)	0.478
	Confidence	113	0.975 (0.046)	112	0.954 (0.121)	-0.009 (-0.037 to 0.020)	0.546

Injure: Likely to injure self; Cope: Coping with life demands; Friendships: Ability to have friendships; Assistance: Organising assistance; Roles: Difficult to fulfil roles; Confidence: Confidence to join activities.
CI: confidence interval.

Supplemental Table A3: NEI-VFQ-25 Subscale and Composite Scores in participants treated with micropulse subthreshold laser vs. standard threshold laser

Trial group by timepoint	Variable	Observations	Mean	Standard deviation	Minimum	Maximum
**Subthreshold Micropulse laser**						
Baseline	*NEI-VFQ-25 Composite Score*	*131*	*86.38*	*13.83*	*30.88*	*100*
	General Health	131	46.95	25.39	0.00	100
	General Vision	130	72.15	13.75	40.00	100
	Ocular Pain	131	85.02	20.65	0.00	100
	Near Activities	130	80.00	19.84	8.33	100
	Distance Activities	130	87.82	16.09	16.67	100
	Vision Social Function	130	94.62	13.56	37.50	100
	Vision Mental Health	131	82.16	19.95	18.75	100
	Vision Role Difficulties	130	83.85	23.11	0.00	100
	Vision Dependency	131	92.68	19.28	8.33	100
	Driving	92	92.84	12.08	33.33	100
	Color Vision	129	96.71	11.85	50.00	100
	Peripheral Vision	130	88.85	18.95	25.00	100
12 months	*NEI-VFQ-25 Composite Score*	*113*	*89.61*	*9.99*	*45.92*	*100*
	General Health	113	50.00	23.62	0.00	100
	General Vision	112	74.11	13.05	40.00	100
	Ocular Pain	113	89.82	17.40	12.50	100
	Near Activities	113	83.67	19.30	8.33	100
	Distance Activities	112	90.29	15.06	33.33	100
	Vision Social Function	113	96.68	8.35	50.00	100
	Vision Mental Health	113	86.06	15.38	18.75	100
	Vision Role Difficulties	112	86.27	20.95	0.00	100
	Vision Dependency	113	96.31	12.35	25.00	100
	Driving	77	94.53	9.57	50.00	100
	Color Vision	113	98.45	8.37	25.00	100
	Peripheral Vision	112	93.08	14.32	50.00	100
24 months	*NEI-VFQ-25 Composite Score*	*114*	*87.19*	*14.08*	*22.65*	*100*
	General Health	114	52.85	29.33	0.00	100
	General Vision	114	72.63	15.57	20.00	100
	Ocular Pain	114	88.38	16.61	25.00	100
	Near Activities	114	81.18	21.55	0.00	100
	Distance Activities	114	87.35	17.57	12.50	100
	Vision Social Function	113	95.24	12.87	25.00	100
	Vision Mental Health	113	83.13	20.22	0.00	100
	Vision Role Difficulties	113	85.07	24.31	0.00	100
	Vision Dependency	111	93.09	19.72	0.00	100
	Driving	81	91.82	13.57	16.67	100
	Color Vision	110	97.73	9.90	50.00	100
	Peripheral Vision	113	88.50	20.60	25.00	100
**Standard threshold laser**						

Baseline	*NEI-VFQ-25 Composite Score*	*130*	*87.00*	*12.73*	*44.63*	*100*
	General Health	130	51.92	25.22	0.00	100
	General Vision	130	72.92	15.52	40.00	100
	Ocular Pain	130	85.38	17.82	25.00	100
	Near Activities	130	80.16	19.33	25.00	100
	Distance Activities	130	89.01	14.17	41.67	100
	Vision Social Function	130	93.94	13.68	25.00	100
	Vision Mental Health	130	80.87	20.62	6.25	100
	Vision Role Difficulties	129	82.75	26.48	0.00	100
	Vision Dependency	130	93.72	14.57	33.33	100
	Driving	91	95.05	9.38	50.00	100
	Color Vision	130	96.35	11.69	25.00	100
	Peripheral Vision	130	91.54	16.63	25.00	100
12 months	*NEI-VFQ-25 Composite Score*	*117*	*88.47*	*13.78*	*29.21*	*100*
	General Health	116	49.78	25.00	0.00	100
	General Vision	116	75.17	14.59	40.00	100
	Ocular Pain	117	88.25	15.25	37.50	100
	Near Activities	117	83.62	19.29	8.33	100
	Distance Activities	117	88.89	17.02	25.00	100
	Vision Social Function	116	94.29	14.22	25.00	100
	Vision Mental Health	117	86.38	18.99	6.25	100
	Vision Role Difficulties	117	85.26	24.27	0.00	100
	Vision Dependency	116	92.74	19.37	0.00	100
	Driving	81	93.26	14.65	33.33	100
	Color Vision	115	98.48	6.86	50.00	100
	Peripheral Vision	115	91.96	16.07	25.00	100
24 months	*NEI-VFQ-25 Composite Score*	*115*	*88.80*	*13.78*	*29.08*	*100*
	General Health	115	51.96	24.59	0.00	100
	General Vision	114	74.39	14.94	20.00	100
	Ocular Pain	115	89.35	15.55	37.50	100
	Near Activities	115	82.79	20.03	16.67	100
	Distance Activities	115	89.53	17.41	16.67	100
	Vision Social Function	115	94.57	13.05	12.50	100
	Vision Mental Health	115	85.43	21.26	0.00	100
	Vision Role Difficulties	114	86.73	21.76	0.00	100
	Vision Dependency	114	94.01	19.03	0.00	100
	Driving	78	96.42	7.35	58.33	100
	Color Vision	115	96.52	11.89	25.00	100
	Peripheral Vision	115	91.96	16.74	25.00	00

Reproduced with permission from Lois et al (2022).(1) This is an open access article distributed in accordance with the Creative Commons Attribution 4.0 Unported (CC BY 4.0) license, which permits others to copy, redistribute, remix, transform and build upon this work for any purpose, provided the original work is properly cited, a link to the licence is given, and indication of whether changes were made. See: https://creativecommons.org/licenses/by/4.0/. The table includes minor additions and formatting changes to the original table.

Supplemental Table A4: Frequency of laser treatments and use of anti-VEGFs or steroids as rescue treatment over the 24-month follow-up period

	Subthreshold Micropulse Laser	Standard Threshold Laser	Difference (95% CI)	P-value
**Number of laser treatments used from baseline to month 24 in study eye [a b]**	2.37 (0.11) n=133	1.89 (0.11) n=132	0.48 (0.18, 0.79)	0.002
**Number of patients with at least one steroid injection in study eye (as additional treatment) from baseline to month 24[c]**	0 (0.0%)	1 (0.8%)		
**Number of patients receiving at least one anti-VEGF treatment (as additional treatment) from baseline to month 24[c]**	24 (18.1%) n=133	28 (21.2%) n=132	OR: 0.78 (0.42 – 1.45)	0.44
			% point difference: -2.8 (-13.1 – 7.5)	0.59
**Number of anti-VEGF treatments (as additional treatment) from baseline to month 24[a d]**	0.80 (0.23) n=133	1.30 (0.23) n=132	-0.50 (-1.14 – 0.14)	0.13
**Number of anti-VEGF treatments (as additional treatment) from baseline to month 24 [d e]**				
**1-2**	4 (16.7%) n=24	7 (25.0%) n=28		
**3-4**	10 (41.7%) n=24	7 (25.0%) n=28		
**5-10**	10 (41.7%) n=24	9 (32.1%) n=28		
**>10**	0 (0.0%) n=24	5 (17.9%) n=28		

[a] Mean (SE) presented for continuous outcomes [b] Number of laser treatments, were analysed using linear regression with adjustment for baseline BCVA and minimisation variables. [c] number of patients receiving at least one additional treatment (defined as at least one anti-VEGF or steroid), were analysed using logistic regression models with adjustment for the minimisation variables. [d] Number of steroid injections and number of anti-VEGF treatments (as mean and number (%) in categories), were analysed using linear regression with adjustment for minimisation variables. [e]n (%) based on no. of patients receiving anti VEGF treatments.

anti-VEGF: anti-vascular endothelial growth factor; CI: confidence interval; OR: odds ratio; RR: risk ratio

References

1. Lois N, Campbell C, Waugh N, Azuara-Blanco A, Maredza M, Mistry H, et al. DIAbetic Macular Oedema aNd Diode Subthreshold micropulse laser (DIAMONDS): A pragmatic, multicentre, allocation concealed, double-masked prospective, randomised, non-inferiority, clinical trial. Health Technology Assessment. 2022;26(50):1-86.
