# Supplementary material for: Subthreshold micropulse laser versus standard laser for the treatment of central-involving diabetic macular oedema with central retinal thickness of <400µ: a cost-effectiveness analysis from the DIAMONDS trial
Source: BMJ Open. 2023 Oct 18;13(10):e067684. doi: 10.1136/bmjopen-2022-067684 (PMC10603454; doi:10.1136/bmjopen-2022-067684)
Supplement: Author's manuscript [file bmjopen-2022-067684.draft_revisions.pdf]

BMJ Open is committed to open peer review. As part of this commitment we make the peer review history of every article we publish publicly available.

When an article is published we post the peer reviewers' comments and the authors' responses online. We also post the versions of the paper that were used during peer review. These are the versions that the peer review comments apply to.

The versions of the paper that follow are the versions that were submitted during the peer review process. They are not the versions of record or the final published versions. They should not be cited or distributed as the published version of this manuscript.

BMJ Open is an open access journal and the full, final, typeset and author-corrected version of record of the manuscript is available on our site with no access controls, subscription charges or pay-per-view fees (http://bmjopen.bmj.com).

If you have any questions on BMJ Open's open peer review process please email info.bmjopen@bmj.com

BMJ Open

Subthreshold micropulse laser versus standard laser for the treatment of central-involving diabetic macular oedema with central retinal thickness of <400μ: a cost-effectiveness analysis from the DIAMONDS trial

Journal:	*BMJ Open*
Manuscript ID	bmjopen-2022-067684
Article Type:	Original research
Date Submitted by the Author:	24-Aug-2022
Complete List of Authors:	Mistry, Hema; University of Warwick; University Hospitals Coventry and Warwickshire NHS Trust<br>Maredza, Mandy ; University of Warwick<br>Campbell, Christina; Northern Ireland Clinical Trials Unit<br>Lois, Noemi; Wellcome-Wolfson Institute for Experimental Medicine, Ophthalmology
Keywords:	OPHTHALMOLOGY, Diabetic retinopathy < DIABETES & ENDOCRINOLOGY, HEALTH ECONOMICS

SCHOLARONE™
Manuscripts

I, the Submitting Author has the right to grant and does grant on behalf of all authors of the Work (as defined in the below author licence), an exclusive licence and/or a non-exclusive licence for contributions from authors who are: i) UK Crown employees; ii) where BMJ has agreed a CC-BY licence shall apply, and/or iii) in accordance with the terms applicable for US Federal Government officers or employees acting as part of their official duties; on a worldwide, perpetual, irrevocable, royalty-free basis to BMJ Publishing Group Ltd ("BMJ") its licensees and where the relevant Journal is co-owned by BMJ to the co-owners of the Journal, to publish the Work in this journal and any other BMJ products and to exploit all rights, as set out in our licence.

The Submitting Author accepts and understands that any supply made under these terms is made by BMJ to the Submitting Author unless you are acting as an employee on behalf of your employer or a postgraduate student of an affiliated institution which is paying any applicable article publishing charge ("APC") for Open Access articles. Where the Submitting Author wishes to make the Work available on an Open Access basis (and intends to pay the relevant APC), the terms of reuse of such Open Access shall be governed by a Creative Commons licence – details of these licences and which Creative Commons licence will apply to this Work are set out in our licence referred to above.

Other than as permitted in any relevant BMJ Author's Self Archiving Policies, I confirm this Work has not been accepted for publication elsewhere, is not being considered for publication elsewhere and does not duplicate material already published. I confirm all authors consent to publication of this Work and authorise the granting of this licence.

For peer review only

Subthreshold micropulse laser versus standard laser for the treatment of central-involving diabetic macular oedema with central retinal thickness of <400μ: a cost-effectiveness analysis from the DIAMONDS trial

Hema Mistry [1,5*], Mandy Maredza[2], Christina Campbell [3], Noemi Lois[4] on behalf of the DIAMONDS study group.

Affiliations

[1] Warwick Clinical Trials Unit, Warwick Medical School, University of Warwick, Coventry, CV4 7AL, UK.

[2] Division of Health Sciences, Warwick Medical School, University of Warwick, Coventry, CV4 7AL, UK.

[3] Northern Ireland Clinical Trials Unit, 7 Lennoxvale, Belfast, BT9 5BY, UK.

[4] Wellcome-Wolfson Institute for Experimental Medicine, Queen's University Belfast, 97 Lisburn Road, Belfast, BT9 7BL, UK.

[5]University Hospitals Coventry and Warwickshire NHS Trust, Clifford Bridge Road, Coventry, CV2 2DX, UK

Correspondence to: Dr Hema Mistry, Warwick Clinical Trials Unit, Warwick Medical School, University of Warwick, Coventry, UK; Email: Hema.Mistry@warwick.ac.uk; Tel: +44 (0)2476 151183

Key words:

Diabetic macular oedema, DMO, diabetic macular edema, DME, macular laser, micropulse, subthreshold, threshold, costs, quality of life, EQ-5D, EuroQol, QALY, cost-effectiveness

For peer review only

Abstract (currently 297 words, 300 words max)

Objectives

To estimate the economic costs, health-related quality of life outcomes and cost-effectiveness of subthreshold micropulse laser (SML) versus standard laser (SL) for the treatment of diabetic macular oedema (DMO) with central retinal thickness (CRT) of <400µ.

Design

An economic evaluation was conducted within a pragmatic, multicentre, randomised clinical trial, DIAMONDS.

Setting

18 UK Hospital Eye Services.

Participants

Adults with diabetes and centre involving DMO with CRT <400µ.

Interventions

Participants (n=266) were randomised 1:1 to receive SML or SL.

Methods

The base-case used an intention-to-treat approach conducted from a UK National Health Service (NHS) and personal social services (PSS) perspective. Costs (2019–2020 prices) were collected prospectively over the 2-year follow-up period. A bivariate regression of costs and quality-adjusted life-years (QALYs), with multiple imputation of missing data, was conducted to estimate the incremental cost per QALY gained and the incremental net monetary benefit of SML in comparison to SL. Sensitivity analyses explored uncertainty and heterogeneity in cost-effectiveness estimates.

Results

One participant in the SL arm withdrew consent for data to be used; data from the remaining 265 participants were included in analyses. Mean (standard error [SE]) NHS and PSS costs over 24 months were £735.09 [£111.85] in the SML arm versus £1099.70 [£195.40] in the SL arm (p=0.107). Mean (SE) QALY estimates were 1.493 [0.024] versus 1.485 [0.020], respectively (p=0.780), giving an

For peer review only

insignificant difference of 0.008 QALYs. The probability SML is cost-effective at a threshold of £20,000 per QALY was 76%.

Conclusions

There were no statistically significant differences in EQ-5D-5L scores or costs between SML and SL. Given these findings and the fact that SML does not burn the retina, unlike SL, and has equivalent efficacy to SL, it may be preferred for the treatment of people with DMO with CRT <400µ.

Trial registration number

ISRCTN17742985; NCT03690050

For peer review only

Strengths and limitations of this study

- This study is based on data obtained in a high-quality, methodologically robust and appropriately powered pragmatic randomised clinical trial called DIAMONDS.
- DIAMONDS showed that, for people with diabetic macular oedema (DMO) and central retinal thickness (CRT)<400μ, subthreshold micropulse laser (SML) is equivalent in efficacy to standard laser (SL).
- This is the first study to compare the cost-effectiveness of SML with that of SL for the treatment of DMO.
- There was a low rate of missing data for both costs and outcomes.
- One limitation of this study is that we used published costs of anti-VEGF drugs, rather than costs to the NHS which would incorporate confidential price discounts for these drugs.

For peer review only

Manuscript word count 2686 words (including the 3 tables); max 4000 words; up to 5 images/tables

Introduction

Diabetic macular oedema (DMO) is a visual threatening complication of diabetes, occurring in approximately 7% of people living with diabetes.(1) Given the high and continuously increasing prevalence of diabetes,(2) DMO is a frequent eye disease requiring treatment in ophthalmic clinics in the UK and worldwide. In DMO fluid accumulates in the center of the retina, the macula, which is the area of the retina responsible for providing central vision.(3) The purpose of the treatment is to restore the anatomy of the macula by clearing up this fluid and restoring vision. Treatments include intraocular injections of anti-vascular endothelial growth factor (anti-VEGF) drugs or steroids and macular laser. The National Institute for Health and Care Excellence (NICE) recommends anti-VEGF therapy for people with more severe forms of DMO, with central retinal (macular) thickness (CRT) of 400µ or above, as measured in scans obtained using an imaging modality called optical coherence tomography (OCT).(4-6) For milder forms of DMO (CRT <400µ) NICE recommends macular laser. Intravitreal steroids are advised for people that do not respond to the above-mentioned therapies.

DIAbetic Macular Oedema aNd Diode Subthreshold micropulse laser (DIAMONDS) was a pragmatic, allocation-concealed, double-masked, multicentre, randomised, non-inferiority clinical trial which compared the clinical effectiveness and cost-effectiveness of subthreshold micropulse laser (SML) and standard laser (SL) for the treatment of people with DMO with CRT of <400µ. DIAMONDS participants were randomised 1:1 to receive SML (577 nm) or SL (e.g. using argon, frequency-doubled neodymium-doped yttrium aluminium garnet [Nd:YAG] 532 nm laser). Laser treatment could be repeated as needed, using the allocated laser at randomisation and rescue treatment with anti-VEGFs or steroids was allowed. The primary outcome was the mean change in best-corrected visual acuity (BCVA) in the study eye at 24 months. DIAMONDS found SML and SL to have equivalent

For peer review only

clinical efficacy.(7, 8) Herein, we present the detailed within-trial economic evaluation comparing costs and benefits of the two laser modalities, SML and SL.

Methods

As detailed in the Health Economics Analysis Plan (HEAP)(9) we planned to conduct a within-trial analysis comparing the cost-effectiveness of SML with SL. The protocol for the DIAMONDS trial envisaged that economic modelling might be required if visual outcomes differed between arms.(10) The DIAMONDS protocol was designed to minimise visual loss in participants and, thus, repeating laser treatment or undertaking rescue treatment with intravitreal anti-VEGF drugs and/or steroids if criteria for rescue were met, were allowed in either arm of the trial. DIAMONDS recruited 266 participants, 116 (87%) in the SML arm and 115 (86%) in the SL arm had primary outcome data, fulfilling the requirements of the power calculation (113 participants with BCVA data at month 24 were required). DIAMONDS found SML and SL to be equivalent in terms of the primary outcome.(7, 8) Hence, economic modelling was not required. Herein, we report detailed economic costs, health-related quality of life (HRQoL) outcomes and cost-effectiveness of SML versus SL.

The economic evaluation took the form of a cost-utility analysis, expressed in terms of cost per quality-adjusted life-year (QALY) gained. The study was conducted over the 24-month time horizon of the trial. The primary analysis adopted a National Health Service (NHS) and personal and social service (PSS) perspective. Costs and outcomes in the second year of follow-up were discounted at 3.5% in line with the NICE reference case.(11)

Resource use data were collected and reported on trial case record forms (CRFs) at scheduled clinic visits at 4, 8, 12, 16, 20 and 24 months. Data were collected on the costs of laser treatment, both at the initial laser session and at subsequent ones if required, outpatient visits, and intravitreal anti-VEGF and/or steroid treatment (costs of drugs and administration) if rescue was required. All costs

For peer review only

were expressed in Great British pounds sterling and valued in 2019–20 prices. If costs were not in line, they were inflated to 2019-20 prices using the NHS Cost Inflation Index (NHSCII).(12)

The costs of laser treatment included staff and equipment costs (capital and maintenance costs of laser machines). Unit costs for staff were obtained from the Unit Costs of Health and Social Care 2019 compendium (12) and were based on the time it took to for each procedure to be undertaken. These times were recorded on CRFs and included: (i) the time taken to obtain fundus fluorescein angiography (FFA) and spectral domain optical coherence tomography (SD-OCTs) scans to guide laser treatment (if used) and (ii) time taken by ophthalmologists to perform the laser procedure, including the time for counselling the participant. Costs of laser machines were obtained from manufacturers. An annual equivalent cost of equipment was obtained by annuitising the capital costs of the item over its useful life span and applying a discount rate of 3.5% per annum. A per-patient cost of equipment was estimated by assuming that the laser machines were used to treat 3,000 patients per year.

Data were also collected on any outpatient attendances or hospital admissions related to DMO or the treatments. Where an outpatient attendance was reported but no procedure undertaken, the average unit cost of an outpatient ophthalmology visit was used (varying between £80-£101 per consultation depending on whether the consultation was 'non-consultant' versus 'consultant-led').(13) The CRFs recorded data on the grade of professional that attended the patient, for example, if a consultant attended to the patient, then the consultant-led unit cost was applied. Where a procedure(s) was undertaken as part of the visit the relevant HRG code was derived using the HRG4+ Reference Costs Grouper Software (NHS Digital, Leeds, UK).

CRFs also recorded information on other tests or investigations, medication use including anti-VEGF/steroids or other rescue treatments. Anti-VEGF and steroid drugs were separately costed as

For peer review only

these are considered an unbundled HRG. Costing of laser retreatments followed the same approach as costing for the index (first session) laser procedure.

Unit costs were derived from national compendia in accordance with NICE's Guide to the Methods of Technology Appraisal.(11) The key databases included the Department of Health and Social Care's Reference Costs 2018–19 schedules,(13) the PSSRU's Unit Costs of Health and Social Care 2020 compendium (12), 2020 volume of the British National Formulary.(14) Supplemental Table A1 gives a summary of the unit costs for resource use and the laser equipment. Resource inputs were valued by attaching unit costs.

The HRQoL of trial participants was assessed at baseline and at 12- and 24-months post-randomisation using the EQ-5D-5L instrument. The EQ-5D-5L questionnaire was used to generate QALYs. (15) The QALY is a measure that combines quantity and quality of life lived into a single metric, with one QALY equating to one year of full health. HRQoL was also assessed using two vision specific measures: the National Eye Institute Visual Functioning Questionnaire - 25 (NEI-VFQ-25) and Vision and Quality of Life Index (VisQoL).(16-18) The NEI-VFQ-25 is a validated questionnaire that has been used widely to evaluate visual outcomes in patients with eye diseases including diabetic retinopathy and DMO. The VisQol questionnaire has not been widely validated but is shorter than the NEI-VFQ-25 with only six attributes: physical well-being, independence, social well-being, self-actualisation, planning and organisation. The utilities for VisQoL were developed using a time-trade off exercise in people who were visually impaired which included patients with age-related macular degeneration, diabetic retinopathy and glaucoma.(18)

Summary statistics were generated for resource use variables, health utility values and QALYs by treatment allocation and assessment time point. For resource use and costs, mean values were compared between groups using two sample t-tests. Differences between groups, along with 95%

For peer review only

confidence intervals (CIs) were estimated using non-parametric bootstrap estimates (10,000 replications). For HRQoL, these were presented as mean values with their associated standard errors. Between-group differences were compared using the two-sample t-test.

Multiple imputation by chained equations was used to predict missing health status (utility) scores and costs based on the assumption that data were missing at random (MAR). Twenty imputed data sets were generated and used to inform the base-case analyses. Parameter estimates were pooled across the 20 imputed data sets using Rubin's rules to account for between- and within-imputation components of variance terms associated with parameter estimates.(19)

The base-case cost-effectiveness analysis was performed using an intention-to-treat approach. Mean incremental costs and QALYs were estimated using seemingly unrelated regression (SUR) methods that account for the correlation between costs and outcomes. The SUR adjusted for covariates (baseline utilities, baseline body mass index (BMI), baseline best corrected distance visual acuity (BCVA), patient-reported previous use of anti-VEGF at baseline and previous use of macular laser).

Results

As reported elsewhere (7, 8) there was no difference in the clinical effectiveness of SML and SL. Table 1 shows that there were also no statistically significant differences between laser groups in EQ-5D-5L scores at baseline, 12 and 24 months.

For peer review only

Table 1: EQ-5D-5L utility scores at baseline, 12 and 24 months and QALYs (base-case, imputed analysis)

Variable	Subthreshold micropulse laser (n = 133)	Standard laser (n = 132)	Between-Group Difference (95% CI)	p-value
	Mean (SE)	Mean (SE)		
**EQ-5D Utility Scores***				
Baseline	0.758 (0.267)	0.772 (0.226)	-0.014 (-0.074 to 0.046)	0.640
12 months	0.767 (0.250)	0.758 (0.017)	0.009 (-0.041to 0.059)	0.717
24 months	0.739 (0.278)	0.743 (0.279)	-0.004 (-0.064 to 0.056)	0.897
**EQ-5D-5L QALYs**				
Over 2 years	1.493 (0.024)	1.485 (0.024)	0.008 (-0.061 to 0.075)	0.836

*Analysis adjusted for participant age, gender, baseline BCVA (Best Corrected Distance Visual Acuity) and participant's previous use of Anti-VEGF (Anti-Vascular Endothelial Growth Factor) and laser therapy at baseline, with repeated measures within participant and site.
SE = standard error; CI = confidence interval

Furthermore, there were no statistically significant differences between the two laser treatment groups for any of the VisQoL dimensions or NEI-VFQ-25 subscales at any follow-up time point (Supplemental Table A2 and Supplemental Table A3, respectively).

The mean numbers of laser treatments performed were 2.4 in the SML and 1.9 in the SL arm. This difference was statistically significant (p=0.002) (Supplemental Table A4), but equated to less than one further session of laser in the SML arm when compared with the SL arm during the 2-year trial. Furthermore, this difference was driven by a small number of participants requiring a higher number of lasers sessions in the SML group (13 participants required 6 or 7 laser treatments in the SML arm compared to 2 in the SL arm). Eighteen percent of participants in the SML arm and 21% in the SL arm required rescue treatments in the study eye (almost all with anti-VEGF drugs; in addition, only one participant had a steroid injection). The average numbers of anti-VEGF treatments per arm from

For peer review only

baseline to 24 months were 1.06 in the SML arm and 1.96 in the SL (Supplemental Table A4). The number in the SL arm was skewed by five participants who received 10 or more injections.

Table 2 summarises the NHS costs associated with resource use in the base-case (imputed) analysis by cost category and follow-up period. Costs of the first laser procedure are reported separately from those of subsequent laser retreatments. The total costs of laser therapy for each participant includes costs of the first laser procedure plus any subsequent laser retreatments they had. The mean (standard error (SE)) cost for the first laser procedure (including costs of performing OCT and FFA to guide laser treatment, if done) was £45.59 (1.64) for the SML compared with £42.29 (1.69) for the SL; the difference was not statistically significant(p=0.09). The mean total NHS and PSS costs were lower in SML compared with the SL (£735.09 vs. £1099.70) between baseline to 24 months post-randomisation; this difference was not statistically significant at the 5% level. The CI around total costs were wide and overlapped and the difference was driven by the higher number of anti-VEGF rescue injections.

Table 2: *Economic costs by trial allocation arm and cost component category for the entire follow-up period in base-case (imputed) analysis (£, 2019-20 prices)*

**Parameter**	**Subthreshold Micropulse laser**	**Standard threshold laser**	**Mean Difference**	**Bootstrap 95% CI**
	**Mean Costs (SE)**	**Mean Costs (SE)**		
Index Laser Procedure	45.59 (1.64)	42.29 (1.69)	3.31	(-1.33 to 7.95)
Laser retreatments	53.02 (5.17)	41.69 (4.42)	11.32	(-2.08 to 24.73)
Outpatient care	124.85 (22.56)	130.32 (31.58)	-5.47	(-81.92 to 70.97)
Anti-VEGF drug costs	511.63 (105.85)	885.40 (183.29)	-373.77	(-791.01 to 43.48)
**Total NHS and PSS Costs**	**735.09 (111.85)**	**1,099.70 (195.40)**	**-364.61**	**(-807.09 to 77.87)**
SE = standard error; CI = confidence interval				

Over the 2-year follow-up period, participants in the SML arm, compared to the SL arm, experienced a non-statistically significant increase in QALYs of 0.008 (circa 3 days of good quality of life). In addition, the mean NHS and PSS costs were lower in the SML arm compared to the SL arm (mean cost difference –£365) – see Table 3. The confidence interval for the cost difference was wide and ranged from cost saving to cost increasing. Although neither costs nor benefits were statistically significantly different between SML and SL, the incremental cost-effectiveness ratio (ICER) for the base-case imputed analysis indicated that SML dominates, as average costs for this intervention were slightly lower and average benefits marginally higher (but not significantly so) than those for SL.

Table 3: Cost-effectiveness, cost/QALY (£, 2020): SML compared to SL

Mean incremental cost (95% CI)	Mean incremental QALY (95% CI)	ICER	Probability of cost-effectiveness*	Net Monetary Benefit (NMB)*
Base-case analysis - ITT approach: Imputed attributable costs and QALYs, covariate adjusted[a]				
-365 (-822 to 93)	0.008 (-0.059 to 0.075)	Dominant	0.763	520 (-925 to 1,965)

***Cost-effectiveness threshold is at £20,000/QALY threshold**

[a] Adjusted for baseline EQ-5D utility, BMI and minimisation variables at baseline (best corrected distance visual acuity and previous use of laser treatment)

CI = confidence interval; ICER = incremental cost-effectiveness ratio; ITT = intention-to-treat approach; QALY = quality-adjusted life year

Discussion

DIAMONDS was a pragmatic clinical trial carried out in 16 NHS ophthalmology departments throughout the UK. Participants were treated and followed as per routine clinical care. Cost data were collected prospectively. This analysis found that costs were slightly higher (but not statistically significantly so) in the SL arm compared to the SML arm, due to more participants in the SL arm needing higher numbers (10 or more) of anti-VEGF rescue injections. Given the wide confidence

For peer review only

intervals around the mean costs and the fact that total costs were not statistically significantly different, costs between SML and SL treatment arms seem comparable.

As noted in the main clinical effectiveness manuscript (7) there may be advantages of SML over SL, including the fact that SML does not cause any structural changes in the retina and, thus, can be repeated potentially indefinitely, as needed. This makes it also potentially safer in less experienced hands. Furthermore, for the same reason, it may allow training of non-specialist/non-medical staff who, currently, are already undertaking anti-VEGF injections in the UK and other parts of the world. This would help alleviating NHS capacity constraints. The clinical effectiveness results reported previously (7) and the HRQoL results presented here showed that SML is as clinically effective as SL at a similar cost.

In DIAMONDS, two vision-specific patient reported outcome quality of life instruments were used in addition to the generic preference-based EQ-5D-5L. Neither showed any statistically significant differences between laser arms. It has sometimes been mentioned that EQ-5D may not be as sensitive to detect changes in quality of life in visual disorders as the NEI-VFQ-25.(4) A mapping exercise from DIAMONDS data is underway to explore this important aspect.

One limitation in the current analysis is the fact that we had to use published costs of anti-VEGF drugs, rather than costs to the NHS. We know that there are confidential price discounts for these drugs when used in the NHS and this will most likely reduce the cost differential between the two arms. Another limitation is that DIAMONDS included only patients with CRT <400µ, so extrapolations with regard to the cost-effectiveness of SML when compared with SL in thicker retinas, where standard laser is known to be less effective, cannot be made.

For peer review only

Both SML and SL were successful in 80% of participants.(7) Macular laser treatment is much less expensive than anti-VEGF drugs and may be more convenient and acceptable to participants. Thus, SML should be considered as first line therapy for patients with central involving DMO with CRT of <400µ.

For peer review only

Contributor statement/Acknowledgements

The Authors would like to thank all of the DIAMONDS participants and the DIAMONDS patient and public involvement (PPI) group. We also thank Dr Pamela Royle for literature searches. We would also like to thank Iridex for providing a free loan for the laser equipment for the trial and William Moore, Joan Stauffer, George Marcellino (*in memoriam*), Gareth Hymas, Nick Fitrzyk, and Suzanne Kelly, for their support. Iridex had no role in the design, data analysis or interpretation of the DIAMONDS data or in the writing of this report. We thank also the NIHR HTA programme for providing funding for the trial and note that the views expressed in this paper are those of the authors and not necessarily those of the NHS, the NIHR or the Department of Health and Social Care.

Author contributions

Hema Mistry contributed to the design of the health economics plan and provided oversight of all aspects of the economic evaluation including its design, conduct, analysis and reporting of this paper.

Mandy Maredza (University of Warwick) conducted the analysis and reporting of the economic evaluation and contributed to the drafting of this paper.

Christina Campbell (Trial Statistician, NICTU) performed the statistical analyses, assisted with interpretation of data and contributed to the drafting of this paper.

Professor Noemi Lois (Clinical Professor of Ophthalmology, Wellcome-Wolfson Institute for Experimental Medicine, Queen's University Belfast) is the Chief Investigator for the DIAMONDS trial conceived the trial, with input from the DIAMONDS Study Group, and led it to its successful completion. In addition, Prof Lois contributed with the identification, recruitment, examination and treatment of all participants enrolled at the Belfast site, as well as to data collection, analysis and interpretation of the trial findings. She contributed to the drafting of this paper.

For peer review only

DIAMONDS Study Group

Noemi Lois, Queen's University and Royal Victoria Hospital, Belfast H&SC Trust; Augusto Azuara-Blanco, Queen's University and Royal Victoria Hospital, Belfast H&SC Trust; Christina Campbell, NICTU, Belfast; Danny McAuley, Queen's University and Royal Victoria Hospital, Belfast H&SC Trust; Mandy Maredza, Warwick University; Hema Mistry, Warwick University; Norman Waugh, Warwick University; Nachiketa Acharya, Sheffield Teaching Hospitals NHS Foundation Trust; Tariq M Aslam, Manchester Royal Eye Hospital, Central Manchester University Hospitals NHS Foundation Trust; Clare Bailey, Bristol Eye Hospital, University Hospitals Bristol NHS Foundation Trust; Victor Chong, Royal Free Hospital NHS Foundation Trust, London; Louise Downey, Hull and East Yorkshire Hospital, Hull and East Yorkshire NHS Trust; Haralabos Eleftheriadis, Kings College Hospital NHS Foundation Trust; Samia Fatum, John Radcliffe Hospital, Oxford University Hospitals NHS Foundation Trust; Sheena George, Hillingdon Hospitals NHS Foundation Trust; Faruque Ghanchi, Bradford Teaching Hospitals NHS Trust; Markus Groppe, Stoke Mandeville Hospital, Buckinghamshire NHS Trust; Robin Hamilton, Moorfields Eye Hospital NHS Foundation Trust; Geeta Menon, Frimley Park Hospital NHS Foundation Trust; Ahmed Saad, James Cook University Hospital, South Tees Hospitals NHS Foundation Trust; Sobha Sivaprasad, Moorfields Eye Hospital NHS Foundation Trust; Marianne Shiew, Hinchingbrooke Hospital North West Anglia NHS Trust; David H Steel, Sunderland Eye Infirmary, City Hospitals Sunderland NHS Foundation Trust; James Stephen Talks, Newcastle Eye Centre, Royal Victoria Infirmary, Newcastle upon Tyne Hospitals NHS Foundation Trust; Catherine Adams, NICTU, Belfast; Paul Doherty, NICTU, Belfast; Evie Gardener, NICTU, Belfast; Aby Joseph, NICTU, Belfast; Cliona McDowell, NICTU, Belfast; Matthew Mills, NICTU, Belfast; Mike Clarke, Queens University, Belfast.

Competing interests: HM: none; **MM**: none; **CC**: none; **NL**: none.

For peer review only

Funding: DIAMONDS was funded by the Health Technology Assessment (HTA) programme of the National Institute for Health Research (NIHR), United Kingdom NIHR-HTA [13/142/04]. The NIHR had no role in the design or conduct of this research. Iridex provided a free loan for the laser equipment for the trial. Iridex had no role in the design or conduct of the trial or on data analysis or interpretation of the DIAMONDS data or writing of this report.

Data sharing statement

All data requests should be submitted to the corresponding author for consideration.

Ethics statements

Ethics Approval

The protocol for the DIAMONDS trial was approved by ORECNI (15/NI/0197). A Clinical Trial Authorisation (CTA) was obtained from the Medicines and Healthcare products Regulatory Agency (MHRA) (32485/0029/001-0001). The trial was registered with the European Union Drug Regulating Authorities Clinical Trials (EudraCT) database (2015-001940-12) and in the International Standard Randomised Controlled Trial Number (ISRCTN) register (ISRCTN16962255) and clinicaltrials.gov (NCT03690050).

Patient consent for publication

Not applicable

Patient and Public Involvement

At the very early stages of the DIAMONDS trial conception, a DIAMONDS Patient and Public Involvement (PPI) group was established with the help of the Northern Ireland branch of DIABETES UK. The DIAMONDS PPI group comprised people living with diabetes and DMO, including a large group of members of the "Diabetes Family" Facebook group. The DIAMONDS PPI group contributed to the trial design and the research question, including the selection of outcomes, preparation of patient related materials for the trial, recruitment strategies, interpretation of trial results, and

For peer review only

preparation of the plain English summary. They also have a major role in the dissemination and implementation of trial results.

For peer review only

References

1. Yau JW, Rogers SL, Kawasaki R, Lamoureux EL, Kowalski JW, Bek T, et al. Global prevalence and major risk factors of diabetic retinopathy. Diabetes care. 2012;35(3):556-64.
2. Diabetes UK. Number of people with diabetes reaches 4.7 million London2019 [Available from: https://www.diabetes.org.uk/about_us/news/new-stats-people-living-with-diabetes.
3. Stitt AW, Curtis TM, Chen M, Medina RJ, McKay GJ, Jenkins A, et al. The progress in understanding and treatment of diabetic retinopathy. Progress in retinal and eye research. 2016;51:156-86.
4. National Institute for Health and Care Excellence. Ranibizumab for treating diabetic macular oedema (rapid review of technology appraisal guidance 237). London: NICE; 2013 April.
5. National Institute for Health and Care Excellence. Aflibercept for treating diabetic macular oedema. London: NICE; 2015 July.
6. National Institute for Health and Care Excellence. Faricimab for treating diabetic macular oedema. London: NICE; 2022 June.
7. Lois N, Campbell C, Waugh N, Azuara-Blanco A, Maredza M, Mistry H, et al. DIAbetic Macular Oedema aNd Diode Subthreshold micropulse laser (DIAMONDS): A pragmatic, double-masked, randomised, non-inferiority trial. Ophthalmology. in press.
8. Lois N, Campbell C, Waugh N, Azuara-Blanco A, Maredza M, Mistry H, et al. DIAbetic Macular Oedema aNd Diode Subthreshold micropulse laser (DIAMONDS): A pragmatic, multicentre, allocation concealed, double-masked prospective, randomised, non-inferiority, clinical trial. Health Technology Assessment. in press.
9. Mistry H, Waugh N, Lois N. DIAMONDS Health Economics Analysis Plan v1.5 2022 [Available from: https://nictu.hscni.net/diamonds-trial-documents/.
10. Lois N, Gardner E, Waugh N, Azuara-Blanco A, Mistry H, McAuley D, et al. Diabetic macular oedema and diode subthreshold micropulse laser (DIAMONDS): study protocol for a randomised controlled trial. Trials. 2019;20(1):1-11.
11. National Institute for Health and Care Excellence. Guide to the methods of technology appraisal. London: NICE; 2013.
12. Curtis L, Burns A. Unit Costs of Health and Social Care 2020. Kent: Personal Social Services Research Unit; 2020.
13. Department of Health. NHS reference costs 2018 to 2019: GOV.UK; 2018 [Available from: https://www.england.nhs.uk/publication/2018-19-national-cost-collection-data-publication/.
14. British Medical Association and Royal Pharmaceutical Society of Great Britain. British National Formulary No. 80, September 2020-March 2021. London: BMA and PRS.
15. Van Hout B, Janssen M, Feng Y-S, Kohlmann T, Busschbach J, Golicki D, et al. Interim scoring for the EQ-5D-5L: mapping the EQ-5D-5L to EQ-5D-3L value sets. Value in Health. 2012;15(5):708-15.
16. Mangione CM, Lee PP, Gutierrez PR, Spritzer K, Berry S, Hays RD. Development of the 25-list-item national eye institute visual function questionnaire. Archives of ophthalmology. 2001;119(7):1050-8.
17. Misajon R, Hawthorne G, Richardson J, Barton J, Peacock S, Iezzi A, et al. Vision and quality of life: the development of a utility measure. Investigative ophthalmology & visual science. 2005;46(11):4007-15.
18. Peacock S, Misajon R, Iezzi A, Richardson J, Hawthorne G, Keeffe J. Vision and quality of life: development of methods for the VisQoL vision-related utility instrument. Ophthalmic epidemiology. 2008;15(4):218-23.
19. Little RJ, Rubin DB. Statistical analysis with missing data: John Wiley & Sons; 2019.

For peer review only

Supplemental Tables

Supplemental Table A1: Unit costs

<table>
<tr><th>Resource Item</th><th>Unit Cost (£, 2020)</th><th colspan="2">Measurement unit</th><th>Source</th></tr>
<tr><td colspan="5">Staff Costs</td></tr>
<tr><td>Consultant</td><td>114</td><td colspan="2">per working hour</td><td>PSSRU 2020, page 158</td></tr>
<tr><td>Associate specialist/Staff Grade</td><td>117</td><td colspan="2">per working hour</td><td>PSSRU 2020, page 158</td></tr>
<tr><td>Retina Fellow</td><td>50</td><td colspan="2">per working hour</td><td>PSSRU 2019, page 158</td></tr>
<tr><td>Ophthalmic photographer/ Imaging technician</td><td>52</td><td colspan="2">per working hour</td><td>PSSRU 2020, page 148</td></tr>
<tr><td colspan="5">Anti-vascular endothelial growth factor costs</td></tr>
<tr><td>Ranibizumab</td><td>569</td><td colspan="2">Per dose</td><td>NHS Reference Costs 2019-20</td></tr>
<tr><td>Aflibercept</td><td>634</td><td colspan="2">Per dose</td><td>NHS Reference Costs 2019-20</td></tr>
<tr><td>Bevacizumab</td><td>277</td><td colspan="2">Per dose</td><td>NHS Reference Costs 2019-20</td></tr>
<tr><td colspan="5">Laser equipment costs</td></tr>
<tr><td>Laser Type</td><td>Current Cost[a]</td><td>Lifespan (years)</td><td>Total annual discounted costs</td><td>Cost per patient[b]</td></tr>
<tr><td>Complete scanning Laser Module TxCell/Haag Streit Fit/ IQ577nm with Micropulse®[c]</td><td>£79,800 - purchase price
£6,990 – total cost for a 5-year preventative maintenance contract (with the first 2 years being warranty) plus VAT</td><td>14</td><td>£8,860</td><td>£2.95</td></tr>
<tr><td>Nidek GYC-1000 Laser (including installation)[d]</td><td>£14,090 - purchase price
£3,653 - maintenance over 5 years</td><td>7</td><td>£3,113</td><td>£1.04</td></tr>
<tr><td>Pascal Laser[e]</td><td>£51,522 - purchase price (excluding optional extras)
£1,548 - maintenance cost</td><td>14</td><td>£6,266</td><td>£2.09</td></tr>
<tr><td>Argon laser (new design high quality ophthalmic laser)</td><td>£14,000 – purchase price
£809 - maintenance costs</td><td>7</td><td>£3,010</td><td>£1.03</td></tr>
<tr><td colspan="5">[a] When required, costs were inflated to 2019/20 prices using the NHS Cost Inflation Index (NHSCII). [b] Annual throughput estimate was 3,000: (Personal communication, Noemi Lois, email dated 21 April 2021).[c] Equipment price quotation: Carleton Ltd (via email on 25 July 2016). [d] Equipment price quotation: Birmingham Optical (via email on 25 July 2016). [e] Equipment price quotation: Topcon Ireland Medical (via email on 23 April 2021)
NHS: National Health Service</td></tr>
</table>

For peer review only

Supplemental Table A2: VisQol scores at baseline, 12 months and 24 months post-randomisation by VisQol dimension and trial group

Timepoint	VisQol Dimension	Subthreshold Micropulse laser (N = 133)		Standard threshold laser (N = 132)		Between-Group Difference (95% CI)	
		n	Unadjusted mean (SD)	n	Unadjusted mean (SD)		p-value
Baseline	Injure	130	0.953 (0.14)	116	0.967 (0.093)	-0.023 (-0.007 to 0.053)	0.141
	Cope	130	0.942 (0.109)	116	0.956 (0.119)	0.001 (-0.025 to 0.028)	0.915
	Friendships	130	0.915 (0.147)	116	0.940 (0.143)	0.003 (-0.031 to 0.037)	0.879
	Assistance	131	0.970 (0.093)	116	0.983 (0.080)	-0.001 (-0.023 to 0.02)	0.904
	Roles	131	0.927 (0.181)	116	0.961 (0.117)	0.025 (-0.007 to 0.057)	0.119
	Confidence	131	0.936 (0.158)	116	0.970 (0.075)	0.015 (-0.012 to 0.041)	0.269
12 months	Injure	130	0.975 (0.082)	109	0.950 (0.177)	-0.018 (-0.05 to 0.014)	0.272
	Cope	130	0.943 (0.105)	109	0.957 (0.121)	-0.007 (-0.036 to 0.021)	0.614
	Friendships	130	0.918 (0.141)	109	0.944 (0.143)	-0.008 (-0.045 to 0.028)	0.650
	Assistance	129	0.969 (0.117)	110	0.979 (0.086)	-0.007 (-0.030 to 0.015)	0.528
	Roles	129	0.953 (0.121)	110	0.966 (0.083)	-0.005 (-0.039 to 0.029)	0.769
	Confidence	129	0.950 (0.122)	110	0.963 (0.076)	-0.005 (-0.033 to 0.023)	0.710
24 months	Injure	113	0.985 (0.047)	112	0.955 (0.159)	0.004 (-0.029 to 0.037)	0.804
	Cope	112	0.963 (0.099)	111	0.956 (0.159)	0 (-0.030 to 0.028)	0.963
	Friendships	130	0.918 (0.127)	111	0.939 (0.136)	-0.005 (-0.042 to 0.032)	0.805
	Assistance	112	0.991 (0.043)	113	0.977 (0.138)	-0.002 (-0.0248 to 0.021)	0.880
	Roles	113	0.966 (0.113)	113	0.953 (0.138)	-0.012 (-0.047 to 0.022)	0.478
	Confidence	113	0.975 (0.046)	112	0.954 (0.121)	-0.009 (-0.037 to 0.020)	0.546

Injure: Likely to injure self; Cope: Coping with life demands; Friendships: Ability to have friendships; Assistance: Organising assistance; Roles: Difficult to fulfil roles; Confidence: Confidence to join activities.
CI: confidence interval.

For peer review only

Supplemental Table A3: NEI-VFQ-25 Subscale and Composite Scores in participants treated with micropulse subthreshold laser vs. standard threshold laser

Trial group by timepoint	Variable	Observations	Mean	Standard deviation	Minimum	Maximum
**Subthreshold Micropulse laser**						
Baseline	*NEI-VFQ-25 Composite Score*	*131*	*86.38*	*13.83*	*30.88*	*100*
	General Health	131	46.95	25.39	0.00	100
	General Vision	130	72.15	13.75	40.00	100
	Ocular Pain	131	85.02	20.65	0.00	100
	Near Activities	130	80.00	19.84	8.33	100
	Distance Activities	130	87.82	16.09	16.67	100
	Vision Social Function	130	94.62	13.56	37.50	100
	Vision Mental Health	131	82.16	19.95	18.75	100
	Vision Role Difficulties	130	83.85	23.11	0.00	100
	Vision Dependency	131	92.68	19.28	8.33	100
	Driving	92	92.84	12.08	33.33	100
	Color Vision	129	96.71	11.85	50.00	100
	Peripheral Vision	130	88.85	18.95	25.00	100
12 months	*NEI-VFQ-25 Composite Score*	*113*	*89.61*	*9.99*	*45.92*	*100*
	General Health	113	50.00	23.62	0.00	100
	General Vision	112	74.11	13.05	40.00	100
	Ocular Pain	113	89.82	17.40	12.50	100
	Near Activities	113	83.67	19.30	8.33	100
	Distance Activities	112	90.29	15.06	33.33	100
	Vision Social Function	113	96.68	8.35	50.00	100
	Vision Mental Health	113	86.06	15.38	18.75	100
	Vision Role Difficulties	112	86.27	20.95	0.00	100
	Vision Dependency	113	96.31	12.35	25.00	100
	Driving	77	94.53	9.57	50.00	100
	Color Vision	113	98.45	8.37	25.00	100
	Peripheral Vision	112	93.08	14.32	50.00	100
24 months	*NEI-VFQ-25 Composite Score*	*114*	*87.19*	*14.08*	*22.65*	*100*
	General Health	114	52.85	29.33	0.00	100
	General Vision	114	72.63	15.57	20.00	100
	Ocular Pain	114	88.38	16.61	25.00	100
	Near Activities	114	81.18	21.55	0.00	100
	Distance Activities	114	87.35	17.57	12.50	100
	Vision Social Function	113	95.24	12.87	25.00	100
	Vision Mental Health	113	83.13	20.22	0.00	100
	Vision Role Difficulties	113	85.07	24.31	0.00	100
	Vision Dependency	111	93.09	19.72	0.00	100
	Driving	81	91.82	13.57	16.67	100
	Color Vision	110	97.73	9.90	50.00	100
	Peripheral Vision	113	88.50	20.60	25.00	100
**Standard threshold laser**						

For peer review only

Baseline	*NEI-VFQ-25 Composite Score*	*130*	*87.00*	*12.73*	*44.63*	*100*
	General Health	130	51.92	25.22	0.00	100
	General Vision	130	72.92	15.52	40.00	100
	Ocular Pain	130	85.38	17.82	25.00	100
	Near Activities	130	80.16	19.33	25.00	100
	Distance Activities	130	89.01	14.17	41.67	100
	Vision Social Function	130	93.94	13.68	25.00	100
	Vision Mental Health	130	80.87	20.62	6.25	100
	Vision Role Difficulties	129	82.75	26.48	0.00	100
	Vision Dependency	130	93.72	14.57	33.33	100
	Driving	91	95.05	9.38	50.00	100
	Color Vision	130	96.35	11.69	25.00	100
	Peripheral Vision	130	91.54	16.63	25.00	100
12 months	*NEI-VFQ-25 Composite Score*	*117*	*88.47*	*13.78*	*29.21*	*100*
	General Health	116	49.78	25.00	0.00	100
	General Vision	116	75.17	14.59	40.00	100
	Ocular Pain	117	88.25	15.25	37.50	100
	Near Activities	117	83.62	19.29	8.33	100
	Distance Activities	117	88.89	17.02	25.00	100
	Vision Social Function	116	94.29	14.22	25.00	100
	Vision Mental Health	117	86.38	18.99	6.25	100
	Vision Role Difficulties	117	85.26	24.27	0.00	100
	Vision Dependency	116	92.74	19.37	0.00	100
	Driving	81	93.26	14.65	33.33	100
	Color Vision	115	98.48	6.86	50.00	100
	Peripheral Vision	115	91.96	16.07	25.00	100
24 months	*NEI-VFQ-25 Composite Score*	*115*	*88.80*	*13.78*	*29.08*	*100*
	General Health	115	51.96	24.59	0.00	100
	General Vision	114	74.39	14.94	20.00	100
	Ocular Pain	115	89.35	15.55	37.50	100
	Near Activities	115	82.79	20.03	16.67	100
	Distance Activities	115	89.53	17.41	16.67	100
	Vision Social Function	115	94.57	13.05	12.50	100
	Vision Mental Health	115	85.43	21.26	0.00	100
	Vision Role Difficulties	114	86.73	21.76	0.00	100
	Vision Dependency	114	94.01	19.03	0.00	100
	Driving	78	96.42	7.35	58.33	100
	Color Vision	115	96.52	11.89	25.00	100
	Peripheral Vision	115	91.96	16.74	25.00	00

For peer review only

Supplemental Table A4: Frequency of laser treatments and use of anti-VEGFs or steroids as rescue treatment over the 24-month follow-up period

	Subthreshold Micropulse Laser	Standard Threshold Laser	Difference (95% CI)	P-value
**Number of laser treatments used from baseline to month 24 in study eye [a b]**	2.37 (0.11) n=133	1.89 (0.11) n=132	0.48 (0.18, 0.79)	0.002
**Number of patients with at least one steroid injection in study eye (as additional treatment) from baseline to month 24[c]**	0 (0.0%)	1 (0.8%)		
**Number of patients receiving at least one anti-VEGF treatment (as additional treatment) from baseline to month 24[c]**	24 (18.1%) n=133	28 (21.2%) n=132	OR: 0.78 (0.42 – 1.45)	0.44
			% point difference: -2.8 (-13.1 – 7.5)	0.59
**Number of anti-VEGF treatments (as additional treatment) from baseline to month 24[a d]**	0.80 (0.23) n=133	1.30 (0.23) n=132	-0.50 (-1.14 – 0.14)	0.13
**Number of anti-VEGF treatments (as additional treatment) from baseline to month 24 [d e]**				
**1-2**	4 (16.7%) n=24	7 (25.0%) n=28		
**3-4**	10 (41.7%) n=24	7 (25.0%) n=28		
**5-10**	10 (41.7%) n=24	9 (32.1%) n=28		
**>10**	0 (0.0%) n=24	5 (17.9%) n=28		

[a] Mean (SE) presented for continuous outcomes [b] Number of laser treatments, were analysed using linear regression with adjustment for baseline BCVA and minimisation variables. [c] number of patients receiving at least one additional treatment (defined as at least one anti-VEGF or steroid), were analysed using logistic regression models with adjustment for the minimisation variables. [d] Number of steroid injections and number of anti-VEGF treatments (as mean and number (%) in categories), were analysed using linear regression with adjustment for minimisation variables. [e] n (%) based on no. of patients receiving anti VEGF treatments.

anti-VEGF: anti-vascular endothelial growth factor; CI: confidence interval; OR: odds ratio; RR: risk ratio

CHEERS 2022 Checklist

	Item	Guidance for Reporting	Reported in section
**TITLE**			
Title	1	Identify the study as an economic evaluation and specify the interventions being compared.	1
**ABSTRACT**			
Abstract	2	Provide a structured summary that highlights context, key methods, results and alternative analyses.	4
**INTRODUCTION**			
Background and objectives	3	Give the context for the study, the study question and its practical relevance for decision making in policy or practice.	6
**METHODS**			
Health economic analysis plan	4	Indicate whether a health economic analysis plan was developed and where available.	6
Study population	5	Describe characteristics of the study population (such as age range, demographics, socioeconomic, or clinical characteristics).	6
Setting and location	6	Provide relevant contextual information that may influence findings.	6
Comparators	7	Describe the interventions or strategies being compared and why chosen.	6
Perspective	8	State the perspective(s) adopted by the study and why chosen.	6
Time horizon	9	State the time horizon for the study and why appropriate.	6
Discount rate	10	Report the discount rate(s) and reason chosen.	7
Selection of outcomes	11	Describe what outcomes were used as the measure(s) of benefit(s) and harm(s).	8-9
Measurement of outcomes	12	Describe how outcomes used to capture benefit(s) and harm(s) were measured.	8-9
Valuation of outcomes	13	Describe the population and methods used to measure and value outcomes.	8-9
Measurement and valuation of resources and costs	14	Describe how costs were valued.	8
Currency, price date, and conversion	15	Report the dates of the estimated resource quantities and unit costs, plus the currency and year of conversion.	7
Rationale and description of model	16	If modelling is used, describe in detail and why used. Report if the model is publicly available and where it can be accessed.	N/A
Analytics and assumptions	17	Describe any methods for analysing or statistically transforming data, any extrapolation methods, and approaches for validating any model used.	9
Characterizing heterogeneity	18	Describe any methods used for estimating how the results of the study vary for sub-groups.	N/A
Characterizing distributional effects	19	Describe how impacts are distributed across different individuals or adjustments made to reflect priority populations.	N/A
Characterizing uncertainty	20	Describe methods to characterize any sources of uncertainty in the analysis.	N/A
Approach to engagement with patients and others affected by the study	21	Describe any approaches to engage patients or service recipients, the general public, communities, or stakeholders (e.g., clinicians or payers) in the design of the study.	N/A
**RESULTS**			
Study parameters	22	Report all analytic inputs (e.g., values, ranges, references) including uncertainty or distributional assumptions.	1 (Suppl A1)
Summary of main results	23	Report the mean values for the main categories of costs and outcomes of interest and summarise them in the most appropriate overall measure.	10-13
Effect of uncertainty	24	Describe how uncertainty about analytic judgments, inputs, or projections affect findings. Report the effect of choice of discount rate and time horizon, if applicable.	N/A
Effect of engagement with patients and others affected by the study	25	Report on any difference patient/service recipient, general public, community, or stakeholder involvement made to the approach or findings of the study	N/A
**DISCUSSION**			
Study findings, limitations, generalizability, and current knowledge	26	Report key findings, limitations, ethical or equity considerations not captured, and how these could impact patients, policy, or practice.	13-14
**OTHER RELEVANT INFORMATION**			
Source of funding	27	Describe how the study was funded and any role of the funder in the identification, design, conduct, and reporting of the analysis	2
Conflicts of interest	28	Report authors conflicts of interest according to journal or International Committee of Medical Journal Editors requirements.	2

Husereau D, Drummond M, Augustovski F, de Bekker-Grob E, Briggs AH, Carswell C, Caulley L, Chaiyakunapruk N, Greenberg D, Loder E, Mauskopf J, Mullins CD, Petrou S, Pwu RF, Staniszewska S; CHEERS 2022 ISPOR Good Research Practices Task Force. Consolidated Health Economic Evaluation Reporting Standards 2022 (CHEERS 2022) Statement: Updated Reporting Guidance for Health Economic Evaluations. BMJ. 2022;376:e067975.
The checklist is Open Access distributed in accordance with the terms of the Creative Commons Attribution (CC BY 4.0) license, which permits others to distribute, remix, adapt and build upon this work, for commercial use, provided the original work is properly cited. See: http://creativecommons.org/licenses/by/4.0/.

For peer review only - http://bmjopen.bmj.com/site/about/guidelines.xhtml

BMJ Open

Subthreshold micropulse laser versus standard laser for the treatment of central-involving diabetic macular oedema with central retinal thickness of <400µ: a cost-effectiveness analysis from the DIAMONDS trial

Journal:	*BMJ Open*
Manuscript ID	bmjopen-2022-067684.R1
Article Type:	Original research
Date Submitted by the Author:	17-Aug-2023
Complete List of Authors:	Mistry, Hema; University of Warwick; University Hospitals Coventry and Warwickshire NHS Trust<br>Maredza, Mandy ; University of Warwick<br>Campbell, Christina; Northern Ireland Clinical Trials Unit<br>Lois, Noemi; Wellcome-Wolfson Institute for Experimental Medicine, Ophthalmology
**Primary Subject Heading**:	Health economics
Secondary Subject Heading:	Ophthalmology
Keywords:	OPHTHALMOLOGY, Diabetic retinopathy < DIABETES & ENDOCRINOLOGY, HEALTH ECONOMICS

SCHOLARONE™
Manuscripts

I, the Submitting Author has the right to grant and does grant on behalf of all authors of the Work (as defined in the below author licence), an exclusive licence and/or a non-exclusive licence for contributions from authors who are: i) UK Crown employees; ii) where BMJ has agreed a CC-BY licence shall apply, and/or iii) in accordance with the terms applicable for US Federal Government officers or employees acting as part of their official duties; on a worldwide, perpetual, irrevocable, royalty-free basis to BMJ Publishing Group Ltd ("BMJ") its licensees and where the relevant Journal is co-owned by BMJ to the co-owners of the Journal, to publish the Work in this journal and any other BMJ products and to exploit all rights, as set out in our licence.

The Submitting Author accepts and understands that any supply made under these terms is made by BMJ to the Submitting Author unless you are acting as an employee on behalf of your employer or a postgraduate student of an affiliated institution which is paying any applicable article publishing charge ("APC") for Open Access articles. Where the Submitting Author wishes to make the Work available on an Open Access basis (and intends to pay the relevant APC), the terms of reuse of such Open Access shall be governed by a Creative Commons licence – details of these licences and which Creative Commons licence will apply to this Work are set out in our licence referred to above.

Other than as permitted in any relevant BMJ Author's Self Archiving Policies, I confirm this Work has not been accepted for publication elsewhere, is not being considered for publication elsewhere and does not duplicate material already published. I confirm all authors consent to publication of this Work and authorise the granting of this licence.

For peer review only

Subthreshold micropulse laser versus standard laser for the treatment of central-involving diabetic macular oedema with central retinal thickness of <400μ: a cost-effectiveness analysis from the DIAMONDS trial

Hema Mistry [1,5*], Mandy Maredza[2], Christina Campbell [3], Noemi Lois[4] on behalf of the DIAMONDS study group.

Affiliations

[1] Warwick Clinical Trials Unit, Warwick Medical School, University of Warwick, Coventry, CV4 7AL, UK.

[2] Division of Health Sciences, Warwick Medical School, University of Warwick, Coventry, CV4 7AL, UK.

[3] Northern Ireland Clinical Trials Unit, 7 Lennoxvale, Belfast, BT9 5BY, UK.

[4] Wellcome-Wolfson Institute for Experimental Medicine, Queen's University Belfast, 97 Lisburn Road, Belfast, BT9 7BL, UK.

[5]University Hospitals Coventry and Warwickshire NHS Trust, Clifford Bridge Road, Coventry, CV2 2DX, UK

Correspondence to: Dr Hema Mistry, Warwick Clinical Trials Unit, Warwick Medical School, University of Warwick, Coventry, UK; Email: Hema.Mistry@warwick.ac.uk; Tel: +44 (0)2476 151183

Key words:

Ophthalmology, Diabetic retinopathy, Diabetes & Endocrinology, Health Economics

For peer review only

Abstract (currently 297 words, 300 words max)

Objectives

To estimate the economic costs, health-related quality of life outcomes and cost-effectiveness of subthreshold micropulse laser (SML) versus standard laser (SL) for the treatment of diabetic macular oedema (DMO) with central retinal thickness (CRT) of <400µ.

Design

An economic evaluation was conducted within a pragmatic, multicentre, randomised clinical trial, DIAMONDS.

Setting

18 UK Hospital Eye Services.

Participants

Adults with diabetes and centre involving DMO with CRT <400µ.

Interventions

Participants (n=266) were randomised 1:1 to receive SML or SL.

Methods

The base-case used an intention-to-treat approach conducted from a UK National Health Service (NHS) and personal social services (PSS) perspective. Costs (2019–2020 prices) were collected prospectively over the 2-year follow-up period. A bivariate regression of costs and quality-adjusted life-years (QALYs), with multiple imputation of missing data, was conducted to estimate the incremental cost per QALY gained and the incremental net monetary benefit of SML in comparison to SL. Sensitivity analyses explored uncertainty and heterogeneity in cost-effectiveness estimates.

Results

One participant in the SL arm withdrew consent for data to be used; data from the remaining 265 participants were included in analyses. Mean (standard error [SE]) NHS and PSS costs over 24 months were £735.09 [£111.85] in the SML arm versus £1099.70 [£195.40] in the SL arm (p=0.107). Mean (SE) QALY estimates were 1.493 [0.024] versus 1.485 [0.020], respectively (p=0.780), giving an

For peer review only

insignificant difference of 0.008 QALYs. The probability SML is cost-effective at a threshold of £20,000 per QALY was 76%.

Conclusions

There were no statistically significant differences in EQ-5D-5L scores or costs between SML and SL. Given these findings and the fact that SML does not burn the retina, unlike SL, and has equivalent efficacy to SL, it may be preferred for the treatment of people with DMO with CRT <400μ.

Trial registration number

ISRCTN17742985; NCT03690050

For peer review only

Strengths and limitations of this study

- Study is based on a high-quality randomised clinical trial.
- For people with diabetic macular oedema (DMO) and central retinal thickness <400μ, subthreshold micropulse laser (SML) is equivalent in efficacy to standard laser (SL).
- First study to compare the cost-effectiveness of SML with that of SL for the treatment of DMO.
- Low rate of missing data for both costs and outcomes.
- We used published costs of anti-VEGF drugs, rather than NHS costs which incorporates confidential price discounts for these drugs.

For peer review only

Manuscript word count 3309 words (including the 3 tables); max 4000 words; up to 5 images/tables

Introduction

Diabetic macular oedema (DMO) is a visual threatening complication of diabetes, occurring in approximately 7% of people living with diabetes.(1) Given the high and continuously increasing prevalence of diabetes,(2) DMO is a frequent eye disease requiring treatment in ophthalmic clinics in the UK and worldwide. In DMO fluid accumulates in the center of the retina, the macula, which is the area of the retina responsible for providing central vision.(3) The purpose of the treatment is to restore the anatomy of the macula by clearing up this fluid and restoring vision. Treatments include intraocular injections of anti-vascular endothelial growth factor (anti-VEGF) drugs or steroids and macular laser. The National Institute for Health and Care Excellence (NICE) recommends anti-VEGF therapy for people with more severe forms of DMO, with central retinal (macular) thickness (CRT) of 400µ or above, as measured in scans obtained using an imaging modality called optical coherence tomography (OCT).(4-6) For milder forms of DMO (CRT <400µ) NICE recommends macular laser. Intravitreal steroids are advised for people that do not respond to the above-mentioned therapies.

DIAbetic Macular Oedema aNd Diode Subthreshold micropulse laser (DIAMONDS) was a pragmatic, allocation-concealed, double-masked, multicentre, randomised, non-inferiority clinical trial which compared the clinical effectiveness and cost-effectiveness of subthreshold micropulse laser (SML) and standard laser (SL) for the treatment of people with DMO with CRT of <400µ. DIAMONDS participants were randomised 1:1 to receive SML (577 nm) or SL (e.g. using argon, frequency-doubled neodymium-doped yttrium aluminium garnet [Nd:YAG] 532 nm laser). Laser treatment could be repeated as needed, using the allocated laser at randomisation and rescue treatment with anti-VEGFs or steroids was allowed. The primary outcome was the mean change in best-corrected visual acuity (BCVA) in the study eye at 24 months. DIAMONDS found SML and SL to have equivalent clinical efficacy.(7, 8) This finding is clinically important given the fact that SML, unlike SL, does not

For peer review only

cause any functional or structural damage to the retina(9-11) and, thus, may be preferred by patients and doctors. Herein, we present the detailed within-trial economic evaluation comparing costs and benefits of the two laser modalities, SML and SL.

Methods

Patient and Public Involvement

At the very early stages of the DIAMONDS trial conception, a DIAMONDS Patient and Public Involvement (PPI) group was established with the help of the Northern Ireland branch of DIABETES UK. The DIAMONDS PPI group comprised people living with diabetes and DMO, including a large group of members of the "Diabetes Family" Facebook group. The DIAMONDS PPI group contributed to the trial design and the research question, including the selection of outcomes, preparation of patient related materials for the trial, recruitment strategies, interpretation of trial results, and preparation of the plain English summary. They also have a major role in the dissemination and implementation of trial results.

The study is reported as per Consolidated Health Economic Evaluation Reporting Standards (CHEERS) 2022 Statement.(12) As detailed in the Health Economics Analysis Plan (HEAP)(13) we planned to conduct a within-trial analysis comparing the cost-effectiveness of SML with SL. The protocol for the DIAMONDS trial envisaged that economic modelling might be required if visual outcomes differed between arms.(14) The DIAMONDS protocol was designed to minimise visual loss in participants and, thus, repeating laser treatment or undertaking rescue treatment with intravitreal anti-VEGF drugs and/or steroids if criteria for rescue were met, were allowed in either arm of the trial. DIAMONDS recruited 266 participants, 116 (87%) in the SML arm and 115 (86%) in the SL arm had primary outcome data, fulfilling the requirements of the power calculation (113 participants with BCVA data at month 24 were required). DIAMONDS found SML and SL to be equivalent in terms of the primary outcome.(7, 8) Hence, economic modelling was not required. Herein, we report detailed

For peer review only

economic costs, health-related quality of life (HRQoL) outcomes and cost-effectiveness of SML versus SL.

The economic evaluation took the form of a cost-utility analysis, expressed in terms of cost per quality-adjusted life-year (QALY) gained. The study was conducted over the 24-month time horizon of the trial. The primary analysis adopted a National Health Service (NHS) and personal and social service (PSS) perspective. Costs and outcomes in the second year of follow-up were discounted at 3.5% in line with the NICE reference case.(15)

Resource use data were collected and reported on trial case record forms (CRFs) at scheduled clinic visits at 4, 8, 12, 16, 20 and 24 months. Data were collected on the costs of laser treatment, both at the initial laser session and at subsequent ones if required, outpatient visits, and intravitreal anti-VEGF and/or steroid treatment (costs of drugs and administration) if rescue was required. All costs were expressed in Great British pounds sterling and valued in 2019–20 prices. If costs were not in line, they were inflated to 2019-20 prices using the NHS Cost Inflation Index (NHSCII).(16)

The costs of laser treatment included staff and equipment costs (capital and maintenance costs of laser machines). Unit costs for staff were obtained from the Unit Costs of Health and Social Care 2019 compendium (16) and were based on the time it took to for each procedure to be undertaken. These times were recorded on CRFs and included: (i) the time taken to obtain fundus fluorescein angiography (FFA) and spectral domain optical coherence tomography (SD-OCTs) scans to guide laser treatment (if used) and (ii) time taken by ophthalmologists to perform the laser procedure, including the time for counselling the participant. Costs of laser machines were obtained from manufacturers. An annual equivalent cost of equipment was obtained by annuitising the capital costs of the item over its useful life span and applying a discount rate of 3.5% per annum. A per-patient cost of

For peer review only

equipment was estimated by assuming that the laser machines were used to treat 3,000 patients per year.

Data were also collected on any outpatient attendances or hospital admissions related to DMO or the treatments. Where an outpatient attendance was reported but no procedure undertaken, the average unit cost of an outpatient ophthalmology visit was used (varying between £80-£101 per consultation depending on whether the consultation was 'non-consultant' versus 'consultant-led').(17) The CRFs recorded data on the grade of professional that attended the patient, for example, if a consultant attended to the patient, then the consultant-led unit cost was applied. Where a procedure(s) was undertaken as part of the visit the relevant HRG code was derived using the HRG4+ Reference Costs Grouper Software (NHS Digital, Leeds, UK).

CRFs also recorded information on other tests or investigations, medication use including anti-VEGF/steroids or other rescue treatments. Anti-VEGF and steroid drugs were separately costed as these are considered an unbundled HRG. Costing of laser retreatments followed the same approach as costing for the index (first session) laser procedure.

Unit costs were derived from national compendia in accordance with NICE's Guide to the Methods of Technology Appraisal.(15) The key databases included the Department of Health and Social Care's Reference Costs 2018–19 schedules,(17) the PSSRU's Unit Costs of Health and Social Care 2020 compendium (16), 2020 volume of the British National Formulary.(18) Supplemental Table A1 gives a summary of the unit costs for resource use and the laser equipment. Resource inputs were valued by attaching unit costs.

The HRQoL of trial participants was assessed at baseline and at 12- and 24-months post-randomisation using the EQ-5D-5L instrument. The EQ-5D-5L questionnaire was used to generate

For peer review only

QALYs for the cost-effectiveness analysis. (19) The QALY is a measure that combines quantity and quality of life lived into a single metric, with one QALY equating to one year of full health. To convert EQ-5D-5L responses into health utility scores we used the EQ-5D-5L Crosswalk Index Value Calculator which maps the EQ-5D-5L descriptive system data onto the EQ-5D-3L valuation set. This valuation set was recommended by NICE at the time when the analysis was undertaken.(19)

HRQoL was also assessed using two vision specific measures: the National Eye Institute Visual Functioning Questionnaire - 25 (NEI-VFQ-25) and Vision and Quality of Life Index (VisQoL).(20-22) The NEI-VFQ-25 is a validated questionnaire that has been used widely to evaluate visual outcomes in patients with eye diseases including diabetic retinopathy and DMO. The VisQol questionnaire has not been widely validated but is shorter than the NEI-VFQ-25 with only six attributes: physical well-being, independence, social well-being, self-actualisation, planning and organisation. The utilities for VisQoL were developed using a time-trade off exercise in people who were visually impaired which included patients with age-related macular degeneration, diabetic retinopathy and glaucoma.(22)

Summary statistics were generated for resource use variables, health utility values and QALYs by treatment allocation and assessment time point. For resource use and costs, mean values were compared between groups using two sample t-tests. Differences between groups, along with 95% confidence intervals (CIs) were estimated using non-parametric bootstrap estimates (10,000 replications). For HRQoL, these were presented as mean values with their associated standard errors. Between-group differences were compared using the two-sample t-test.

Multiple imputation by chained equations was used to predict missing health status (utility) scores and costs based on the assumption that data were missing at random (MAR). Twenty imputed data sets were generated and used to inform the base-case analyses. Parameter estimates were pooled

For peer review only

across the 20 imputed data sets using Rubin's rules to account for between- and within-imputation components of variance terms associated with parameter estimates.(23)

The base-case cost-effectiveness analysis was performed using an intention-to-treat approach. Mean incremental costs and QALYs were estimated using seemingly unrelated regression (SUR) methods that account for the correlation between costs and outcomes. The SUR adjusted for covariates (baseline utilities, baseline body mass index (BMI), baseline best corrected distance visual acuity (BCVA), patient-reported previous use of anti-VEGF at baseline and previous use of macular laser).

Results

As reported elsewhere (7, 8) there was no difference in the clinical effectiveness of SML and SL. Table 1 shows that there were also no statistically significant differences between laser groups in EQ-5D-5L scores at baseline, 12 and 24 months.

For peer review only

Table 1: EQ-5D-5L utility scores at baseline, 12 and 24 months and QALYs (base-case, imputed analysis)

Variable	Subthreshold micropulse laser (n = 133)	Standard laser (n = 132)	Between-Group Difference (95% CI)	p-value
	Mean (SE)	Mean (SE)		
**EQ-5D Utility Scores***				
Baseline	0.758 (0.267)	0.772 (0.226)	-0.014 (-0.074 to 0.046)	0.640
12 months	0.767 (0.250)	0.758 (0.017)	0.009 (-0.041to 0.059)	0.717
24 months	0.739 (0.278)	0.743 (0.279)	-0.004 (-0.064 to 0.056)	0.897
**EQ-5D-5L QALYs**				
Over 2 years	1.493 (0.024)	1.485 (0.024)	0.008 (-0.061 to 0.075)	0.836

*Analysis adjusted for participant age, gender, baseline BCVA (Best Corrected Distance Visual Acuity) and participant's previous use of Anti-VEGF (Anti-Vascular Endothelial Growth Factor) and laser therapy at baseline, with repeated measures within participant and site.
SE = standard error; CI = confidence interval

Furthermore, there were no statistically significant differences between the two laser treatment groups for any of the VisQoL dimensions or NEI-VFQ-25 subscales at any follow-up time point (Supplemental Table A2 and Supplemental Table A3, respectively).

The mean numbers of laser treatments performed were 2.4 in the SML and 1.9 in the SL arm. This difference was statistically significant (p=0.002) (Supplemental Table A4), but equated to less than one further session of laser in the SML arm when compared with the SL arm during the 2-year trial. Furthermore, this difference was driven by a small number of participants requiring a higher number of lasers sessions in the SML group (13 participants required 6 or 7 laser treatments in the SML arm compared to 2 in the SL arm). Eighteen percent of participants in the SML arm and 21% in the SL arm required rescue treatments in the study eye (almost all with anti-VEGF drugs; in addition, only one participant had a steroid injection). The average numbers of anti-VEGF treatments per arm from

For peer review only

baseline to 24 months were 1.06 in the SML arm and 1.96 in the SL (Supplemental Table A4). The number in the SL arm was skewed by five participants who received 10 or more injections.

Table 2 summarises the NHS costs associated with resource use in the base-case (imputed) analysis by cost category and follow-up period. Costs of the first laser procedure are reported separately from those of subsequent laser retreatments. The total costs of laser therapy for each participant includes costs of the first laser procedure plus any subsequent laser retreatments they had. The mean (standard error (SE)) cost for the first laser procedure (including costs of performing OCT and FFA to guide laser treatment, if done) was £45.59 (1.64) for the SML compared with £42.29 (1.69) for the SL; the difference was not statistically significant(p=0.09). The mean total NHS and PSS costs were lower in SML compared with the SL (£735.09 vs. £1099.70) between baseline to 24 months post-randomisation; this difference was not statistically significant at the 5% level. The CI around total costs were wide and overlapped and the difference was driven by the higher number of anti-VEGF rescue injections.

Table 2: *Economic costs by trial allocation arm and cost component category for the entire follow-up period in base-case (imputed) analysis (£, 2019-20 prices)*

**Parameter**	**Subthreshold Micropulse laser**	**Standard threshold laser**	**Mean Difference**	**Bootstrap 95% CI**
	**Mean Costs (SE)**	**Mean Costs (SE)**		
Index Laser Procedure	45.59 (1.64)	42.29 (1.69)	3.31	(-1.33 to 7.95)
Laser retreatments	53.02 (5.17)	41.69 (4.42)	11.32	(-2.08 to 24.73)
Outpatient care	124.85 (22.56)	130.32 (31.58)	-5.47	(-81.92 to 70.97)
Anti-VEGF drug costs	511.63 (105.85)	885.40 (183.29)	-373.77	(-791.01 to 43.48)
**Total NHS and PSS Costs**	**735.09 (111.85)**	**1,099.70 (195.40)**	**-364.61**	**(-807.09 to 77.87)**
SE = standard error; CI = confidence interval				

For peer review only

Over the 2-year follow-up period, participants in the SML arm, compared to the SL arm, experienced a non-statistically significant increase in QALYs of 0.008 (circa 3 days of good quality of life). In addition, the mean NHS and PSS costs were lower in the SML arm compared to the SL arm (mean cost difference –£365) – see Table 3. The confidence interval for the cost difference was wide and ranged from cost saving to cost increasing. Although neither costs nor benefits were statistically significantly different between SML and SL, the incremental cost-effectiveness ratio (ICER) for the base-case imputed analysis indicated that SML dominates, as average costs for this intervention were slightly lower and average benefits marginally higher (but not significantly so) than those for SL. Assuming cost-effectiveness threshold of £20,000 per QALY, the probability that SML was cost-effective was 0.76, and the NMB associated with SML was positive. Figure 1 shows the joint distributions of costs and outcomes.

Table 3: Cost-effectiveness, cost/QALY (£, 2020): SML compared to SL

Mean incremental cost (95% CI)	Mean incremental QALY (95% CI)	ICER	Probability of cost-effectiveness*	Net Monetary Benefit (NMB)*
Base-case analysis - ITT approach: Imputed attributable costs and QALYs, covariate adjusted[a]				
-365 (-822 to 93)	0.008 (-0.059 to 0.075)	Dominant	0.763	520 (-925 to 1,965)
***Cost-effectiveness threshold is at £20,000/QALY threshold**<br>[a] Adjusted for baseline EQ-5D utility, BMI and minimisation variables at baseline (best corrected distance visual acuity and previous use of laser treatment)<br>CI = confidence interval; ICER = incremental cost-effectiveness ratio; ITT = intention-to-treat approach; QALY = quality-adjusted life year				

Discussion

DIAMONDS was a pragmatic clinical trial carried out in 16 NHS ophthalmology departments throughout the UK. The DIAMONDS trial was powered to detect not only differences in the primary outcome (BCVA) but also in important secondary outcomes (CRT and vision quality of life).(8) However, a limitation of this cost-effectiveness analysis is that we did not use BCVA as the primary outcome, instead we used QALYs as the main outcome. Participants were treated and followed as per routine clinical care. Costs and outcome data were collected prospectively. We found no

For peer review only

significant differences in EQ-5D-5L and only a trivial non-significant difference of 0.008 in a calculation of QALYs. So, the verdict on whether one form of laser is better than the other shifts the focus onto the costs. This analysis found that costs were slightly higher (but not statistically significantly so) in the SL arm compared to the SML arm, due to more participants in the SL arm needing higher numbers (10 or more) of anti-VEGF rescue injections. In economic terms bringing both the costs and QALYs together in a ratio form, this meant the ICER for the base-case analysis indicated that SML is the dominant procedure, as average costs for this intervention were lower and average benefits were marginally higher than those for SL. However, caution should be taken when interpreting the results, given the wide confidence intervals around the mean costs (which ranges from cost saving to cost increasing). Taking this into consideration, costs of SML and SL treatment arms seem comparable. We also conducted a per-protocol analysis as part of a sensitivity analysis as DIAMONDS was a non-inferiority trial. The results were in line with the intention-to-treat analysis, were SML remained the dominant option and the probability of of SML being cost-effectiveness at the £20k/QALY threshold was 0.773.

As noted in the main clinical effectiveness manuscript(7) there may be advantages of SML over SL. , Among these and importantly, the fact that SML does not cause any functional or structural damage to the retina(9-11) and, thus, can be repeated as needed. This is an advantage for patients as their retina will remain intact despite the application of SML but would lose cells as a result of the laser burn if SL is applied. Furthermore, the lack of a burn following SML makes the delivery of the treatment safer in less experienced hands as, unlike SL, it does not carry a risk of burning the fovea. Furthermore, for the same reason, it may allow training of allied non-medical staff to undertake this procedure, who currently, are already undertaking anti-VEGF injections in the UK and other parts of the world. This would help alleviate clinical capacity constraints experienced by most ophthalmology units across the world. The clinical effectiveness results reported previously(7, 8) and the HRQoL results presented here showed that SML is as clinically effective as SL at a similar cost.

For peer review only

In DIAMONDS, two vision-specific patient reported outcome quality of life instruments were used in addition to the generic preference-based EQ-5D-5L. Neither showed any statistically significant differences between laser arms. It has sometimes been mentioned that changes in vision that are sufficient to affect some activities of daily living, may not be reflected in changes in EQ-5D, because the EQ-5D may not be as sensitive to detect changes in quality of life as other visual specific questionnaires such as the NEI-VFQ-25.(4, 24, 25) A mapping exercise from DIAMONDS data is underway to explore this important aspect.

One limitation in the current analysis is the fact that we had to use published costs of anti-VEGF drugs (at list prices, these can range from £5 to £7k a year),(4-6) rather than costs to the NHS. We know that there are confidential price discounts for these drugs when used in the NHS (without taking into account the administration of the injection into the eye and monitoring visits) and this will most likely reduce the cost differential between the two arms.(4-6) Another limitation is that DIAMONDS included only patients with CRT <400µ, so extrapolations with regard to the cost-effectiveness of SML when compared with SL in thicker retinas, where standard laser is known to be less effective, cannot be made. Furthermore, the analysis was conducted from a NHS and PSS perspective, so we have not taken into account any broader societal costs such as time off work or care for children or other dependents, when patients have laser treatment.

Both SML and SL were successful in 80% of participants.(7) Macular laser treatment is known to be less expensive than anti-VEGF drugs and may be more convenient and acceptable to participants, although no trials have been conducted comparing the cost-effectiveness of macular laser with anti-VEGFs in people with <400µ CRT DMO. Thus, SML should be considered as first line therapy for patients with central involving DMO with CRT of <400µ.

For peer review only

Contributor statement/Acknowledgements

The Authors would like to thank all the DIAMONDS participants and the DIAMONDS patient and public involvement (PPI) group. We also thank Dr Pamela Royle for literature searches. We would also like to thank Iridex for providing a free loan for the laser equipment for the trial and William Moore, Joan Stauffer, George Marcellino (*in memoriam*), Gareth Hymas, Nick Fitrzyk, and Suzanne Kelly, for their support. Iridex had no role in the design, data analysis or interpretation of the DIAMONDS data or in the writing of this report. We thank also the NIHR HTA programme for providing funding for the trial and note that the views expressed in this paper are those of the authors and not necessarily those of the NHS, the NIHR or the Department of Health and Social Care.

Author contributions

Hema Mistry contributed to the design of the health economics plan and provided oversight of all aspects of the economic evaluation including its design, conduct, analysis and reporting of this paper.

Mandy Maredza (University of Warwick) conducted the analysis and reporting of the economic evaluation and contributed to the drafting of this paper.

Christina Campbell (Trial Statistician, NICTU) performed the statistical analyses, assisted with interpretation of data and contributed to the drafting of this paper.

Professor Noemi Lois (Clinical Professor of Ophthalmology, Wellcome-Wolfson Institute for Experimental Medicine, Queen's University Belfast) is the Chief Investigator for the DIAMONDS trial conceived the trial, with input from the DIAMONDS Study Group, and led it to its successful completion. In addition, Prof Lois contributed with the identification, recruitment, examination and treatment of all participants enrolled at the Belfast site, as well as to data collection, analysis and interpretation of the trial findings. She contributed to the drafting of this paper.

For peer review only

DIAMONDS Study Group

Noemi Lois, Queen's University and Royal Victoria Hospital, Belfast H&SC Trust; Augusto Azuara-Blanco, Queen's University and Royal Victoria Hospital, Belfast H&SC Trust; Christina Campbell, NICTU, Belfast; Danny McAuley, Queen's University and Royal Victoria Hospital, Belfast H&SC Trust; Mandy Maredza, Warwick University; Hema Mistry, Warwick University; Norman Waugh, Warwick University; Nachiketa Acharya, Sheffield Teaching Hospitals NHS Foundation Trust; Tariq M Aslam, Manchester Royal Eye Hospital, Central Manchester University Hospitals NHS Foundation Trust; Clare Bailey, Bristol Eye Hospital, University Hospitals Bristol NHS Foundation Trust; Victor Chong, Royal Free Hospital NHS Foundation Trust, London; Louise Downey, Hull and East Yorkshire Hospital, Hull and East Yorkshire NHS Trust; Haralabos Eleftheriadis, Kings College Hospital NHS Foundation Trust; Samia Fatum, John Radcliffe Hospital, Oxford University Hospitals NHS Foundation Trust; Sheena George, Hillingdon Hospitals NHS Foundation Trust; Faruque Ghanchi, Bradford Teaching Hospitals NHS Trust; Markus Groppe, Stoke Mandeville Hospital, Buckinghamshire NHS Trust; Robin Hamilton, Moorfields Eye Hospital NHS Foundation Trust; Geeta Menon, Frimley Park Hospital NHS Foundation Trust; Ahmed Saad, James Cook University Hospital, South Tees Hospitals NHS Foundation Trust; Sobha Sivaprasad, Moorfields Eye Hospital NHS Foundation Trust; Marianne Shiew, Hinchingbrooke Hospital North West Anglia NHS Trust; David H Steel, Sunderland Eye Infirmary, City Hospitals Sunderland NHS Foundation Trust; James Stephen Talks, Newcastle Eye Centre, Royal Victoria Infirmary, Newcastle upon Tyne Hospitals NHS Foundation Trust; Catherine Adams, NICTU, Belfast; Paul Doherty, NICTU, Belfast; Evie Gardener, NICTU, Belfast; Aby Joseph, NICTU, Belfast; Cliona McDowell, NICTU, Belfast; Matthew Mills, NICTU, Belfast; Mike Clarke, Queens University, Belfast.

Competing interests: HM: none; **MM**: none; **CC**: none; **NL**: none.

For peer review only

Funding: DIAMONDS was funded by the Health Technology Assessment (HTA) programme of the National Institute for Health Research (NIHR), United Kingdom NIHR-HTA [13/142/04]. The NIHR had no role in the design or conduct of this research. Iridex provided a free loan for the laser equipment for the trial. Iridex had no role in the design or conduct of the trial or on data analysis or interpretation of the DIAMONDS data or writing of this report.

Data sharing statement

All data requests should be submitted to the corresponding author for consideration.

Ethics statements

Ethics Approval

The protocol for the DIAMONDS trial was approved by ORECNI (15/NI/0197). A Clinical Trial Authorisation (CTA) was obtained from the Medicines and Healthcare products Regulatory Agency (MHRA) (32485/0029/001-0001). The trial was registered with the European Union Drug Regulating Authorities Clinical Trials (EudraCT) database (2015-001940-12) and in the International Standard Randomised Controlled Trial Number (ISRCTN) register (ISRCTN16962255) and clinicaltrials.gov (NCT03690050).

Patient consent for publication

Not applicable

Figure legend/caption

Figure 1: Cost-effectiveness scatterplot with 95% confidence ellipses at 24 months for base-case within-trial analysis (NHS and PSS perspective, imputed, additionally controlled for baseline utilities)

For peer review

References

1. Yau JW, Rogers SL, Kawasaki R, Lamoureux EL, Kowalski JW, Bek T, et al. Global prevalence and major risk factors of diabetic retinopathy. Diabetes care. 2012;35(3):556-64.
2. Diabetes UK. Number of people with diabetes reaches 4.7 million London2019 [Available from: https://www.diabetes.org.uk/about_us/news/new-stats-people-living-with-diabetes.
3. Stitt AW, Curtis TM, Chen M, Medina RJ, McKay GJ, Jenkins A, et al. The progress in understanding and treatment of diabetic retinopathy. Progress in retinal and eye research. 2016;51:156-86.
4. National Institute for Health and Care Excellence. Ranibizumab for treating diabetic macular oedema (rapid review of technology appraisal guidance 237). London: NICE; 2013 April.
5. National Institute for Health and Care Excellence. Aflibercept for treating diabetic macular oedema. London: NICE; 2015 July.
6. National Institute for Health and Care Excellence. Faricimab for treating diabetic macular oedema. London: NICE; 2022 June.
7. Lois N, Campbell C, Waugh N, Azuara-Blanco A, Maredza M, Mistry H, et al. DIAbetic Macular Oedema aNd Diode Subthreshold micropulse laser (DIAMONDS): A pragmatic, double-masked, randomised, non-inferiority trial. Ophthalmology. 2023;130(1):14-27.
8. Lois N, Campbell C, Waugh N, Azuara-Blanco A, Maredza M, Mistry H, et al. DIAbetic Macular Oedema aNd Diode Subthreshold micropulse laser (DIAMONDS): A pragmatic, multicentre, allocation concealed, double-masked prospective, randomised, non-inferiority, clinical trial. Health Technology Assessment. 2022;26(50):1-86.
9. Luttrull JK, Sinclair SH. Safety of transfoveal subthreshold diode micropulse laser for fovea-involving diabetic macular edema in eyes with good visual acuity. Retina. 2014;34(10):2010-20.
10. Vujosevic S, Martini F, Longhin E, Convento E, Cavarzeran F, Midena E. Subthreshold micropulse yellow laser versus subthreshold micropulse infrared laser in center-involving diabetic macular edema: morphologic and functional safety. Retina. 2015;35(8):1594-603.
11. Wells-Gray EM, Doble N, Ohr MP, Choi SS. Structural integrity of individual cone photoreceptors after short-wavelength subthreshold micropulse laser therapy for diabetic macular edema. Ophthalmic Surgery, Lasers and Imaging Retina. 2018;49(12):946-54.
12. Husereau D, Drummond M, Augustovski F, de Bekker-Grob E, Briggs AH, Carswell C, et al. Consolidated Health Economic Evaluation Reporting Standards 2022 (CHEERS 2022) statement: updated reporting guidance for health economic evaluations. International journal of technology assessment in health care. 2022;38(1):e13.
13. Mistry H, Waugh N, Lois N. DIAMONDS Health Economics Analysis Plan v1.5 2022 [Available from: https://nictu.hscni.net/diamonds-trial-documents/.
14. Lois N, Gardner E, Waugh N, Azuara-Blanco A, Mistry H, McAuley D, et al. Diabetic macular oedema and diode subthreshold micropulse laser (DIAMONDS): study protocol for a randomised controlled trial. Trials. 2019;20(1):1-11.
15. National Institute for Health and Care Excellence. Guide to the methods of technology appraisal. London: NICE; 2013.
16. Curtis L, Burns A. Unit Costs of Health and Social Care 2020. Kent: Personal Social Services Research Unit; 2020.
17. Department of Health. NHS reference costs 2018 to 2019: GOV.UK; 2018 [Available from: https://www.england.nhs.uk/publication/2018-19-national-cost-collection-data-publication/.
18. British Medical Association and Royal Pharmaceutical Society of Great Britain. British National Formulary No. 80, September 2020-March 2021. London: BMA and PRS.
19. Van Hout B, Janssen M, Feng Y-S, Kohlmann T, Busschbach J, Golicki D, et al. Interim scoring for the EQ-5D-5L: mapping the EQ-5D-5L to EQ-5D-3L value sets. Value in Health. 2012;15(5):708-15.
20. Mangione CM, Lee PP, Gutierrez PR, Spritzer K, Berry S, Hays RD. Development of the 25-list-item national eye institute visual function questionnaire. Archives of ophthalmology. 2001;119(7):1050-8.
21. Misajon R, Hawthorne G, Richardson J, Barton J, Peacock S, Iezzi A, et al. Vision and quality of life: the development of a utility measure. Investigative ophthalmology & visual science. 2005;46(11):4007-15.
22. Peacock S, Misajon R, Iezzi A, Richardson J, Hawthorne G, Keeffe J. Vision and quality of life: development of methods for the VisQoL vision-related utility instrument. Ophthalmic epidemiology. 2008;15(4):218-23.

For peer review only

23. Little RJ, Rubin DB. Statistical analysis with missing data: John Wiley & Sons; 2019.
24. Payakachat N, Summers KH, Pleil AM, Murawski MM, Thomas J, Jennings K, et al. Predicting EQ-5D utility scores from the 25-item National Eye Institute Vision Function Questionnaire (NEI-VFQ 25) in patients with age-related macular degeneration. Quality of Life Research. 2009;18:801-13.
25. Malkin AG, Goldstein JE, Perlmutter M, Massof RW. Responsiveness of the EQ-5D to the effects of low vision rehabilitation. Optometry and vision science: official publication of the American Academy of Optometry. 2013;90(8):799.

For peer review only

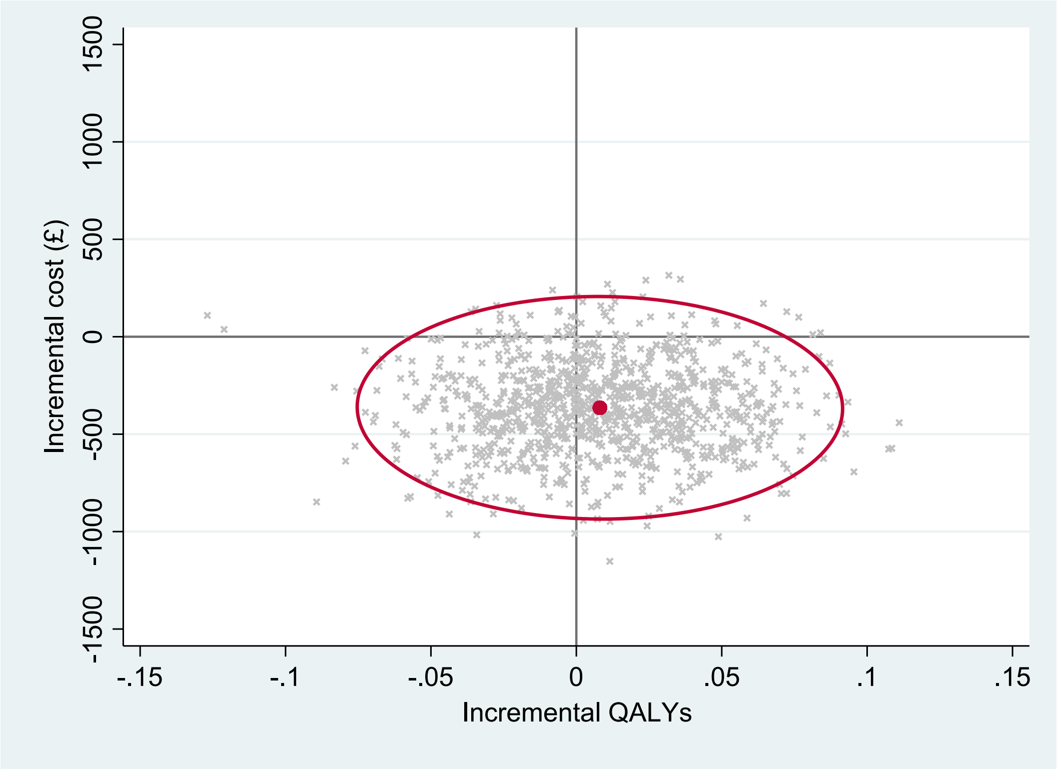

Figure 1 Cost-effectiveness scatterplot with 95% confidence ellipses at 24 months for base-case within-trial analysis (NHS and PSS perspective, imputed, additionally controlled for baseline utilities)

For peer review only - http://bmjopen.bmj.com/site/about/guidelines.xhtml

Supplemental Tables

Supplemental Table A1: Unit costs

Resource Item	Unit Cost (£, 2020)	Measurement unit	Source
**Staff Costs**			
Consultant	114	per working hour	PSSRU 2020, page 158
Associate specialist/Staff Grade	117	per working hour	PSSRU 2020, page 158
Retina Fellow	50	per working hour	PSSRU 2019, page 158
Ophthalmic photographer/ Imaging technician	52	per working hour	PSSRU 2020, page 148
**Anti-vascular endothelial growth factor costs**			
Ranibizumab	569	Per dose	NHS Reference Costs 2019-20
Aflibercept	634	Per dose	NHS Reference Costs 2019-20
Bevacizumab	277	Per dose	NHS Reference Costs 2019-20

**Laser equipment costs**				
**Laser Type**	**Current Cost[a]**	**Lifespan (years)**	**Total annual discounted costs**	**Cost per patient[b]**
Complete scanning Laser Module TxCell/Haag Streit Fit/ IQ577nm with Micropulse®[c]	£79,800 - purchase price<br>£6,990 – total cost for a 5-year preventative maintenance contract (with the first 2 years being warranty) plus VAT	14	£8,860	£2.95
Nidek GYC-1000 Laser (including installation)[d]	£14,090 - purchase price<br>£3,653 - maintenance over 5 years	7	£3,113	£1.04
Pascal Laser[e]	£51,522 - purchase price (excluding optional extras)<br>£1,548 - maintenance cost	14	£6,266	£2.09
Argon laser (new design high quality ophthalmic laser)	£14,000 – purchase price<br>£809 - maintenance costs	7	£3,010	£1.03

[a] When required, costs were inflated to 2019/20 prices using the NHS Cost Inflation Index (NHSCII). [b] Annual throughput estimate was 3,000: (Personal communication, Noemi Lois, email dated 21 April 2021).[c] Equipment price quotation: Carleton Ltd (via email on 25 July 2016). [d] Equipment price quotation: Birmingham Optical (via email on 25 July 2016). [e] Equipment price quotation: Topcon Ireland Medical (via email on 23 April 2021)
NHS: National Health Service

For peer review only

Supplemental Table A2: VisQol scores at baseline, 12 months and 24 months post-randomisation by VisQol dimension and trial group

Timepoint	VisQol Dimension	Subthreshold Micropulse laser (N = 133)		Standard threshold laser (N = 132)		Between-Group Difference (95% CI)	
		n	Unadjusted mean (SD)	n	Unadjusted mean (SD)		p-value
Baseline	Injure	130	0.953 (0.14)	116	0.967 (0.093)	-0.023 (-0.007 to 0.053)	0.141
	Cope	130	0.942 (0.109)	116	0.956 (0.119)	0.001 (-0.025 to 0.028)	0.915
	Friendships	130	0.915 (0.147)	116	0.940 (0.143)	0.003 (-0.031 to 0.037)	0.879
	Assistance	131	0.970 (0.093)	116	0.983 (0.080)	-0.001 (-0.023 to 0.02)	0.904
	Roles	131	0.927 (0.181)	116	0.961 (0.117)	0.025 (-0.007 to 0.057)	0.119
	Confidence	131	0.936 (0.158)	116	0.970 (0.075)	0.015 (-0.012 to 0.041)	0.269
12 months	Injure	130	0.975 (0.082)	109	0.950 (0.177)	-0.018 (-0.05 to 0.014)	0.272
	Cope	130	0.943 (0.105)	109	0.957 (0.121)	-0.007 (-0.036 to 0.021)	0.614
	Friendships	130	0.918 (0.141)	109	0.944 (0.143)	-0.008 (-0.045 to 0.028)	0.650
	Assistance	129	0.969 (0.117)	110	0.979 (0.086)	-0.007 (-0.030 to 0.015)	0.528
	Roles	129	0.953 (0.121)	110	0.966 (0.083)	-0.005 (-0.039 to 0.029)	0.769
	Confidence	129	0.950 (0.122)	110	0.963 (0.076)	-0.005 (-0.033 to 0.023)	0.710
24 months	Injure	113	0.985 (0.047)	112	0.955 (0.159)	0.004 (-0.029 to 0.037)	0.804
	Cope	112	0.963 (0.099)	111	0.956 (0.159)	0 (-0.030 to 0.028)	0.963
	Friendships	130	0.918 (0.127)	111	0.939 (0.136)	-0.005 (-0.042 to 0.032)	0.805
	Assistance	112	0.991 (0.043)	113	0.977 (0.138)	-0.002 (-0.0248 to 0.021)	0.880
	Roles	113	0.966 (0.113)	113	0.953 (0.138)	-0.012 (-0.047 to 0.022)	0.478
	Confidence	113	0.975 (0.046)	112	0.954 (0.121)	-0.009 (-0.037 to 0.020)	0.546

Injure: Likely to injure self; Cope: Coping with life demands; Friendships: Ability to have friendships; Assistance: Organising assistance; Roles: Difficult to fulfil roles; Confidence: Confidence to join activities.
CI: confidence interval.

For peer review only

Supplemental Table A3: NEI-VFQ-25 Subscale and Composite Scores in participants treated with micropulse subthreshold laser vs. standard threshold laser

Trial group by timepoint	Variable	Observations	Mean	Standard deviation	Minimum	Maximum
**Subthreshold Micropulse laser**						
Baseline	*NEI-VFQ-25 Composite Score*	*131*	*86.38*	*13.83*	*30.88*	*100*
	General Health	131	46.95	25.39	0.00	100
	General Vision	130	72.15	13.75	40.00	100
	Ocular Pain	131	85.02	20.65	0.00	100
	Near Activities	130	80.00	19.84	8.33	100
	Distance Activities	130	87.82	16.09	16.67	100
	Vision Social Function	130	94.62	13.56	37.50	100
	Vision Mental Health	131	82.16	19.95	18.75	100
	Vision Role Difficulties	130	83.85	23.11	0.00	100
	Vision Dependency	131	92.68	19.28	8.33	100
	Driving	92	92.84	12.08	33.33	100
	Color Vision	129	96.71	11.85	50.00	100
	Peripheral Vision	130	88.85	18.95	25.00	100
12 months	*NEI-VFQ-25 Composite Score*	*113*	*89.61*	*9.99*	*45.92*	*100*
	General Health	113	50.00	23.62	0.00	100
	General Vision	112	74.11	13.05	40.00	100
	Ocular Pain	113	89.82	17.40	12.50	100
	Near Activities	113	83.67	19.30	8.33	100
	Distance Activities	112	90.29	15.06	33.33	100
	Vision Social Function	113	96.68	8.35	50.00	100
	Vision Mental Health	113	86.06	15.38	18.75	100
	Vision Role Difficulties	112	86.27	20.95	0.00	100
	Vision Dependency	113	96.31	12.35	25.00	100
	Driving	77	94.53	9.57	50.00	100
	Color Vision	113	98.45	8.37	25.00	100
	Peripheral Vision	112	93.08	14.32	50.00	100
24 months	*NEI-VFQ-25 Composite Score*	*114*	*87.19*	*14.08*	*22.65*	*100*
	General Health	114	52.85	29.33	0.00	100
	General Vision	114	72.63	15.57	20.00	100
	Ocular Pain	114	88.38	16.61	25.00	100
	Near Activities	114	81.18	21.55	0.00	100
	Distance Activities	114	87.35	17.57	12.50	100
	Vision Social Function	113	95.24	12.87	25.00	100
	Vision Mental Health	113	83.13	20.22	0.00	100
	Vision Role Difficulties	113	85.07	24.31	0.00	100
	Vision Dependency	111	93.09	19.72	0.00	100
	Driving	81	91.82	13.57	16.67	100
	Color Vision	110	97.73	9.90	50.00	100
	Peripheral Vision	113	88.50	20.60	25.00	100
**Standard threshold laser**						

For peer review only

Baseline	*NEI-VFQ-25 Composite Score*	*130*	*87.00*	*12.73*	*44.63*	*100*
	General Health	130	51.92	25.22	0.00	100
	General Vision	130	72.92	15.52	40.00	100
	Ocular Pain	130	85.38	17.82	25.00	100
	Near Activities	130	80.16	19.33	25.00	100
	Distance Activities	130	89.01	14.17	41.67	100
	Vision Social Function	130	93.94	13.68	25.00	100
	Vision Mental Health	130	80.87	20.62	6.25	100
	Vision Role Difficulties	129	82.75	26.48	0.00	100
	Vision Dependency	130	93.72	14.57	33.33	100
	Driving	91	95.05	9.38	50.00	100
	Color Vision	130	96.35	11.69	25.00	100
	Peripheral Vision	130	91.54	16.63	25.00	100
12 months	*NEI-VFQ-25 Composite Score*	*117*	*88.47*	*13.78*	*29.21*	*100*
	General Health	116	49.78	25.00	0.00	100
	General Vision	116	75.17	14.59	40.00	100
	Ocular Pain	117	88.25	15.25	37.50	100
	Near Activities	117	83.62	19.29	8.33	100
	Distance Activities	117	88.89	17.02	25.00	100
	Vision Social Function	116	94.29	14.22	25.00	100
	Vision Mental Health	117	86.38	18.99	6.25	100
	Vision Role Difficulties	117	85.26	24.27	0.00	100
	Vision Dependency	116	92.74	19.37	0.00	100
	Driving	81	93.26	14.65	33.33	100
	Color Vision	115	98.48	6.86	50.00	100
	Peripheral Vision	115	91.96	16.07	25.00	100
24 months	*NEI-VFQ-25 Composite Score*	*115*	*88.80*	*13.78*	*29.08*	*100*
	General Health	115	51.96	24.59	0.00	100
	General Vision	114	74.39	14.94	20.00	100
	Ocular Pain	115	89.35	15.55	37.50	100
	Near Activities	115	82.79	20.03	16.67	100
	Distance Activities	115	89.53	17.41	16.67	100
	Vision Social Function	115	94.57	13.05	12.50	100
	Vision Mental Health	115	85.43	21.26	0.00	100
	Vision Role Difficulties	114	86.73	21.76	0.00	100
	Vision Dependency	114	94.01	19.03	0.00	100
	Driving	78	96.42	7.35	58.33	100
	Color Vision	115	96.52	11.89	25.00	100
	Peripheral Vision	115	91.96	16.74	25.00	00

For peer review only

Supplemental Table A4: Frequency of laser treatments and use of anti-VEGFs or steroids as rescue treatment over the 24-month follow-up period

	Subthreshold Micropulse Laser	Standard Threshold Laser	Difference (95% CI)	P-value
**Number of laser treatments used from baseline to month 24 in study eye [a b]**	2.37 (0.11) n=133	1.89 (0.11) n=132	0.48 (0.18, 0.79)	0.002
**Number of patients with at least one steroid injection in study eye (as additional treatment) from baseline to month 24[c]**	0 (0.0%)	1 (0.8%)		
**Number of patients receiving at least one anti-VEGF treatment (as additional treatment) from baseline to month 24[c]**	24 (18.1%) n=133	28 (21.2%) n=132	OR: 0.78 (0.42 – 1.45)	0.44
			% point difference: -2.8 (-13.1 – 7.5)	0.59
**Number of anti-VEGF treatments (as additional treatment) from baseline to month 24[a d]**	0.80 (0.23) n=133	1.30 (0.23) n=132	-0.50 (-1.14 – 0.14)	0.13
**Number of anti-VEGF treatments (as additional treatment) from baseline to month 24 [d e]**				
**1-2**	4 (16.7%) n=24	7 (25.0%) n=28		
**3-4**	10 (41.7%) n=24	7 (25.0%) n=28		
**5-10**	10 (41.7%) n=24	9 (32.1%) n=28		
**>10**	0 (0.0%) n=24	5 (17.9%) n=28		

[a] Mean (SE) presented for continuous outcomes [b] Number of laser treatments, were analysed using linear regression with adjustment for baseline BCVA and minimisation variables. [c] number of patients receiving at least one additional treatment (defined as at least one anti-VEGF or steroid), were analysed using logistic regression models with adjustment for the minimisation variables. [d] Number of steroid injections and number of anti-VEGF treatments (as mean and number (%) in categories), were analysed using linear regression with adjustment for minimisation variables. [e] n (%) based on no. of patients receiving anti VEGF treatments.

anti-VEGF: anti-vascular endothelial growth factor; CI: confidence interval; OR: odds ratio; RR: risk ratio

view only

CHEERS 2022 Checklist

	Item	Guidance for Reporting	Reported in section
**TITLE**			
Title	1	Identify the study as an economic evaluation and specify the interventions being compared.	1
**ABSTRACT**			
Abstract	2	Provide a structured summary that highlights context, key methods, results and alternative analyses.	4
**INTRODUCTION**			
Background and objectives	3	Give the context for the study, the study question and its practical relevance for decision making in policy or practice.	6
**METHODS**			
Health economic analysis plan	4	Indicate whether a health economic analysis plan was developed and where available.	6
Study population	5	Describe characteristics of the study population (such as age range, demographics, socioeconomic, or clinical characteristics).	6
Setting and location	6	Provide relevant contextual information that may influence findings.	6
Comparators	7	Describe the interventions or strategies being compared and why chosen.	6
Perspective	8	State the perspective(s) adopted by the study and why chosen.	6
Time horizon	9	State the time horizon for the study and why appropriate.	6
Discount rate	10	Report the discount rate(s) and reason chosen.	7
Selection of outcomes	11	Describe what outcomes were used as the measure(s) of benefit(s) and harm(s).	8-9
Measurement of outcomes	12	Describe how outcomes used to capture benefit(s) and harm(s) were measured.	8-9
Valuation of outcomes	13	Describe the population and methods used to measure and value outcomes.	8-9
Measurement and valuation of resources and costs	14	Describe how costs were valued.	8
Currency, price date, and conversion	15	Report the dates of the estimated resource quantities and unit costs, plus the currency and year of conversion.	7
Rationale and description of model	16	If modelling is used, describe in detail and why used. Report if the model is publicly available and where it can be accessed.	N/A
Analytics and assumptions	17	Describe any methods for analysing or statistically transforming data, any extrapolation methods, and approaches for validating any model used.	9
Characterizing heterogeneity	18	Describe any methods used for estimating how the results of the study vary for sub-groups.	N/A
Characterizing distributional effects	19	Describe how impacts are distributed across different individuals or adjustments made to reflect priority populations.	N/A
Characterizing uncertainty	20	Describe methods to characterize any sources of uncertainty in the analysis.	~~N/A~~
Approach to engagement with patients and others affected by the study	21	Describe any approaches to engage patients or service recipients, the general public, communities, or stakeholders (e.g., clinicians or payers) in the design of the study.	N/A
**RESULTS**			
Study parameters	22	Report all analytic inputs (e.g., values, ranges, references) including uncertainty or distributional assumptions.	1 (Suppl A1)
Summary of main results	23	Report the mean values for the main categories of costs and outcomes of interest and summarise them in the most appropriate overall measure.	10-13
Effect of uncertainty	24	Describe how uncertainty about analytic judgments, inputs, or projections affect findings. Report the effect of choice of discount rate and time horizon, if applicable.	~~N/A~~
Effect of engagement with patients and others affected by the study	25	Report on any difference patient/service recipient, general public, community, or stakeholder involvement made to the approach or findings of the study	N/A
**DISCUSSION**			
Study findings, limitations, generalizability, and current knowledge	26	Report key findings, limitations, ethical or equity considerations not captured, and how these could impact patients, policy, or practice.	13-14
**OTHER RELEVANT INFORMATION**			
Source of funding	27	Describe how the study was funded and any role of the funder in the identification, design, conduct, and reporting of the analysis	2
Conflicts of interest	28	Report authors conflicts of interest according to journal or International Committee of Medical Journal Editors requirements.	2

Husereau D, Drummond M, Augustovski F, de Bekker-Grob E, Briggs AH, Carswell C, Caulley L, Chaiyakunapruk N, Greenberg D, Loder E, Mauskopf J, Mullins CD, Petrou S, Pwu RF, Staniszewska S; CHEERS 2022 ISPOR Good Research Practices Task Force. Consolidated Health Economic Evaluation Reporting Standards 2022 (CHEERS 2022) Statement: Updated Reporting Guidance for Health Economic Evaluations. BMJ. 2022;376:e067975.
The checklist is Open Access distributed in accordance with the terms of the Creative Commons Attribution (CC BY 4.0) license, which permits others to distribute, remix, adapt and build upon this work, for commercial use, provided the original work is properly cited. See: http://creativecommons.org/licenses/by/4.0/.

For peer review only

BMJ Open

Subthreshold micropulse laser versus standard laser for the treatment of central-involving diabetic macular oedema with central retinal thickness of <400μ: a cost-effectiveness analysis from the DIAMONDS trial

Journal:	*BMJ Open*
Manuscript ID	bmjopen-2022-067684.R2
Article Type:	Original research
Date Submitted by the Author:	26-Sep-2023
Complete List of Authors:	Mistry, Hema; University of Warwick; University Hospitals Coventry and Warwickshire NHS Trust<br>Maredza, Mandy ; University of Warwick<br>Campbell, Christina; Northern Ireland Clinical Trials Unit<br>Lois, Noemi; Wellcome-Wolfson Institute for Experimental Medicine, Ophthalmology<br>Study Group, DIAMONDS; Northern Ireland Clinical Trials Unit
**Primary Subject Heading**:	Health economics
Secondary Subject Heading:	Ophthalmology
Keywords:	OPHTHALMOLOGY, Diabetic retinopathy < DIABETES & ENDOCRINOLOGY, HEALTH ECONOMICS

SCHOLARONE™
Manuscripts

I, the Submitting Author has the right to grant and does grant on behalf of all authors of the Work (as defined in the below author licence), an exclusive licence and/or a non-exclusive licence for contributions from authors who are: i) UK Crown employees; ii) where BMJ has agreed a CC-BY licence shall apply, and/or iii) in accordance with the terms applicable for US Federal Government officers or employees acting as part of their official duties; on a worldwide, perpetual, irrevocable, royalty-free basis to BMJ Publishing Group Ltd ("BMJ") its licensees and where the relevant Journal is co-owned by BMJ to the co-owners of the Journal, to publish the Work in this journal and any other BMJ products and to exploit all rights, as set out in our licence.

The Submitting Author accepts and understands that any supply made under these terms is made by BMJ to the Submitting Author unless you are acting as an employee on behalf of your employer or a postgraduate student of an affiliated institution which is paying any applicable article publishing charge ("APC") for Open Access articles. Where the Submitting Author wishes to make the Work available on an Open Access basis (and intends to pay the relevant APC), the terms of reuse of such Open Access shall be governed by a Creative Commons licence – details of these licences and which Creative Commons licence will apply to this Work are set out in our licence referred to above.

Other than as permitted in any relevant BMJ Author's Self Archiving Policies, I confirm this Work has not been accepted for publication elsewhere, is not being considered for publication elsewhere and does not duplicate material already published. I confirm all authors consent to publication of this Work and authorise the granting of this licence.

For peer review only

Subthreshold micropulse laser versus standard laser for the treatment of central-involving diabetic macular oedema with central retinal thickness of <400μ: a cost-effectiveness analysis from the DIAMONDS trial

Hema Mistry [1,5*], Mandy Maredza[2], Christina Campbell [3], Noemi Lois[4] on behalf of the DIAMONDS study group.

Affiliations

[1] Warwick Clinical Trials Unit, Warwick Medical School, University of Warwick, Coventry, CV4 7AL, UK.

[2] Division of Health Sciences, Warwick Medical School, University of Warwick, Coventry, CV4 7AL, UK.

[3] Northern Ireland Clinical Trials Unit, 7 Lennoxvale, Belfast, BT9 5BY, UK.

[4] Wellcome-Wolfson Institute for Experimental Medicine, Queen's University Belfast, 97 Lisburn Road, Belfast, BT9 7BL, UK.

[5]University Hospitals Coventry and Warwickshire NHS Trust, Clifford Bridge Road, Coventry, CV2 2DX, UK

Correspondence to: Dr Hema Mistry, Warwick Clinical Trials Unit, Warwick Medical School, University of Warwick, Coventry, UK; Email: Hema.Mistry@warwick.ac.uk; Tel: +44 (0)2476 151183

Key words:

Ophthalmology, Diabetic retinopathy, Diabetes & Endocrinology, Health Economics

For peer review only

Abstract (currently 297 words, 300 words max)

Objectives

To estimate the economic costs, health-related quality of life outcomes and cost-effectiveness of subthreshold micropulse laser (SML) versus standard laser (SL) for the treatment of diabetic macular oedema (DMO) with central retinal thickness (CRT) of <400μ.

Design

An economic evaluation was conducted within a pragmatic, multicentre, randomised clinical trial, DIAMONDS.

Setting

18 UK Hospital Eye Services.

Participants

Adults with diabetes and centre involving DMO with CRT <400μ.

Interventions

Participants (n=266) were randomised 1:1 to receive SML or SL.

Methods

The base-case used an intention-to-treat approach conducted from a UK National Health Service (NHS) and personal social services (PSS) perspective. Costs (2019–2020 prices) were collected prospectively over the 2-year follow-up period. A bivariate regression of costs and quality-adjusted life-years (QALYs), with multiple imputation of missing data, was conducted to estimate the incremental cost per QALY gained and the incremental net monetary benefit of SML in comparison to SL. Sensitivity analyses explored uncertainty and heterogeneity in cost-effectiveness estimates.

Results

One participant in the SL arm withdrew consent for data to be used; data from the remaining 265 participants were included in analyses. Mean (standard error [SE]) NHS and PSS costs over 24 months were £735.09 [£111.85] in the SML arm versus £1099.70 [£195.40] in the SL arm (p=0.107). Mean (SE) QALY estimates were 1.493 [0.024] versus 1.485 [0.020], respectively (p=0.780), giving an

For peer review only

insignificant difference of 0.008 QALYs. The probability SML is cost-effective at a threshold of £20,000 per QALY was 76%.

Conclusions

There were no statistically significant differences in EQ-5D-5L scores or costs between SML and SL. Given these findings and the fact that SML does not burn the retina, unlike SL, and has equivalent efficacy to SL, it may be preferred for the treatment of people with DMO with CRT <400μ.

Trial registration number

ISRCTN17742985; NCT03690050

For peer review only

Strengths and limitations of this study

- Study is based on a high-quality randomised clinical trial.
- First study to compare the cost-effectiveness of subthreshold micropulse laser (SML) with that of standard laser (SL) for the treatment of diabetic macular oedema (DMO).
- The analysis was from a UK National Health Service (NHS) and Personal Social Services (PSS) perspective and did not consider costs such as productivity losses from a societal perspective.
- Low rate of missing data for both costs and outcomes.
- We used published costs of anti-VEGF drugs, rather than NHS costs which incorporates confidential price discounts for these drugs.

For peer review only

Manuscript word count 3309 words (including the 3 tables); max 4000 words; up to 5 images/tables

Introduction

Diabetic macular oedema (DMO) is a visual threatening complication of diabetes, occurring in approximately 7% of people living with diabetes.(1) Given the high and continuously increasing prevalence of diabetes,(2) DMO is a frequent eye disease requiring treatment in ophthalmic clinics in the UK and worldwide. DMO can impose a significant social and economic burden on society, due to its high prevalence and associated costs. Very few studies have explored the economic burden of DMO and even fewer have reported on its cost-effectiveness. A cost-of-illness study using cohort data from US Medicare data reported just under 38% with DMO underwent laser photocoagulation and their 1-year mean direct medical costs amounted to US$11,290, 31% higher than for those without DMO.(3) In a cohort study from South Korea, the mean 1-year medical costs were higher for people with DMO (US$6,723) than those who had diabetes without retinopathy.(4) The estimated health care and social care costs for DMO in England in 2010 were £92 million and £11.6 million respectively, with £65.6 million of this being spent on hospital treatment and related costs.(5)

In DMO fluid accumulates in the center of the retina, the macula, which is the area of the retina responsible for providing central vision.(6) The purpose of the treatment is to restore the anatomy of the macula by clearing up this fluid and restoring vision. Treatments include intraocular injections of anti-vascular endothelial growth factor (anti-VEGF) drugs or steroids and macular laser. The National Institute for Health and Care Excellence (NICE) recommends anti-VEGF therapy for people with more severe forms of DMO, with central retinal (macular) thickness (CRT) of 400µ or above, as measured in scans obtained using an imaging modality called optical coherence tomography (OCT).(7-9) For milder forms of DMO (CRT <400µ) NICE recommends macular laser. Intravitreal steroids are advised for people that do not respond to the above-mentioned therapies.

For peer review only - http://bmjopen.bmj.com/site/about/guidelines.xhtml

DIAbetic Macular Oedema aNd Diode Subthreshold micropulse laser (DIAMONDS) was a pragmatic, allocation-concealed, double-masked, multicentre, randomised, non-inferiority clinical trial which compared the clinical effectiveness and cost-effectiveness of subthreshold micropulse laser (SML) and standard laser (SL) for the treatment of people with DMO with CRT of <400µ. DIAMONDS participants were randomised 1:1 to receive SML (577 nm) or SL (e.g. using argon, frequency-doubled neodymium-doped yttrium aluminium garnet [Nd:YAG] 532 nm laser). Laser treatment could be repeated as needed, using the allocated laser at randomisation and rescue treatment with anti-VEGFs or steroids was allowed. The primary outcome was the mean change in best-corrected visual acuity (BCVA) in the study eye at 24 months. DIAMONDS found SML and SL to have equivalent clinical efficacy.(10, 11) This finding is clinically important given the fact that SML, unlike SL, does not cause any functional or structural damage to the retina(12-14) and, thus, may be preferred by patients and doctors. Herein, we present the detailed within-trial economic evaluation comparing costs and benefits of the two laser modalities, SML and SL. To our knowledge, no other trials have compared the cost-effectiveness of SML and SL for the treatment of DMO before.

Methods

Patient and Public Involvement

At the very early stages of the DIAMONDS trial conception, a DIAMONDS Patient and Public Involvement (PPI) group was established with the help of the Northern Ireland branch of DIABETES UK. The DIAMONDS PPI group comprised people living with diabetes and DMO, including a large group of members of the “Diabetes Family” Facebook group. The DIAMONDS PPI group contributed to the trial design and the research question, including the selection of outcomes, preparation of patient related materials for the trial, recruitment strategies, interpretation of trial results, and preparation of the plain English summary. They also have a major role in the dissemination and implementation of trial results.

For peer review only - http://bmjopen.bmj.com/site/about/guidelines.xhtml

The study is reported as per Consolidated Health Economic Evaluation Reporting Standards (CHEERS) 2022 Statement.(15) As detailed in the Health Economics Analysis Plan (HEAP)(16) we planned to conduct a within-trial analysis comparing the cost-effectiveness of SML with SL. The protocol for the DIAMONDS trial envisaged that economic modelling might be required if visual outcomes differed between arms.(17) The DIAMONDS protocol was designed to minimise visual loss in participants and, thus, repeating laser treatment or undertaking rescue treatment with intravitreal anti-VEGF drugs and/or steroids if criteria for rescue were met, were allowed in either arm of the trial. DIAMONDS recruited 266 participants, 116 (87%) in the SML arm and 115 (86%) in the SL arm had primary outcome data, fulfilling the requirements of the power calculation (113 participants with BCVA data at month 24 were required). DIAMONDS found SML and SL to be equivalent in terms of the primary outcome.(10, 11) Hence, economic modelling was not required. Herein, we report detailed economic costs, health-related quality of life (HRQoL) outcomes and cost-effectiveness of SML versus SL.

The economic evaluation took the form of a cost-utility analysis, expressed in terms of cost per quality-adjusted life-year (QALY) gained. The study was conducted over the 24-month time horizon of the trial. The primary analysis adopted a National Health Service (NHS) and personal and social service (PSS) perspective. Costs and outcomes in the second year of follow-up were discounted at 3.5% in line with the NICE reference case.(18)

Resource use data were collected and reported on trial case record forms (CRFs) at scheduled clinic visits at 4, 8, 12, 16, 20 and 24 months. Data were collected on the costs of laser treatment, both at the initial laser session and at subsequent ones if required, outpatient visits, and intravitreal anti-VEGF and/or steroid treatment (costs of drugs and administration) if rescue was required. All costs were expressed in Great British pounds sterling and valued in 2019–20 prices. If costs were not in line, they were inflated to 2019-20 prices using the NHS Cost Inflation Index (NHSCII).(19)

For peer review only

The costs of laser treatment included staff and equipment costs (capital and maintenance costs of laser machines). Unit costs for staff were obtained from the Unit Costs of Health and Social Care 2019 compendium (19) and were based on the time it took to for each procedure to be undertaken. These times were recorded on CRFs and included: (i) the time taken to obtain fundus fluorescein angiography (FFA) and spectral domain optical coherence tomography (SD-OCTs) scans to guide laser treatment (if used) and (ii) time taken by ophthalmologists to perform the laser procedure, including the time for counselling the participant. Costs of laser machines were obtained from manufacturers. An annual equivalent cost of equipment was obtained by annuitising the capital costs of the item over its useful life span and applying a discount rate of 3.5% per annum. A per-patient cost of equipment was estimated by assuming that the laser machines were used to treat 3,000 patients per year.

Data were also collected on any outpatient attendances or hospital admissions related to DMO or the treatments. Where an outpatient attendance was reported but no procedure undertaken, the average unit cost of an outpatient ophthalmology visit was used (varying between £80-£101 per consultation depending on whether the consultation was 'non-consultant' versus 'consultant-led').(20) The CRFs recorded data on the grade of professional that attended the patient, for example, if a consultant attended to the patient, then the consultant-led unit cost was applied. Where a procedure(s) was undertaken as part of the visit the relevant HRG code was derived using the HRG4+ Reference Costs Grouper Software (NHS Digital, Leeds, UK).

CRFs also recorded information on other tests or investigations, medication use including anti-VEGF/steroids or other rescue treatments. Anti-VEGF and steroid drugs were separately costed as these are considered an unbundled HRG. Costing of laser retreatments followed the same approach as costing for the index (first session) laser procedure.

For peer review only

Unit costs were derived from national compendia in accordance with NICE's Guide to the Methods of Technology Appraisal.(18) The key databases included the Department of Health and Social Care's Reference Costs 2018–19 schedules,(20) the PSSRU's Unit Costs of Health and Social Care 2020 compendium (19), 2020 volume of the British National Formulary.(21) Supplemental Table A1 gives a summary of the unit costs for resource use and the laser equipment. Resource inputs were valued by attaching unit costs.

The HRQoL of trial participants was assessed at baseline and at 12- and 24-months post-randomisation using the EQ-5D-5L instrument. The EQ-5D-5L questionnaire was used to generate QALYs for the cost-effectiveness analysis. (22) The QALY is a measure that combines quantity and quality of life lived into a single metric, with one QALY equating to one year of full health. To convert EQ-5D-5L responses into health utility scores we used the EQ-5D-5L Crosswalk Index Value Calculator which maps the EQ-5D-5L descriptive system data onto the EQ-5D-3L valuation set. This valuation set was recommended by NICE at the time when the analysis was undertaken.(22)

HRQoL was also assessed using two vision specific measures: the National Eye Institute Visual Functioning Questionnaire - 25 (NEI-VFQ-25) and Vision and Quality of Life Index (VisQoL).(23-25) The NEI-VFQ-25 is a validated questionnaire that has been used widely to evaluate visual outcomes in patients with eye diseases including diabetic retinopathy and DMO. The VisQol questionnaire has not been widely validated but is shorter than the NEI-VFQ-25 with only six attributes: physical well-being, independence, social well-being, self-actualisation, planning and organisation. The utilities for VisQoL were developed using a time-trade off exercise in people who were visually impaired which included patients with age-related macular degeneration, diabetic retinopathy and glaucoma.(25)

For peer review only

Summary statistics were generated for resource use variables, health utility values and QALYs by treatment allocation and assessment time point. For resource use and costs, mean values were compared between groups using two sample t-tests. Differences between groups, along with 95% confidence intervals (CIs) were estimated using non-parametric bootstrap estimates (10,000 replications). For HRQoL, these were presented as mean values with their associated standard errors. Between-group differences were compared using the two-sample t-test.

Multiple imputation by chained equations was used to predict missing health status (utility) scores and costs based on the assumption that data were missing at random (MAR). Twenty imputed data sets were generated and used to inform the base-case analyses. Parameter estimates were pooled across the 20 imputed data sets using Rubin's rules to account for between- and within-imputation components of variance terms associated with parameter estimates.(26)

The base-case cost-effectiveness analysis was performed using an intention-to-treat approach. Mean incremental costs and QALYs were estimated using seemingly unrelated regression (SUR) methods that account for the correlation between costs and outcomes. The SUR adjusted for covariates (baseline utilities, baseline body mass index (BMI), baseline best corrected distance visual acuity (BCVA), patient-reported previous use of anti-VEGF at baseline and previous use of macular laser).

Results

As reported elsewhere (10, 11) there was no difference in the clinical effectiveness of SML and SL. Table 1 shows that there were also no statistically significant differences between laser groups in EQ-5D-5L scores at baseline, 12 and 24 months.

For peer review only

Table 1: EQ-5D-5L utility scores at baseline, 12 and 24 months and QALYs (base-case, imputed analysis)

Variable	Subthreshold micropulse laser (n = 133)	Standard laser (n = 132)	Between-Group Difference (95% CI)	p-value
	Mean (SE)	Mean (SE)		
**EQ-5D Utility Scores***				
Baseline	0.758 (0.267)	0.772 (0.226)	-0.014 (-0.074 to 0.046)	0.640
12 months	0.767 (0.250)	0.758 (0.017)	0.009 (-0.041to 0.059)	0.717
24 months	0.739 (0.278)	0.743 (0.279)	-0.004 (-0.064 to 0.056)	0.897
**EQ-5D-5L QALYs**				
Over 2 years	1.493 (0.024)	1.485 (0.024)	0.008 (-0.061 to 0.075)	0.836

*Analysis adjusted for participant age, gender, baseline BCVA (Best Corrected Distance Visual Acuity) and participant's previous use of Anti-VEGF (Anti-Vascular Endothelial Growth Factor) and laser therapy at baseline, with repeated measures within participant and site.

SE = standard error; CI = confidence interval

Furthermore, there were no statistically significant differences between the two laser treatment groups for any of the VisQoL dimensions or NEI-VFQ-25 subscales at any follow-up time point (Supplemental Table A2 and Supplemental Table A3, respectively).

The mean numbers of laser treatments performed were 2.4 in the SML and 1.9 in the SL arm. This difference was statistically significant (p=0.002) (Supplemental Table A4), but equated to less than one further session of laser in the SML arm when compared with the SL arm during the 2-year trial. Furthermore, this difference was driven by a small number of participants requiring a higher number of lasers sessions in the SML group (13 participants required 6 or 7 laser treatments in the SML arm compared to 2 in the SL arm). Eighteen percent of participants in the SML arm and 21% in the SL arm required rescue treatments in the study eye (almost all with anti-VEGF drugs; in addition, only one participant had a steroid injection). The average numbers of anti-VEGF treatments per arm from

For peer review only

baseline to 24 months were 1.06 in the SML arm and 1.96 in the SL (Supplemental Table A4). The number in the SL arm was skewed by five participants who received 10 or more injections.

Table 2 summarises the NHS costs associated with resource use in the base-case (imputed) analysis by cost category and follow-up period. Costs of the first laser procedure are reported separately from those of subsequent laser retreatments. The total costs of laser therapy for each participant includes costs of the first laser procedure plus any subsequent laser retreatments they had. The mean (standard error (SE)) cost for the first laser procedure (including costs of performing OCT and FFA to guide laser treatment, if done) was £45.59 (1.64) for the SML compared with £42.29 (1.69) for the SL; the difference was not statistically significant(p=0.09). The mean total NHS and PSS costs were lower in SML compared with the SL (£735.09 vs. £1099.70) between baseline to 24 months post-randomisation; this difference was not statistically significant at the 5% level. The CI around total costs were wide and overlapped and the difference was driven by the higher number of anti-VEGF rescue injections.

Table 2: *Economic costs by trial allocation arm and cost component category for the entire follow-up period in base-case (imputed) analysis (£, 2019-20 prices)*

**Parameter**	**Subthreshold Micropulse laser Mean Costs (SE)**	**Standard threshold laser Mean Costs (SE)**	**Mean Difference**	**Bootstrap 95% CI**
Index Laser Procedure	45.59 (1.64)	42.29 (1.69)	3.31	(-1.33 to 7.95)
Laser retreatments	53.02 (5.17)	41.69 (4.42)	11.32	(-2.08 to 24.73)
Outpatient care	124.85 (22.56)	130.32 (31.58)	-5.47	(-81.92 to 70.97)
Anti-VEGF drug costs	511.63 (105.85)	885.40 (183.29)	-373.77	(-791.01 to 43.48)
**Total NHS and PSS Costs**	**735.09 (111.85)**	**1,099.70 (195.40)**	**-364.61**	**(-807.09 to 77.87)**
SE = standard error; CI = confidence interval				

Over the 2-year follow-up period, participants in the SML arm, compared to the SL arm, experienced a non-statistically significant increase in QALYs of 0.008 (circa 3 days of good quality of life). In addition, the mean NHS and PSS costs were lower in the SML arm compared to the SL arm (mean cost difference –£365) – see Table 3. The confidence interval for the cost difference was wide and ranged from cost saving to cost increasing. Although neither costs nor benefits were statistically significantly different between SML and SL, the incremental cost-effectiveness ratio (ICER) for the base-case imputed analysis indicated that SML dominates, as average costs for this intervention were slightly lower and average benefits marginally higher (but not significantly so) than those for SL. Assuming cost-effectiveness threshold of £20,000 per QALY, the probability that SML was cost-effective was 0.76, and the NMB associated with SML was positive. Figure 1 shows the joint distributions of costs and outcomes.

Table 3: Cost-effectiveness, cost/QALY (£, 2020): SML compared to SL

Mean incremental cost (95% CI)	Mean incremental QALY (95% CI)	ICER	Probability of cost-effectiveness*	Net Monetary Benefit (NMB)*
Base-case analysis - ITT approach: Imputed attributable costs and QALYs, covariate adjusted[a]				
-365 (-822 to 93)	0.008 (-0.059 to 0.075)	Dominant	0.763	520 (-925 to 1,965)

***Cost-effectiveness threshold is at £20,000/QALY threshold**

[a] Adjusted for baseline EQ-5D utility, BMI and minimisation variables at baseline (best corrected distance visual acuity and previous use of laser treatment)

CI = confidence interval; ICER = incremental cost-effectiveness ratio; ITT = intention-to-treat approach; QALY = quality-adjusted life year

Discussion

DIAMONDS was a pragmatic clinical trial carried out in 16 NHS ophthalmology departments throughout the UK. The DIAMONDS trial was powered to detect not only differences in the primary outcome (BCVA) but also in important secondary outcomes (CRT and vision quality of life).(11) However, a limitation of this cost-effectiveness analysis is that we did not use BCVA as the primary outcome, instead we used QALYs as the main outcome. Participants were treated and followed as per routine clinical care. Costs and outcome data were collected prospectively. We found no

For peer review only

significant differences in EQ-5D-5L and only a trivial non-significant difference of 0.008 in a calculation of QALYs. So, the verdict on whether one form of laser is better than the other shifts the focus onto the costs. This analysis found that costs were slightly higher (but not statistically significantly so) in the SL arm compared to the SML arm, due to more participants in the SL arm needing higher numbers (10 or more) of anti-VEGF rescue injections. Reporting both the costs and QALYs together in a ratio form, this meant the ICER for the base-case analysis indicated that SML is the dominant procedure, as average costs for this intervention were lower and average benefits were marginally higher than those for SL. However, caution should be taken when interpreting the results, given the wide confidence intervals around the mean costs (which ranges from cost saving to cost increasing). Taking this into consideration, costs of SML and SL treatment arms seem comparable. We also conducted a per-protocol analysis as part of a sensitivity analysis as DIAMONDS was a non-inferiority trial. The results were in line with the intention-to-treat analysis, were SML remained the dominant option and the probability of of SML being cost-effectiveness at the £20k/QALY threshold was 0.773.

As noted in the main clinical effectiveness manuscript(10) there may be advantages of SML over SL. , Among these and importantly, the fact that SML does not cause any functional or structural damage to the retina(12-14) and, thus, can be repeated as needed. This is an advantage for patients as their retina will remain intact despite the application of SML but would lose cells as a result of the laser burn if SL is applied. Furthermore, the lack of a burn following SML makes the delivery of the treatment safer in less experienced hands as, unlike SL, it does not carry a risk of burning the fovea. Furthermore, for the same reason, it may allow training of allied non-medical staff to undertake this procedure, who currently, are already undertaking anti-VEGF injections in the UK and other parts of the world. This would help alleviate clinical capacity constraints experienced by most ophthalmology units across the world. The clinical effectiveness results reported previously(10, 11)

For peer review only

and the HRQoL results presented here showed that SML is as clinically effective as SL at a similar cost.

In DIAMONDS, two vision-specific patient reported outcome quality of life instruments were used in addition to the generic preference-based EQ-5D-5L. Neither showed any statistically significant differences between laser arms. It has sometimes been mentioned that changes in vision that are sufficient to affect some activities of daily living, may not be reflected in changes in EQ-5D, because the EQ-5D may not be as sensitive to detect changes in quality of life as other visual specific questionnaires such as the NEI-VFQ-25.(7, 27, 28) A mapping exercise from DIAMONDS data is underway to explore this important aspect.

One limitation in the current analysis is the fact that we had to use published costs of anti-VEGF drugs (at list prices, these can range from £5 to £7k a year),(7-9) rather than costs to the NHS. We know that there are confidential price discounts for these drugs when used in the NHS (without taking into account the administration of the injection into the eye and monitoring visits) and this will most likely reduce the cost differential between the two arms.(7-9) Another limitation is that DIAMONDS included only patients with CRT <400µ, so extrapolations with regard to the cost-effectiveness of SML when compared with SL in thicker retinas, where standard laser is known to be less effective, cannot be made. Furthermore, the analysis was conducted from a NHS and PSS perspective, so we have not taken into account any broader societal costs such as time off work or care for children or other dependents, when patients have laser treatment.

Both SML and SL were successful in 80% of participants.(10) Macular laser treatment is known to be less expensive than anti-VEGF drugs and may be more convenient and acceptable to participants, although no trials have been conducted comparing the cost-effectiveness of macular laser with anti-

For peer review only

VEGFs in people with <400μ CRT DMO. Thus, SML should be considered as first line therapy for patients with central involving DMO with CRT of <400μ.

Contributor statement/Acknowledgements

The Authors would like to thank all the DIAMONDS participants and the DIAMONDS patient and public involvement (PPI) group. We also thank Dr Pamela Royle for literature searches. We would also like to thank Iridex for providing a free loan for the laser equipment for the trial and William Moore, Joan Stauffer, George Marcellino (*in memoriam*), Gareth Hymas, Nick Fitrzyk, and Suzanne Kelly, for their support. Iridex had no role in the design, data analysis or interpretation of the DIAMONDS data or in the writing of this report. We thank also the NIHR HTA programme for providing funding for the trial and note that the views expressed in this paper are those of the authors and not necessarily those of the NHS, the NIHR or the Department of Health and Social Care.

For peer review only

Author contributions

Hema Mistry contributed to the design of the health economics plan and provided oversight of all aspects of the economic evaluation including its design, conduct, analysis and reporting of this paper.

Mandy Maredza (University of Warwick) conducted the analysis and reporting of the economic evaluation and contributed to the drafting of this paper.

Christina Campbell (Trial Statistician, NICTU) performed the statistical analyses, assisted with interpretation of data and contributed to the drafting of this paper.

Professor Noemi Lois (Clinical Professor of Ophthalmology, Wellcome-Wolfson Institute for Experimental Medicine, Queen's University Belfast) is the Chief Investigator for the DIAMONDS trial conceived the trial, with input from the DIAMONDS Study Group, and led it to its successful completion. In addition, Prof Lois contributed with the identification, recruitment,

examination and treatment of all participants enrolled at the Belfast site, as well as to data collection, analysis and interpretation of the trial findings. She contributed to the drafting of this paper.

DIAMONDS Study Group

Noemi Lois, Queen's University and Royal Victoria Hospital, Belfast H&SC Trust; Augusto Azuara-Blanco, Queen's University and Royal Victoria Hospital, Belfast H&SC Trust; Christina Campbell, NICTU, Belfast; Danny McAuley, Queen's University and Royal Victoria Hospital, Belfast H&SC Trust; Mandy Maredza, Warwick University; Hema Mistry, Warwick University; Norman Waugh, Warwick University; Nachiketa Acharya, Sheffield Teaching Hospitals NHS Foundation Trust; Tariq M Aslam, Manchester Royal Eye Hospital, Central Manchester University Hospitals NHS Foundation Trust; Clare Bailey, Bristol Eye Hospital, University Hospitals Bristol NHS Foundation Trust; Victor Chong, Royal Free Hospital NHS Foundation Trust, London; Louise Downey, Hull and East Yorkshire Hospital, Hull and East Yorkshire NHS Trust; Haralabos Eleftheriadis, Kings College Hospital NHS Foundation Trust; Samia Fatum, John Radcliffe Hospital, Oxford University Hospitals NHS Foundation Trust; Sheena George, Hillingdon Hospitals NHS Foundation Trust; Faruque Ghanchi, Bradford Teaching Hospitals NHS Trust; Markus Groppe, Stoke Mandeville Hospital, Buckinghamshire NHS Trust; Robin Hamilton, Moorfields Eye Hospital NHS Foundation Trust; Geeta Menon, Frimley Park Hospital NHS Foundation Trust; Ahmed Saad, James Cook University Hospital, South Tees Hospitals NHS Foundation Trust; Sobha Sivaprasad, Moorfields Eye Hospital NHS Foundation Trust; Marianne Shiew, Hinchingbrooke Hospital North West Anglia NHS Trust; David H Steel, Sunderland Eye Infirmary, City Hospitals Sunderland NHS Foundation Trust; James Stephen Talks, Newcastle Eye Centre, Royal Victoria Infirmary, Newcastle upon Tyne Hospitals NHS Foundation Trust; Catherine Adams, NICTU, Belfast; Paul Doherty, NICTU, Belfast; Evie Gardener, NICTU, Belfast; Aby Joseph, NICTU, Belfast; Cliona McDowell, NICTU, Belfast; Matthew Mills, NICTU, Belfast; Mike Clarke, Queens University, Belfast.

For peer review only

Competing interests: HM: none; **MM**: none; **CC**: none; **NL**: none.

Funding: DIAMONDS was funded by the Health Technology Assessment (HTA) programme of the National Institute for Health Research (NIHR), United Kingdom NIHR-HTA [13/142/04]. The NIHR had no role in the design or conduct of this research. Iridex provided a free loan for the laser equipment for the trial. Iridex had no role in the design or conduct of the trial or on data analysis or interpretation of the DIAMONDS data or writing of this report.

Data sharing statement

All data requests should be submitted to the corresponding author for consideration.

Ethics statements

Ethics Approval

The protocol for the DIAMONDS trial was approved by ORECNI (15/NI/0197). A Clinical Trial Authorisation (CTA) was obtained from the Medicines and Healthcare products Regulatory Agency (MHRA) (32485/0029/001-0001). The trial was registered with the European Union Drug Regulating Authorities Clinical Trials (EudraCT) database (2015-001940-12) and in the International Standard Randomised Controlled Trial Number (ISRCTN) register (ISRCTN16962255) and clinicaltrials.gov (NCT03690050).

Patient consent for publication

Not applicable

Figure legend/caption

Figure 1: Cost-effectiveness scatterplot with 95% confidence ellipses at 24 months for base-case within-trial analysis (NHS and PSS perspective, imputed, additionally controlled for baseline utilities)

For peer review only

For peer review only

References

1. Yau JW, Rogers SL, Kawasaki R, Lamoureux EL, Kowalski JW, Bek T, et al. Global prevalence and major risk factors of diabetic retinopathy. Diabetes care. 2012;35(3):556-64.
2. Diabetes UK. Number of people with diabetes reaches 4.7 million London2019 [Available from: https://www.diabetes.org.uk/about_us/news/new-stats-people-living-with-diabetes.
3. Shea AM, Curtis LH, Hammill BG, Kowalski JW, Ravelo A, Lee PP, et al. Resource use and costs associated with diabetic macular edema in elderly persons. Archives of ophthalmology. 2008;126(12):1748-54.
4. Jeon HL, Lee H, Yoon D, Lee Y, Kim JH, Jee D, et al. Burden of diabetic macular oedema in patients receiving antivascular endothelial growth factor therapy in South Korea: a healthcare resource use and cost analysis. BMJ Open. 2020;10(12):e042484.
5. Minassian D, Owens DR, Reidy A. Prevalence of diabetic macular oedema and related health and social care resource use in England. British journal of ophthalmology. 2012;96(3):345-9.
6. Stitt AW, Curtis TM, Chen M, Medina RJ, McKay GJ, Jenkins A, et al. The progress in understanding and treatment of diabetic retinopathy. Progress in retinal and eye research. 2016;51:156-86.
7. National Institute for Health and Care Excellence. Ranibizumab for treating diabetic macular oedema (rapid review of technology appraisal guidance 237). London: NICE; 2013 April.
8. National Institute for Health and Care Excellence. Aflibercept for treating diabetic macular oedema. London: NICE; 2015 July.
9. National Institute for Health and Care Excellence. Faricimab for treating diabetic macular oedema. London: NICE; 2022 June.
10. Lois N, Campbell C, Waugh N, Azuara-Blanco A, Maredza M, Mistry H, et al. DIAbetic Macular Oedema aNd Diode Subthreshold micropulse laser (DIAMONDS): A pragmatic, double-masked, randomised, non-inferiority trial. Ophthalmology. 2023;130(1):14-27.
11. Lois N, Campbell C, Waugh N, Azuara-Blanco A, Maredza M, Mistry H, et al. DIAbetic Macular Oedema aNd Diode Subthreshold micropulse laser (DIAMONDS): A pragmatic, multicentre, allocation concealed, double-masked prospective, randomised, non-inferiority, clinical trial. Health Technology Assessment. 2022;26(50):1-86.
12. Luttrull JK, Sinclair SH. Safety of transfoveal subthreshold diode micropulse laser for fovea-involving diabetic macular edema in eyes with good visual acuity. Retina. 2014;34(10):2010-20.
13. Vujosevic S, Martini F, Longhin E, Convento E, Cavarzeran F, Midena E. Subthreshold micropulse yellow laser versus subthreshold micropulse infrared laser in center-involving diabetic macular edema: morphologic and functional safety. Retina. 2015;35(8):1594-603.
14. Wells-Gray EM, Doble N, Ohr MP, Choi SS. Structural integrity of individual cone photoreceptors after short-wavelength subthreshold micropulse laser therapy for diabetic macular edema. Ophthalmic Surgery, Lasers and Imaging Retina. 2018;49(12):946-54.
15. Husereau D, Drummond M, Augustovski F, de Bekker-Grob E, Briggs AH, Carswell C, et al. Consolidated Health Economic Evaluation Reporting Standards 2022 (CHEERS 2022) statement: updated reporting guidance for health economic evaluations. International journal of technology assessment in health care. 2022;38(1):e13.
16. Mistry H, Waugh N, Lois N. DIAMONDS Health Economics Analysis Plan v1.5 2022 [Available from: https://nictu.hscni.net/diamonds-trial-documents/.
17. Lois N, Gardner E, Waugh N, Azuara-Blanco A, Mistry H, McAuley D, et al. Diabetic macular oedema and diode subthreshold micropulse laser (DIAMONDS): study protocol for a randomised controlled trial. Trials. 2019;20(1):1-11.
18. National Institute for Health and Care Excellence. Guide to the methods of technology appraisal. London: NICE; 2013.
19. Curtis L, Burns A. Unit Costs of Health and Social Care 2020. Kent: Personal Social Services Research Unit; 2020.
20. Department of Health. NHS reference costs 2018 to 2019: GOV.UK; 2018 [Available from: https://www.england.nhs.uk/publication/2018-19-national-cost-collection-data-publication/.
21. British Medical Association and Royal Pharmaceutical Society of Great Britain. British National Formulary No. 80, September 2020-March 2021. London: BMA and PRS.
22. Van Hout B, Janssen M, Feng Y-S, Kohlmann T, Busschbach J, Golicki D, et al. Interim scoring for the EQ-5D-5L: mapping the EQ-5D-5L to EQ-5D-3L value sets. Value in Health. 2012;15(5):708-15.

23. Mangione CM, Lee PP, Gutierrez PR, Spritzer K, Berry S, Hays RD. Development of the 25-list-item national eye institute visual function questionnaire. Archives of ophthalmology. 2001;119(7):1050-8.
24. Misajon R, Hawthorne G, Richardson J, Barton J, Peacock S, Iezzi A, et al. Vision and quality of life: the development of a utility measure. Investigative ophthalmology & visual science. 2005;46(11):4007-15.
25. Peacock S, Misajon R, Iezzi A, Richardson J, Hawthorne G, Keeffe J. Vision and quality of life: development of methods for the VisQoL vision-related utility instrument. Ophthalmic epidemiology. 2008;15(4):218-23.
26. Little RJ, Rubin DB. Statistical analysis with missing data: John Wiley & Sons; 2019.
27. Payakachat N, Summers KH, Pleil AM, Murawski MM, Thomas J, Jennings K, et al. Predicting EQ-5D utility scores from the 25-item National Eye Institute Vision Function Questionnaire (NEI-VFQ 25) in patients with age-related macular degeneration. Quality of Life Research. 2009;18:801-13.
28. Malkin AG, Goldstein JE, Perlmutter M, Massof RW. Responsiveness of the EQ-5D to the effects of low vision rehabilitation. Optometry and vision science: official publication of the American Academy of Optometry. 2013;90(8):799.

For peer review only

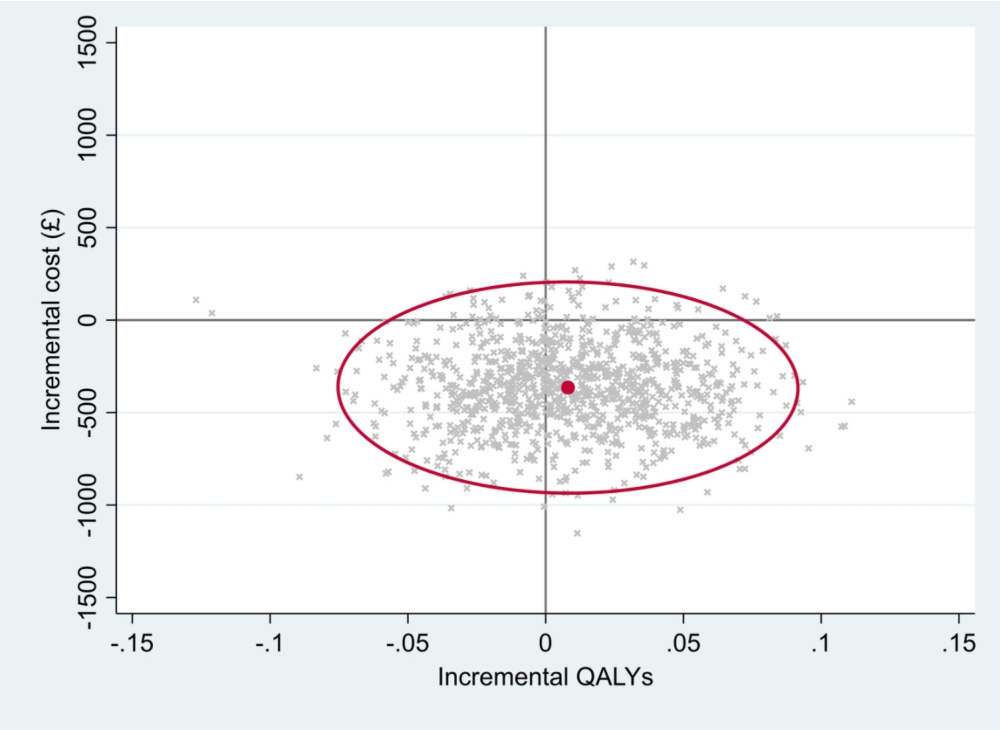

139x101mm (300 x 300 DPI)

Supplemental Tables

Supplemental Table A1: Unit costs

Resource Item	Unit Cost (£, 2020)	Measurement unit	Source
**Staff Costs**			
Consultant	114	per working hour	PSSRU 2020, page 158
Associate specialist/Staff Grade	117	per working hour	PSSRU 2020, page 158
Retina Fellow	50	per working hour	PSSRU 2019, page 158
Ophthalmic photographer/ Imaging technician	52	per working hour	PSSRU 2020, page 148
**Anti-vascular endothelial growth factor costs**			
Ranibizumab	569	Per dose	NHS Reference Costs 2019-20
Aflibercept	634	Per dose	NHS Reference Costs 2019-20
Bevacizumab	277	Per dose	NHS Reference Costs 2019-20

Laser equipment costs

Laser Type	Current Cost[a]	Lifespan (years)	Total annual discounted costs	Cost per patient[b]
Complete scanning Laser Module TxCell/Haag Streit Fit/ IQ577nm with Micropulse®[c]	£79,800 - purchase price<br>£6,990 – total cost for a 5-year preventative maintenance contract (with the first 2 years being warranty) plus VAT	14	£8,860	£2.95
Nidek GYC-1000 Laser (including installation)[d]	£14,090 - purchase price<br>£3,653 - maintenance over 5 years	7	£3,113	£1.04
Pascal Laser[e]	£51,522 - purchase price (excluding optional extras)<br>£1,548 - maintenance cost	14	£6,266	£2.09
Argon laser (new design high quality ophthalmic laser)	£14,000 – purchase price<br>£809 - maintenance costs	7	£3,010	£1.03

[a] When required, costs were inflated to 2019/20 prices using the NHS Cost Inflation Index (NHSCII). [b] Annual throughput estimate was 3,000: (Personal communication, Noemi Lois, email dated 21 April 2021).[c] Equipment price quotation: Carleton Ltd (via email on 25 July 2016). [d] Equipment price quotation: Birmingham Optical (via email on 25 July 2016). [e] Equipment price quotation: Topcon Ireland Medical (via email on 23 April 2021)
NHS: National Health Service

For peer review only

Supplemental Table A2: VisQol scores at baseline, 12 months and 24 months post-randomisation by VisQol dimension and trial group

Timepoint	VisQol Dimension	Subthreshold Micropulse laser (N = 133)		Standard threshold laser (N = 132)		Between-Group Difference (95% CI)	
		n	Unadjusted mean (SD)	n	Unadjusted mean (SD)		p-value
Baseline	Injure	130	0.953 (0.14)	116	0.967 (0.093)	-0.023 (-0.007 to 0.053)	0.141
	Cope	130	0.942 (0.109)	116	0.956 (0.119)	0.001 (-0.025 to 0.028)	0.915
	Friendships	130	0.915 (0.147)	116	0.940 (0.143)	0.003 (-0.031 to 0.037)	0.879
	Assistance	131	0.970 (0.093)	116	0.983 (0.080)	-0.001 (-0.023 to 0.02)	0.904
	Roles	131	0.927 (0.181)	116	0.961 (0.117)	0.025 (-0.007 to 0.057)	0.119
	Confidence	131	0.936 (0.158)	116	0.970 (0.075)	0.015 (-0.012 to 0.041)	0.269
12 months	Injure	130	0.975 (0.082)	109	0.950 (0.177)	-0.018 (-0.05 to 0.014)	0.272
	Cope	130	0.943 (0.105)	109	0.957 (0.121)	-0.007 (-0.036 to 0.021)	0.614
	Friendships	130	0.918 (0.141)	109	0.944 (0.143)	-0.008 (-0.045 to 0.028)	0.650
	Assistance	129	0.969 (0.117)	110	0.979 (0.086)	-0.007 (-0.030 to 0.015)	0.528
	Roles	129	0.953 (0.121)	110	0.966 (0.083)	-0.005 (-0.039 to 0.029)	0.769
	Confidence	129	0.950 (0.122)	110	0.963 (0.076)	-0.005 (-0.033 to 0.023)	0.710
24 months	Injure	113	0.985 (0.047)	112	0.955 (0.159)	0.004 (-0.029 to 0.037)	0.804
	Cope	112	0.963 (0.099)	111	0.956 (0.159)	0 (-0.030 to 0.028)	0.963
	Friendships	130	0.918 (0.127)	111	0.939 (0.136)	-0.005 (-0.042 to 0.032)	0.805
	Assistance	112	0.991 (0.043)	113	0.977 (0.138)	-0.002 (-0.0248 to 0.021)	0.880
	Roles	113	0.966 (0.113)	113	0.953 (0.138)	-0.012 (-0.047 to 0.022)	0.478
	Confidence	113	0.975 (0.046)	112	0.954 (0.121)	-0.009 (-0.037 to 0.020)	0.546

Injure: Likely to injure self; Cope: Coping with life demands; Friendships: Ability to have friendships; Assistance: Organising assistance; Roles: Difficult to fulfil roles; Confidence: Confidence to join activities.
CI: confidence interval.

For peer review only

Supplemental Table A3: NEI-VFQ-25 Subscale and Composite Scores in participants treated with micropulse subthreshold laser vs. standard threshold laser

Trial group by timepoint	Variable	Observations	Mean	Standard deviation	Minimum	Maximum
**Subthreshold Micropulse laser**						
Baseline	*NEI-VFQ-25 Composite Score*	*131*	*86.38*	*13.83*	*30.88*	*100*
	General Health	131	46.95	25.39	0.00	100
	General Vision	130	72.15	13.75	40.00	100
	Ocular Pain	131	85.02	20.65	0.00	100
	Near Activities	130	80.00	19.84	8.33	100
	Distance Activities	130	87.82	16.09	16.67	100
	Vision Social Function	130	94.62	13.56	37.50	100
	Vision Mental Health	131	82.16	19.95	18.75	100
	Vision Role Difficulties	130	83.85	23.11	0.00	100
	Vision Dependency	131	92.68	19.28	8.33	100
	Driving	92	92.84	12.08	33.33	100
	Color Vision	129	96.71	11.85	50.00	100
	Peripheral Vision	130	88.85	18.95	25.00	100
12 months	*NEI-VFQ-25 Composite Score*	*113*	*89.61*	*9.99*	*45.92*	*100*
	General Health	113	50.00	23.62	0.00	100
	General Vision	112	74.11	13.05	40.00	100
	Ocular Pain	113	89.82	17.40	12.50	100
	Near Activities	113	83.67	19.30	8.33	100
	Distance Activities	112	90.29	15.06	33.33	100
	Vision Social Function	113	96.68	8.35	50.00	100
	Vision Mental Health	113	86.06	15.38	18.75	100
	Vision Role Difficulties	112	86.27	20.95	0.00	100
	Vision Dependency	113	96.31	12.35	25.00	100
	Driving	77	94.53	9.57	50.00	100
	Color Vision	113	98.45	8.37	25.00	100
	Peripheral Vision	112	93.08	14.32	50.00	100
24 months	*NEI-VFQ-25 Composite Score*	*114*	*87.19*	*14.08*	*22.65*	*100*
	General Health	114	52.85	29.33	0.00	100
	General Vision	114	72.63	15.57	20.00	100
	Ocular Pain	114	88.38	16.61	25.00	100
	Near Activities	114	81.18	21.55	0.00	100
	Distance Activities	114	87.35	17.57	12.50	100
	Vision Social Function	113	95.24	12.87	25.00	100
	Vision Mental Health	113	83.13	20.22	0.00	100
	Vision Role Difficulties	113	85.07	24.31	0.00	100
	Vision Dependency	111	93.09	19.72	0.00	100
	Driving	81	91.82	13.57	16.67	100
	Color Vision	110	97.73	9.90	50.00	100
	Peripheral Vision	113	88.50	20.60	25.00	100
**Standard threshold laser**						

Baseline	*NEI-VFQ-25 Composite Score*	*130*	*87.00*	*12.73*	*44.63*	*100*
	General Health	130	51.92	25.22	0.00	100
	General Vision	130	72.92	15.52	40.00	100
	Ocular Pain	130	85.38	17.82	25.00	100
	Near Activities	130	80.16	19.33	25.00	100
	Distance Activities	130	89.01	14.17	41.67	100
	Vision Social Function	130	93.94	13.68	25.00	100
	Vision Mental Health	130	80.87	20.62	6.25	100
	Vision Role Difficulties	129	82.75	26.48	0.00	100
	Vision Dependency	130	93.72	14.57	33.33	100
	Driving	91	95.05	9.38	50.00	100
	Color Vision	130	96.35	11.69	25.00	100
	Peripheral Vision	130	91.54	16.63	25.00	100
12 months	*NEI-VFQ-25 Composite Score*	*117*	*88.47*	*13.78*	*29.21*	*100*
	General Health	116	49.78	25.00	0.00	100
	General Vision	116	75.17	14.59	40.00	100
	Ocular Pain	117	88.25	15.25	37.50	100
	Near Activities	117	83.62	19.29	8.33	100
	Distance Activities	117	88.89	17.02	25.00	100
	Vision Social Function	116	94.29	14.22	25.00	100
	Vision Mental Health	117	86.38	18.99	6.25	100
	Vision Role Difficulties	117	85.26	24.27	0.00	100
	Vision Dependency	116	92.74	19.37	0.00	100
	Driving	81	93.26	14.65	33.33	100
	Color Vision	115	98.48	6.86	50.00	100
	Peripheral Vision	115	91.96	16.07	25.00	100
24 months	*NEI-VFQ-25 Composite Score*	*115*	*88.80*	*13.78*	*29.08*	*100*
	General Health	115	51.96	24.59	0.00	100
	General Vision	114	74.39	14.94	20.00	100
	Ocular Pain	115	89.35	15.55	37.50	100
	Near Activities	115	82.79	20.03	16.67	100
	Distance Activities	115	89.53	17.41	16.67	100
	Vision Social Function	115	94.57	13.05	12.50	100
	Vision Mental Health	115	85.43	21.26	0.00	100
	Vision Role Difficulties	114	86.73	21.76	0.00	100
	Vision Dependency	114	94.01	19.03	0.00	100
	Driving	78	96.42	7.35	58.33	100
	Color Vision	115	96.52	11.89	25.00	100
	Peripheral Vision	115	91.96	16.74	25.00	00

For peer review only

Supplemental Table A4: Frequency of laser treatments and use of anti-VEGFs or steroids as rescue treatment over the 24-month follow-up period

	Subthreshold Micropulse Laser	Standard Threshold Laser	Difference (95% CI)	P-value
**Number of laser treatments used from baseline to month 24 in study eye [a b]**	2.37 (0.11) n=133	1.89 (0.11) n=132	0.48 (0.18, 0.79)	0.002
**Number of patients with at least one steroid injection in study eye (as additional treatment) from baseline to month 24[c]**	0 (0.0%)	1 (0.8%)		
**Number of patients receiving at least one anti-VEGF treatment (as additional treatment) from baseline to month 24[c]**	24 (18.1%) n=133	28 (21.2%) n=132	OR: 0.78 (0.42 – 1.45)	0.44
			% point difference: -2.8 (-13.1 – 7.5)	0.59
**Number of anti-VEGF treatments (as additional treatment) from baseline to month 24[a d]**	0.80 (0.23) n=133	1.30 (0.23) n=132	-0.50 (-1.14 – 0.14)	0.13
**Number of anti-VEGF treatments (as additional treatment) from baseline to month 24 [d e]**				
**1-2**	4 (16.7%) n=24	7 (25.0%) n=28		
**3-4**	10 (41.7%) n=24	7 (25.0%) n=28		
**5-10**	10 (41.7%) n=24	9 (32.1%) n=28		
**>10**	0 (0.0%) n=24	5 (17.9%) n=28		

[a] Mean (SE) presented for continuous outcomes [b] Number of laser treatments, were analysed using linear regression with adjustment for baseline BCVA and minimisation variables. [c] number of patients receiving at least one additional treatment (defined as at least one anti-VEGF or steroid), were analysed using logistic regression models with adjustment for the minimisation variables. [d] Number of steroid injections and number of anti-VEGF treatments (as mean and number (%) in categories), were analysed using linear regression with adjustment for minimisation variables. [e]n (%) based on no. of patients receiving anti VEGF treatments.

anti-VEGF: anti-vascular endothelial growth factor; CI: confidence interval; OR: odds ratio; RR: risk ratio

view only

CHEERS Checklist

Topic	No.	Item	Location where item is reported
**Title**			
	1	Identify the study as an economic evaluation and specify the interventions being compared.	1
**Abstract**			
	2	Provide a structured summary that highlights context, key methods, results, and alternative analyses.	4
**Introduction**			
Background and objectives	3	Give the context for the study, the study question, and its practical relevance for decision making in policy or practice.	6
**Methods**			
Health economic analysis plan	4	Indicate whether a health economic analysis plan was developed and where available.	6
Study population	5	Describe characteristics of the study population (such as age range, demographics, socioeconomic, or clinical characteristics).	6
Setting and location	6	Provide relevant contextual information that may influence findings.	6
Comparators	7	Describe the interventions or strategies being compared and why chosen.	6
Perspective	8	State the perspective(s) adopted by the study and why chosen.	6
Time horizon	9	State the time horizon for the study and why appropriate.	6
Discount rate	10	Report the discount rate(s) and reason chosen.	7
Selection of outcomes	11	Describe what outcomes were used as the measure(s) of benefit(s) and harm(s).	8-9
Measurement of outcomes	12	Describe how outcomes used to capture benefit(s) and harm(s) were measured.	8-9
Valuation of outcomes	13	Describe the population and methods used to measure and value outcomes.	8-9

For peer review only - http://bmjopen.bmj.com/site/about/guidelines.xhtml

Topic	No.	Item	Location where item is reported
Measurement and valuation of resources and costs	14	Describe how costs were valued.	8
Currency, price date, and conversion	15	Report the dates of the estimated resource quantities and unit costs, plus the currency and year of conversion.	7
Rationale and description of model	16	If modelling is used, describe in detail and why used. Report if the model is publicly available and where it can be accessed.	N/A
Analytics and assumptions	17	Describe any methods for analysing or statistically transforming data, any extrapolation methods, and approaches for validating any model used.	9
Characterising heterogeneity	18	Describe any methods used for estimating how the results of the study vary for subgroups.	N/A
Characterising distributional effects	19	Describe how impacts are distributed across different individuals or adjustments made to reflect priority populations.	N/A
Characterising uncertainty	20	Describe methods to characterise any sources of uncertainty in the analysis.	10
Approach to engagement with patients and others affected by the study	21	Describe any approaches to engage patients or service recipients, the general public, communities, or stakeholders (such as clinicians or payers) in the design of the study.	N/A
Results			
Study parameters	22	Report all analytic inputs (such as values, ranges, references) including uncertainty or distributional assumptions.	1 (Suppl A1)
Summary of main results	23	Report the mean values for the main categories of costs and outcomes of interest and summarise them in the most appropriate overall measure.	10-13
Effect of uncertainty	24	Describe how uncertainty about analytic judgments, inputs, or projections affect findings. Report the effect of choice of discount rate and time horizon, if applicable.	13
Effect of engagement with patients and others affected by the study	25	Report on any difference patient/service recipient, general public, community, or stakeholder involvement made to the approach or findings of the study	N/A
Discussion			

For peer review only - http://bmjopen.bmj.com/site/about/guidelines.xhtml

Topic	No.	Item	Location where item is reported
Study findings, limitations, generalisability, and current knowledge	26	Report key findings, limitations, ethical or equity considerations not captured, and how these could affect patients, policy, or practice.	13-14
**Other relevant information**			
Source of funding	27	Describe how the study was funded and any role of the funder in the identification, design, conduct, and reporting of the analysis	2
Conflicts of interest	28	Report authors conflicts of interest according to journal or International Committee of Medical Journal Editors requirements.	2

From: Husereau D, Drummond M, Augustovski F, et al. Consolidated Health Economic Evaluation Reporting Standards 2022 (CHEERS 2022) Explanation and Elaboration: A Report of the ISPOR CHEERS II Good Practices Task Force. Value Health 2022;25. doi:10.1016/j.jval.2021.10.008

For peer review only - http://bmjopen.bmj.com/site/about/guidelines.xhtml

Subthreshold micropulse laser versus standard laser for the treatment of central-involving diabetic macular oedema with central retinal thickness of <400μ: a cost-effectiveness analysis from the DIAMONDS trial

Journal:	*BMJ Open*
Manuscript ID	bmjopen-2022-067684.R3
Article Type:	Original research
Date Submitted by the Author:	28-Sep-2023
Complete List of Authors:	Mistry, Hema; University of Warwick; University Hospitals Coventry and Warwickshire NHS Trust<br>Maredza, Mandy ; University of Warwick<br>Campbell, Christina; Northern Ireland Clinical Trials Unit<br>Lois, Noemi; Wellcome-Wolfson Institute for Experimental Medicine, Ophthalmology<br>Study Group, DIAMONDS; Northern Ireland Clinical Trials Unit
**Primary Subject Heading**:	Health economics
Secondary Subject Heading:	Ophthalmology
Keywords:	OPHTHALMOLOGY, Diabetic retinopathy < DIABETES & ENDOCRINOLOGY, HEALTH ECONOMICS

SCHOLARONE™ Manuscripts

I, the Submitting Author has the right to grant and does grant on behalf of all authors of the Work (as defined in the below author licence), an exclusive licence and/or a non-exclusive licence for contributions from authors who are: i) UK Crown employees; ii) where BMJ has agreed a CC-BY licence shall apply, and/or iii) in accordance with the terms applicable for US Federal Government officers or employees acting as part of their official duties; on a worldwide, perpetual, irrevocable, royalty-free basis to BMJ Publishing Group Ltd ("BMJ") its licensees and where the relevant Journal is co-owned by BMJ to the co-owners of the Journal, to publish the Work in this journal and any other BMJ products and to exploit all rights, as set out in our licence.

The Submitting Author accepts and understands that any supply made under these terms is made by BMJ to the Submitting Author unless you are acting as an employee on behalf of your employer or a postgraduate student of an affiliated institution which is paying any applicable article publishing charge ("APC") for Open Access articles. Where the Submitting Author wishes to make the Work available on an Open Access basis (and intends to pay the relevant APC), the terms of reuse of such Open Access shall be governed by a Creative Commons licence – details of these licences and which Creative Commons licence will apply to this Work are set out in our licence referred to above.

Other than as permitted in any relevant BMJ Author's Self Archiving Policies, I confirm this Work has not been accepted for publication elsewhere, is not being considered for publication elsewhere and does not duplicate material already published. I confirm all authors consent to publication of this Work and authorise the granting of this licence.

For peer review only

Subthreshold micropulse laser versus standard laser for the treatment of central-involving diabetic macular oedema with central retinal thickness of <400μ: a cost-effectiveness analysis from the DIAMONDS trial

Hema Mistry [1,5*], Mandy Maredza[2], Christina Campbell [3], Noemi Lois[4] on behalf of the DIAMONDS study group.

Affiliations

[1] Warwick Clinical Trials Unit, Warwick Medical School, University of Warwick, Coventry, CV4 7AL, UK.

[2] Division of Health Sciences, Warwick Medical School, University of Warwick, Coventry, CV4 7AL, UK.

[3] Northern Ireland Clinical Trials Unit, 7 Lennoxvale, Belfast, BT9 5BY, UK.

[4] Wellcome-Wolfson Institute for Experimental Medicine, Queen's University Belfast, 97 Lisburn Road, Belfast, BT9 7BL, UK.

[5]University Hospitals Coventry and Warwickshire NHS Trust, Clifford Bridge Road, Coventry, CV2 2DX, UK

Correspondence to: Dr Hema Mistry, Warwick Clinical Trials Unit, Warwick Medical School, University of Warwick, Coventry, UK; Email: Hema.Mistry@warwick.ac.uk; Tel: +44 (0)2476 151183

Key words:

Ophthalmology, Diabetic retinopathy, Diabetes & Endocrinology, Health Economics

For peer review only

Abstract (currently 297 words, 300 words max)

Objectives

To estimate the economic costs, health-related quality of life outcomes and cost-effectiveness of subthreshold micropulse laser (SML) versus standard laser (SL) for the treatment of diabetic macular oedema (DMO) with central retinal thickness (CRT) of <400μ.

Design

An economic evaluation was conducted within a pragmatic, multicentre, randomised clinical trial, DIAMONDS.

Setting

18 UK Hospital Eye Services.

Participants

Adults with diabetes and centre involving DMO with CRT <400μ.

Interventions

Participants (n=266) were randomised 1:1 to receive SML or SL.

Methods

The base-case used an intention-to-treat approach conducted from a UK National Health Service (NHS) and personal social services (PSS) perspective. Costs (2019–2020 prices) were collected prospectively over the 2-year follow-up period. A bivariate regression of costs and quality-adjusted life-years (QALYs), with multiple imputation of missing data, was conducted to estimate the incremental cost per QALY gained and the incremental net monetary benefit of SML in comparison to SL. Sensitivity analyses explored uncertainty and heterogeneity in cost-effectiveness estimates.

Results

One participant in the SL arm withdrew consent for data to be used; data from the remaining 265 participants were included in analyses. Mean (standard error [SE]) NHS and PSS costs over 24 months were £735.09 [£111.85] in the SML arm versus £1099.70 [£195.40] in the SL arm (p=0.107). Mean (SE) QALY estimates were 1.493 [0.024] versus 1.485 [0.020], respectively (p=0.780), giving an

For peer review only

insignificant difference of 0.008 QALYs. The probability SML is cost-effective at a threshold of £20,000 per QALY was 76%.

Conclusions

There were no statistically significant differences in EQ-5D-5L scores or costs between SML and SL. Given these findings and the fact that SML does not burn the retina, unlike SL, and has equivalent efficacy to SL, it may be preferred for the treatment of people with DMO with CRT <400μ.

Trial registration number

ISRCTN17742985; NCT03690050

For peer review only

Strengths and limitations of this study

- Study is based on a high-quality randomised clinical trial.
- First study to compare the cost-effectiveness of subthreshold micropulse laser (SML) with that of standard laser (SL) for the treatment of diabetic macular oedema (DMO).
- The analysis was from a UK National Health Service (NHS) and Personal Social Services (PSS) perspective and did not consider costs such as productivity losses from a societal perspective.
- Low rate of missing data for both costs and outcomes.
- We used published costs of anti-VEGF drugs, rather than NHS costs which incorporates confidential price discounts for these drugs.

For peer review only

Manuscript word count 3309 words (including the 3 tables); max 4000 words; up to 5 images/tables

Introduction

Diabetic macular oedema (DMO) is a visual threatening complication of diabetes, occurring in approximately 7% of people living with diabetes.(1) Given the high and continuously increasing prevalence of diabetes,(2) DMO is a frequent eye disease requiring treatment in ophthalmic clinics in the UK and worldwide. DMO can impose a significant social and economic burden on society, due to its high prevalence and associated costs. Very few studies have explored the economic burden of DMO and even fewer have reported on its cost-effectiveness. A cost-of-illness study using cohort data from US Medicare data reported just under 38% with DMO underwent laser photocoagulation and their 1-year mean direct medical costs amounted to US$11,290, 31% higher than for those without DMO.(3) In a cohort study from South Korea, the mean 1-year medical costs were higher for people with DMO (US$6,723) than those who had diabetes without retinopathy.(4) The estimated health care and social care costs for DMO in England in 2010 were £92 million and £11.6 million respectively, with £65.6 million of this being spent on hospital treatment and related costs.(5)

In DMO fluid accumulates in the center of the retina, the macula, which is the area of the retina responsible for providing central vision.(6) The purpose of the treatment is to restore the anatomy of the macula by clearing up this fluid and restoring vision. Treatments include intraocular injections of anti-vascular endothelial growth factor (anti-VEGF) drugs or steroids and macular laser. The National Institute for Health and Care Excellence (NICE) recommends anti-VEGF therapy for people with more severe forms of DMO, with central retinal (macular) thickness (CRT) of 400µ or above, as measured in scans obtained using an imaging modality called optical coherence tomography (OCT).(7-9) For milder forms of DMO (CRT <400µ) NICE recommends macular laser. Intravitreal steroids are advised for people that do not respond to the above-mentioned therapies.

For peer review only

DIAbetic Macular Oedema aNd Diode Subthreshold micropulse laser (DIAMONDS) was a pragmatic, allocation-concealed, double-masked, multicentre, randomised, non-inferiority clinical trial which compared the clinical effectiveness and cost-effectiveness of subthreshold micropulse laser (SML) and standard laser (SL) for the treatment of people with DMO with CRT of <400µ.(10, 11) DIAMONDS participants were randomised 1:1 to receive SML (577 nm) or SL (e.g. using argon, frequency-doubled neodymium-doped yttrium aluminium garnet [Nd:YAG] 532 nm laser). Laser treatment could be repeated as needed, using the allocated laser at randomisation and rescue treatment with anti-VEGFs or steroids was allowed. The primary outcome was the mean change in best-corrected visual acuity (BCVA) in the study eye at 24 months. DIAMONDS found SML and SL to have equivalent clinical efficacy.(10, 11) This finding is clinically important given the fact that SML, unlike SL, does not cause any functional or structural damage to the retina(12-14) and, thus, may be preferred by patients and doctors. Herein, we present the detailed within-trial economic evaluation comparing costs and benefits of the two laser modalities, SML and SL. To our knowledge, no other trials have compared the cost-effectiveness of SML and SL for the treatment of DMO before.

Methods

Patient and Public Involvement

As described previously(10, 11): *"At the very early stages of the DIAMONDS trial conception, a DIAMONDS Patient and Public Involvement (PPI) group was established with the help of the Northern Ireland branch of DIABETES UK. The DIAMONDS PPI group comprised people living with diabetes and DMO, including a large group of members of the "Diabetes Family" Facebook group. The DIAMONDS PPI group contributed to the trial design and the research question, including the selection of outcomes, preparation of patient related materials for the trial, recruitment strategies, interpretation of trial results, and preparation of the plain English summary. They also have a major role in the dissemination and implementation of trial results."*(10, 11)

For peer review only

The study is reported as per Consolidated Health Economic Evaluation Reporting Standards (CHEERS) 2022 Statement.(15) As detailed in the Health Economics Analysis Plan (HEAP)(16) we planned to conduct a within-trial analysis comparing the cost-effectiveness of SML with SL. The protocol for the DIAMONDS trial envisaged that economic modelling might be required if visual outcomes differed between arms.(17) The DIAMONDS protocol was designed to minimise visual loss in participants and, thus, repeating laser treatment or undertaking rescue treatment with intravitreal anti-VEGF drugs and/or steroids if criteria for rescue were met, were allowed in either arm of the trial. DIAMONDS recruited 266 participants, 116 (87%) in the SML arm and 115 (86%) in the SL arm had primary outcome data, fulfilling the requirements of the power calculation (113 participants with BCVA data at month 24 were required). DIAMONDS found SML and SL to be equivalent in terms of the primary outcome.(10, 11) Hence, economic modelling was not required. Herein, we report detailed economic costs, health-related quality of life (HRQoL) outcomes and cost-effectiveness of SML versus SL.

As described in detail previously in Lois *et al* (2022)(10) and Lois *et al* (2023)(11), the methods (resource use, costs and outcomes) have been summarised here. The economic evaluation took the form of a cost-utility analysis, expressed in terms of cost per quality-adjusted life-year (QALY) gained. The study was conducted over the 24-month time horizon of the trial and adopted a National Health Service (NHS) and personal and social service (PSS) perspective. Costs and outcomes in the second year of follow-up were discounted at 3.5% in line with the NICE reference case.(18)

Resource use data were collected and reported on trial case record forms (CRFs) at scheduled 4-monthly clinic visits (4, 8, 12, 16, 20 and 24 months). Data were collected on the costs of laser treatment, both at the initial laser session and at subsequent ones if required, outpatient visits, and intravitreal anti-VEGF and/or steroid treatment (costs of drugs and administration) if rescue was

For peer review only

required. All costs were expressed in UK pounds sterling and valued in 2019–20 prices. If costs were not in line, they were inflated to 2019-20 prices using the NHS Cost Inflation Index (NHSCII).(10, 19)

The costs of laser treatment included staff and equipment costs (capital and maintenance costs of laser machines). Unit costs for staff were obtained from the Unit Costs of Health and Social Care 2019 compendium (19) and were based on the time it took to for each procedure to be undertaken. These times were recorded on CRFs and included: (i) the time taken to obtain fundus fluorescein angiography (FFA) and spectral domain optical coherence tomography (SD-OCTs) scans to guide laser treatment (if used) and (ii) time taken by ophthalmologists to perform the laser procedure, including counselling the participant. Costs of laser machines were obtained from manufacturers. An annual equivalent cost of equipment was obtained by annuitising the capital costs of the item over its useful life span and applying a discount rate of 3.5% per annum.(10) A per-patient cost of equipment was estimated by assuming that the laser machines were used to treat 3,000 patients per year.(10, 11)

Data were also collected on any outpatient attendances or hospital admissions related to DMO or the treatments. Where an outpatient attendance was reported but no procedure undertaken, the average unit cost of an outpatient ophthalmology visit was used (varying between £80-£101 per consultation depending on whether the consultation was 'non-consultant' versus 'consultant-led').(10, 20) The CRFs recorded data on the grade of professional that attended the patient, for example, if a consultant attended to the patient, then the consultant-led unit cost was applied. Where a procedure(s) was undertaken as part of the visit the relevant HRG code was derived using the HRG4+ Reference Costs Grouper Software (NHS Digital, Leeds, UK).(10)

CRFs also recorded information on other tests or investigations, medication use including anti-VEGF/steroids or other rescue treatments. Anti-VEGF and steroid drugs were separately costed as

For peer review only

these are considered an unbundled HRG. Costing of laser retreatments followed the same approach as costing for the index (first session) laser procedure.

Unit costs were derived from national compendia in accordance with NICE's Guide to the Methods of Technology Appraisal.(18) The key databases included the Department of Health and Social Care's Reference Costs 2018–19 schedules,(20) the PSSRU's Unit Costs of Health and Social Care 2020 compendium (19), 2020 volume of the British National Formulary.(21) Supplemental Table A1 gives a summary of the unit costs for resource use and the laser equipment. Resource inputs were valued by attaching unit costs.

The HRQoL of trial participants was assessed at baseline and at 12- and 24-months post-randomisation using the EQ-5D-5L instrument.(10) The EQ-5D-5L questionnaire was used to generate QALYs for the cost-effectiveness analysis. (22) The QALY is a measure that combines quantity and quality of life lived into a single metric, with one QALY equating to one year of full health. To convert EQ-5D-5L responses into health utility scores we used the EQ-5D-5L Crosswalk Index Value Calculator which maps the EQ-5D-5L descriptive system data onto the EQ-5D-3L valuation set. This valuation set was recommended by NICE at the time when the analysis was undertaken.(22)

HRQoL was also assessed using two vision specific measures: the National Eye Institute Visual Functioning Questionnaire - 25 (NEI-VFQ-25) and Vision and Quality of Life Index (VisQoL).(23-25) The NEI-VFQ-25 is a validated questionnaire that has been used widely to evaluate visual outcomes in patients with eye diseases including diabetic retinopathy and DMO. The VisQol questionnaire has not been widely validated but is shorter than the NEI-VFQ-25 with only six attributes (physical well-being, independence, social well-being, self-actualisation, planning and organisation). The utilities for VisQoL were developed using a time-trade off exercise in people who were visually impaired

For peer review only

which included patients with age-related macular degeneration, diabetic retinopathy and glaucoma.(25)

Summary statistics were generated for resource use variables, health utility values and QALYs by treatment allocation and assessment time point. For resource use and costs, mean values were compared between groups using two sample t-tests. Differences between groups, along with 95% confidence intervals (CIs) were estimated using non-parametric bootstrap estimates (10,000 replications). For HRQoL, these were presented as mean values with their associated standard errors. Between-group differences were compared using the two-sample t-test.(10, 11)

Multiple imputation by chained equations was used to predict missing health status (utility) scores and costs based on the assumption that data were missing at random (MAR).(10) Twenty imputed data sets were generated and used to inform the base-case analyses. Parameter estimates were pooled across the 20 imputed data sets using Rubin's rules to account for between- and within-imputation components of variance terms associated with parameter estimates.(26)

The base-case cost-effectiveness analysis was performed using an intention-to-treat approach. Mean incremental costs and QALYs were estimated using seemingly unrelated regression (SUR) methods that account for the correlation between costs and outcomes. The SUR adjusted for covariates (baseline utilities, baseline body mass index (BMI), baseline best corrected distance visual acuity (BCVA), patient-reported previous use of anti-VEGF at baseline and previous use of macular laser).

Results

For peer review only

As reported elsewhere (10, 11) there was no difference in the clinical effectiveness of SML and SL. Table 1 shows that there were also no statistically significant differences between laser groups in EQ-5D-5L scores at baseline, 12 and 24 months.

Table 1: EQ-5D-5L utility scores at baseline, 12 and 24 months and QALYs (base-case, imputed analysis)

Variable	Subthreshold micropulse laser (n = 133)	Standard laser (n = 132)	Between-Group Difference (95% CI)	p-value
	Mean (SE)	Mean (SE)		
**EQ-5D Utility Scores***				
Baseline	0.758 (0.267)	0.772 (0.226)	-0.014 (-0.074 to 0.046)	0.640
12 months	0.767 (0.250)	0.758 (0.017)	0.009 (-0.041to 0.059)	0.717
24 months	0.739 (0.278)	0.743 (0.279)	-0.004 (-0.064 to 0.056)	0.897
**EQ-5D-5L QALYs**				
Over 2 years	1.493 (0.024)	1.485 (0.024)	0.008 (-0.061 to 0.075)	0.836

*Analysis adjusted for participant age, gender, baseline BCVA (Best Corrected Distance Visual Acuity) and participant's previous use of Anti-VEGF (Anti-Vascular Endothelial Growth Factor) and laser therapy at baseline, with repeated measures within participant and site.
SE = standard error; CI = confidence interval

Furthermore, there were no statistically significant differences between the two laser treatment groups for any of the VisQoL dimensions or NEI-VFQ-25 subscales at any follow-up time point (Supplemental Table A2 and Supplemental Table A3, respectively).(10, 11)

The mean numbers of laser treatments performed were 2.4 in the SML and 1.9 in the SL arm. This difference was statistically significant (p=0.002) (Supplemental Table A4), but equated to less than one further session of laser in the SML arm when compared with the SL arm during the 2-year trial. Furthermore, this difference was driven by a small number of participants requiring a higher number of lasers sessions in the SML group (13 participants required 6 or 7 laser treatments in the SML arm

For peer review only

compared to 2 in the SL arm). Eighteen percent of participants in the SML arm and 21% in the SL arm required rescue treatments in the study eye (almost all with anti-VEGF drugs; in addition, only one participant had a steroid injection). The average numbers of anti-VEGF treatments per arm from baseline to 24 months were 1.06 in the SML arm and 1.96 in the SL (Supplemental Table A4). The number in the SL arm was skewed by five participants who received 10 or more injections.

Table 2 shows the NHS costs associated with resource use in the base-case (imputed) analysis by cost category and follow-up period. Costs of the first laser procedure are reported separately from those of subsequent laser retreatments. The total costs of laser therapy for each participant includes costs of the first laser procedure plus any subsequent laser retreatments they had. The mean (standard error (SE)) cost for the first laser procedure (including costs of performing OCT and FFA to guide laser treatment, if done) was £45.59 (1.64) for the SML compared with £42.29 (1.69) for the SL; the difference was not statistically significant(p=0.09). The mean total NHS and PSS costs were lower in SML compared with the SL (£735.09 vs. £1099.70) between baseline to 24 months post-randomisation; this difference was not statistically significant at the 5% level. The CI around total costs were wide and overlapped and the difference was driven by the higher number of anti-VEGF rescue injections.

For peer review only

Table 2: *Economic costs by trial allocation arm and cost component category for the entire follow-up period in base-case (imputed) analysis (£, 2019-20 prices)*

**Parameter**	**Subthreshold Micropulse laser Mean Costs (SE)**	**Standard threshold laser Mean Costs (SE)**	**Mean Difference**	**Bootstrap 95% CI**
Index Laser Procedure	45.59 (1.64)	42.29 (1.69)	3.31	(-1.33 to 7.95)
Laser retreatments	53.02 (5.17)	41.69 (4.42)	11.32	(-2.08 to 24.73)
Outpatient care	124.85 (22.56)	130.32 (31.58)	-5.47	(-81.92 to 70.97)
Anti-VEGF drug costs	511.63 (105.85)	885.40 (183.29)	-373.77	(-791.01 to 43.48)
**Total NHS and PSS Costs**	**735.09 (111.85)**	**1,099.70 (195.40)**	**-364.61**	**(-807.09 to 77.87)**
SE = standard error; CI = confidence interval				

Over the 2-year follow-up period, participants in the SML arm, compared to the SL arm, experienced a non-statistically significant increase in QALYs of 0.008 (circa 3 days of good quality of life).(10, 11) In addition, the mean NHS and PSS costs were lower in the SML arm compared to the SL arm (mean cost difference –£365) – see Table 3. The confidence interval for the cost difference was wide and ranged from cost saving to cost increasing. Although neither costs nor benefits were statistically significantly different between SML and SL, the incremental cost-effectiveness ratio (ICER) for the base-case imputed analysis indicated that SML dominates, as average costs for this intervention were slightly lower and average benefits marginally higher (but not significantly so) than those for SL. Assuming cost-effectiveness threshold of £20,000 per QALY, the probability that SML was cost-effective was 0.76, and the NMB associated with SML was positive. Figure 1 shows the joint distributions of costs and outcomes. The graph also highlights that SML has the potential to be cost saving as the majority of the bootstrapped iterations lie in the bottom half of the cost-effectiveness plane.

Table 3: Cost-effectiveness, cost/QALY (£, 2020): SML compared to SL

**Mean incremental cost (95% CI)**	**Mean incremental QALY (95% CI)**	**ICER**	**Probability of cost-effectiveness***	**Net Monetary Benefit (NMB)***
Base-case analysis - ITT approach: Imputed attributable costs and QALYs, covariate adjusted[a]				
-365 (-822 to 93)	0.008 (-0.059 to 0.075)	Dominant	0.763	520 (-925 to 1,965)

For peer review only

Mean incremental cost (95% CI)	Mean incremental QALY (95% CI)	ICER	Probability of cost-effectiveness*	Net Monetary Benefit (NMB)*
***Cost-effectiveness threshold is at £20,000/QALY threshold**				
[a] Adjusted for baseline EQ-5D utility, BMI and minimisation variables at baseline (best corrected distance visual acuity and previous use of laser treatment)				
CI = confidence interval; ICER = incremental cost-effectiveness ratio; ITT = intention-to-treat approach; QALY = quality-adjusted life year				

Discussion

DIAMONDS was a pragmatic clinical trial carried out in 16 NHS ophthalmology departments throughout the UK. The DIAMONDS trial was powered to detect not only differences in the primary outcome (BCVA) but also in important secondary outcomes (CRT and vision quality of life).(10, 11) However, a limitation of this cost-effectiveness analysis is that we did not use BCVA as the primary outcome, instead we used QALYs as the main outcome. Participants were treated and followed as per routine clinical care. Costs and outcome data were collected prospectively. We found no significant differences in EQ-5D-5L and only a trivial non-significant difference of 0.008 in a calculation of QALYs. So, the verdict on whether one form of laser is better than the other shifts the focus onto the costs. This analysis found that costs were slightly higher (but not statistically significantly so) in the SL arm compared to the SML arm, due to more participants in the SL arm needing higher numbers (10 or more) of anti-VEGF rescue injections. Reporting both the costs and QALYs together in a ratio form, this meant the ICER for the base-case analysis indicated that SML is the dominant procedure, as average costs for this intervention were lower and average benefits were marginally higher than those for SL. However, caution should be taken when interpreting the results, given the wide confidence intervals around the mean costs (which ranges from cost saving to cost increasing). Taking this into consideration, costs of SML and SL treatment arms seem comparable. We also conducted a per-protocol analysis as part of a sensitivity analysis as DIAMONDS was a non-inferiority trial. The results were in line with the intention-to-treat analysis,

For peer review only

were SML remained the dominant option and the probability of of SML being cost-effectiveness at the £20k/QALY threshold was 0.773.

As noted in the main clinical effectiveness manuscript(11) there may be advantages of SML over SL. , Among these and importantly, the fact that SML does not cause any functional or structural damage to the retina(12-14) and, thus, can be repeated as needed. This is an advantage for patients as their retina will remain intact despite the application of SML but would lose cells as a result of the laser burn if SL is applied. Furthermore, the lack of a burn following SML makes the delivery of the treatment safer in less experienced hands as, unlike SL, it does not carry a risk of burning the fovea. Furthermore, for the same reason, it may allow training of allied non-medical staff to undertake this procedure, who currently, are already undertaking anti-VEGF injections in the UK and other parts of the world. This would help alleviate clinical capacity constraints experienced by most ophthalmology units across the world. The clinical effectiveness results reported previously(10, 11) and the HRQoL results presented here showed that SML is as clinically effective as SL at a similar cost.

In DIAMONDS, two vision-specific patient reported outcome quality of life instruments were used in addition to the generic preference-based EQ-5D-5L. Neither showed any statistically significant differences between laser arms. It has sometimes been mentioned that changes in vision that are sufficient to affect some activities of daily living, may not be reflected in changes in EQ-5D, because the EQ-5D may not be as sensitive to detect changes in quality of life as other visual specific questionnaires such as the NEI-VFQ-25.(7, 27, 28) A mapping exercise from DIAMONDS data is underway to explore this important aspect.

One limitation in the current analysis is the fact that we had to use published costs of anti-VEGF drugs (at list prices, these can range from £5 to £7k a year),(7-9) rather than costs to the NHS. We

For peer review only

know that there are confidential price discounts for these drugs when used in the NHS (without taking into account the administration of the injection into the eye and monitoring visits) and this will most likely reduce the cost differential between the two arms.(7-9) Another limitation is that DIAMONDS included only patients with CRT <400µ, so extrapolations with regard to the cost-effectiveness of SML when compared with SL in thicker retinas, where standard laser is known to be less effective, cannot be made. Furthermore, the analysis was conducted from a NHS and PSS perspective, so we have not taken into account any broader societal costs such as time off work or care for children or other dependents, when patients have laser treatment.

Both SML and SL were successful in 80% of participants.(11) Macular laser treatment is known to be less expensive than anti-VEGF drugs and may be more convenient and acceptable to participants, although no trials have been conducted comparing the cost-effectiveness of macular laser with anti-VEGFs in people with <400µ CRT DMO. Thus, SML should be considered as first line therapy for patients with central involving DMO with CRT of <400µ.

Contributor statement/Acknowledgements

The Authors would like to thank all the DIAMONDS participants and the DIAMONDS patient and public involvement (PPI) group. We also thank Dr Pamela Royle for literature searches. We would also like to thank Iridex for providing a free loan for the laser equipment for the trial and William Moore, Joan Stauffer, George Marcellino (*in memoriam*), Gareth Hymas, Nick Fitrzyk, and Suzanne Kelly, for their support. Iridex had no role in the design, data analysis or interpretation of the DIAMONDS data or in the writing of this report. We thank also the NIHR HTA programme for providing funding for the trial and note that the views expressed in this paper are those of the authors and not necessarily those of the NHS, the NIHR or the Department of Health and Social Care.

For peer review only

Author contributions

Hema Mistry contributed to the design of the health economics plan and provided oversight of all aspects of the economic evaluation including its design, conduct, analysis and reporting of this paper.

Mandy Maredza (University of Warwick) conducted the analysis and reporting of the economic evaluation and contributed to the drafting of this paper.

Christina Campbell (Trial Statistician, NICTU) performed the statistical analyses, assisted with interpretation of data and contributed to the drafting of this paper.

Professor Noemi Lois (Clinical Professor of Ophthalmology, Wellcome-Wolfson Institute for Experimental Medicine, Queen's University Belfast) is the Chief Investigator for the DIAMONDS trial conceived the trial, with input from the DIAMONDS Study Group, and led it to its successful completion. In addition, Prof Lois contributed with the identification, recruitment, examination and treatment of all participants enrolled at the Belfast site, as well as to data collection, analysis and interpretation of the trial findings. She contributed to the drafting of this paper.

DIAMONDS Study Group

Noemi Lois, Queen's University and Royal Victoria Hospital, Belfast H&SC Trust; Augusto Azuara-Blanco, Queen's University and Royal Victoria Hospital, Belfast H&SC Trust; Christina Campbell, NICTU, Belfast; Danny McAuley, Queen's University and Royal Victoria Hospital, Belfast H&SC Trust; Mandy Maredza, Warwick University; Hema Mistry, Warwick University; Norman Waugh, Warwick University; Nachiketa Acharya, Sheffield Teaching Hospitals NHS Foundation Trust; Tariq M Aslam, Manchester Royal Eye Hospital, Central Manchester University Hospitals NHS Foundation Trust; Clare Bailey, Bristol Eye Hospital, University Hospitals Bristol NHS Foundation Trust; Victor Chong, Royal Free Hospital NHS Foundation Trust, London; Louise Downey, Hull and East Yorkshire Hospital, Hull and East Yorkshire NHS Trust; Haralabos Eleftheriadis, Kings College Hospital NHS Foundation

For peer review only

Trust; Samia Fatum, John Radcliffe Hospital, Oxford University Hospitals NHS Foundation Trust; Sheena George, Hillingdon Hospitals NHS Foundation Trust; Faruque Ghanchi, Bradford Teaching Hospitals NHS Trust; Markus Groppe, Stoke Mandeville Hospital, Buckinghamshire NHS Trust; Robin Hamilton, Moorfields Eye Hospital NHS Foundation Trust; Geeta Menon, Frimley Park Hospital NHS Foundation Trust; Ahmed Saad, James Cook University Hospital, South Tees Hospitals NHS Foundation Trust; Sobha Sivaprasad, Moorfields Eye Hospital NHS Foundation Trust; Marianne Shiew, Hinchingbrooke Hospital North West Anglia NHS Trust; David H Steel, Sunderland Eye Infirmary, City Hospitals Sunderland NHS Foundation Trust; James Stephen Talks, Newcastle Eye Centre, Royal Victoria Infirmary, Newcastle upon Tyne Hospitals NHS Foundation Trust; Catherine Adams, NICTU, Belfast; Paul Doherty, NICTU, Belfast; Evie Gardener, NICTU, Belfast; Aby Joseph, NICTU, Belfast; Cliona McDowell, NICTU, Belfast; Matthew Mills, NICTU, Belfast; Mike Clarke, Queens University, Belfast.

Competing interests: HM: none; **MM**: none; **CC**: none; **NL**: none.

Funding: DIAMONDS was funded by the Health Technology Assessment (HTA) programme of the National Institute for Health Research (NIHR), United Kingdom NIHR-HTA [13/142/04]. The NIHR had no role in the design or conduct of this research. Iridex provided a free loan for the laser equipment for the trial. Iridex had no role in the design or conduct of the trial or on data analysis or interpretation of the DIAMONDS data or writing of this report.

Data sharing statement

All data requests should be submitted to the corresponding author for consideration.

Ethics statements

Ethics Approval

For peer review only

The protocol for the DIAMONDS trial was approved by ORECNI (15/NI/0197). A Clinical Trial Authorisation (CTA) was obtained from the Medicines and Healthcare products Regulatory Agency (MHRA) (32485/0029/001-0001). The trial was registered with the European Union Drug Regulating Authorities Clinical Trials (EudraCT) database (2015-001940-12) and in the International Standard Randomised Controlled Trial Number (ISRCTN) register (ISRCTN16962255) and clinicaltrials.gov (NCT03690050).

Patient consent for publication

Not applicable

Figure legend/caption

Figure 1: Cost-effectiveness scatterplot with 95% confidence ellipses at 24 months for base-case within-trial analysis (NHS and PSS perspective, imputed, additionally controlled for baseline utilities)

For peer review only

References

1. Yau JW, Rogers SL, Kawasaki R, Lamoureux EL, Kowalski JW, Bek T, et al. Global prevalence and major risk factors of diabetic retinopathy. Diabetes care. 2012;35(3):556-64.
2. Diabetes UK. Number of people with diabetes reaches 4.7 million London2019 [Available from: https://www.diabetes.org.uk/about_us/news/new-stats-people-living-with-diabetes.
3. Shea AM, Curtis LH, Hammill BG, Kowalski JW, Ravelo A, Lee PP, et al. Resource use and costs associated with diabetic macular edema in elderly persons. Archives of ophthalmology. 2008;126(12):1748-54.
4. Jeon HL, Lee H, Yoon D, Lee Y, Kim JH, Jee D, et al. Burden of diabetic macular oedema in patients receiving antivascular endothelial growth factor therapy in South Korea: a healthcare resource use and cost analysis. BMJ Open. 2020;10(12):e042484.
5. Minassian D, Owens DR, Reidy A. Prevalence of diabetic macular oedema and related health and social care resource use in England. British journal of ophthalmology. 2012;96(3):345-9.
6. Stitt AW, Curtis TM, Chen M, Medina RJ, McKay GJ, Jenkins A, et al. The progress in understanding and treatment of diabetic retinopathy. Progress in retinal and eye research. 2016;51:156-86.
7. National Institute for Health and Care Excellence. Ranibizumab for treating diabetic macular oedema (rapid review of technology appraisal guidance 237). London: NICE; 2013 April.
8. National Institute for Health and Care Excellence. Aflibercept for treating diabetic macular oedema. London: NICE; 2015 July.
9. National Institute for Health and Care Excellence. Faricimab for treating diabetic macular oedema. London: NICE; 2022 June.
10. Lois N, Campbell C, Waugh N, Azuara-Blanco A, Maredza M, Mistry H, et al. DIAbetic Macular Oedema aNd Diode Subthreshold micropulse laser (DIAMONDS): A pragmatic, multicentre, allocation concealed, double-masked prospective, randomised, non-inferiority, clinical trial. Health Technology Assessment. 2022;26(50):1-86.
11. Lois N, Campbell C, Waugh N, Azuara-Blanco A, Maredza M, Mistry H, et al. DIAbetic Macular Oedema aNd Diode Subthreshold micropulse laser (DIAMONDS): A pragmatic, double-masked, randomised, non-inferiority trial. Ophthalmology. 2023;130(1):14-27.
12. Luttrull JK, Sinclair SH. Safety of transfoveal subthreshold diode micropulse laser for fovea-involving diabetic macular edema in eyes with good visual acuity. Retina. 2014;34(10):2010-20.
13. Vujosevic S, Martini F, Longhin E, Convento E, Cavarzeran F, Midena E. Subthreshold micropulse yellow laser versus subthreshold micropulse infrared laser in center-involving diabetic macular edema: morphologic and functional safety. Retina. 2015;35(8):1594-603.
14. Wells-Gray EM, Doble N, Ohr MP, Choi SS. Structural integrity of individual cone photoreceptors after short-wavelength subthreshold micropulse laser therapy for diabetic macular edema. Ophthalmic Surgery, Lasers and Imaging Retina. 2018;49(12):946-54.
15. Husereau D, Drummond M, Augustovski F, de Bekker-Grob E, Briggs AH, Carswell C, et al. Consolidated Health Economic Evaluation Reporting Standards 2022 (CHEERS 2022) statement: updated reporting guidance for health economic evaluations. International journal of technology assessment in health care. 2022;38(1):e13.
16. Mistry H, Waugh N, Lois N. DIAMONDS Health Economics Analysis Plan v1.5 2022 [Available from: https://nictu.hscni.net/diamonds-trial-documents/.
17. Lois N, Gardner E, Waugh N, Azuara-Blanco A, Mistry H, McAuley D, et al. Diabetic macular oedema and diode subthreshold micropulse laser (DIAMONDS): study protocol for a randomised controlled trial. Trials. 2019;20(1):1-11.
18. National Institute for Health and Care Excellence. Guide to the methods of technology appraisal. London: NICE; 2013.
19. Curtis L, Burns A. Unit Costs of Health and Social Care 2020. Kent: Personal Social Services Research Unit; 2020.
20. Department of Health. NHS reference costs 2018 to 2019: GOV.UK; 2018 [Available from: https://www.england.nhs.uk/publication/2018-19-national-cost-collection-data-publication/.
21. British Medical Association and Royal Pharmaceutical Society of Great Britain. British National Formulary No. 80, September 2020-March 2021. London: BMA and PRS.
22. Van Hout B, Janssen M, Feng Y-S, Kohlmann T, Busschbach J, Golicki D, et al. Interim scoring for the EQ-5D-5L: mapping the EQ-5D-5L to EQ-5D-3L value sets. Value in Health. 2012;15(5):708-15.

23. Mangione CM, Lee PP, Gutierrez PR, Spritzer K, Berry S, Hays RD. Development of the 25-list-item national eye institute visual function questionnaire. Archives of ophthalmology. 2001;119(7):1050-8.
24. Misajon R, Hawthorne G, Richardson J, Barton J, Peacock S, Iezzi A, et al. Vision and quality of life: the development of a utility measure. Investigative ophthalmology & visual science. 2005;46(11):4007-15.
25. Peacock S, Misajon R, Iezzi A, Richardson J, Hawthorne G, Keeffe J. Vision and quality of life: development of methods for the VisQoL vision-related utility instrument. Ophthalmic epidemiology. 2008;15(4):218-23.
26. Little RJ, Rubin DB. Statistical analysis with missing data: John Wiley & Sons; 2019.
27. Payakachat N, Summers KH, Pleil AM, Murawski MM, Thomas J, Jennings K, et al. Predicting EQ-5D utility scores from the 25-item National Eye Institute Vision Function Questionnaire (NEI-VFQ 25) in patients with age-related macular degeneration. Quality of Life Research. 2009;18:801-13.
28. Malkin AG, Goldstein JE, Perlmutter M, Massof RW. Responsiveness of the EQ-5D to the effects of low vision rehabilitation. Optometry and vision science: official publication of the American Academy of Optometry. 2013;90(8):799.

For peer review only

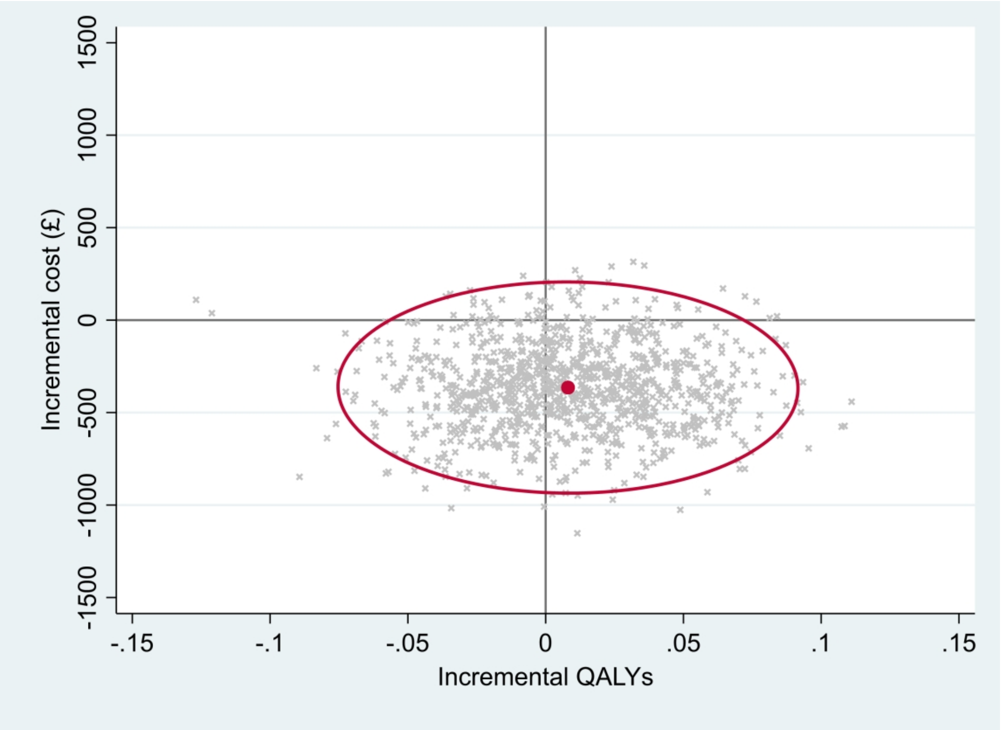

139x101mm (600 x 600 DPI)

Supplemental Tables

Supplemental Table A1: Unit costs

Resource Item	Unit Cost (£, 2020)	Measurement unit	Source
**Staff Costs**			
Consultant	114	per working hour	PSSRU 2020, page 158
Associate specialist/Staff Grade	117	per working hour	PSSRU 2020, page 158
Retina Fellow	50	per working hour	PSSRU 2019, page 158
Ophthalmic photographer/ Imaging technician	52	per working hour	PSSRU 2020, page 148
**Anti-vascular endothelial growth factor costs**			
Ranibizumab	569	Per dose	NHS Reference Costs 2019-20
Aflibercept	634	Per dose	NHS Reference Costs 2019-20
Bevacizumab	277	Per dose	NHS Reference Costs 2019-20

**Laser equipment costs**				
**Laser Type**	**Current Cost[a]**	**Lifespan (years)**	**Total annual discounted costs**	**Cost per patient[b]**
Complete scanning Laser Module TxCell/Haag Streit Fit/ IQ577nm with Micropulse®[c]	£79,800 - purchase price<br>£6,990 – total cost for a 5-year preventative maintenance contract (with the first 2 years being warranty) plus VAT	14	£8,860	£2.95
Nidek GYC-1000 Laser (including installation)[d]	£14,090 - purchase price<br>£3,653 - maintenance over 5 years	7	£3,113	£1.04
Pascal Laser[e]	£51,522 - purchase price (excluding optional extras)<br>£1,548 - maintenance cost	14	£6,266	£2.09
Argon laser (new design high quality ophthalmic laser)	£14,000 – purchase price<br>£809 - maintenance costs	7	£3,010	£1.03

[a] When required, costs were inflated to 2019/20 prices using the NHS Cost Inflation Index (NHSCII). [b] Annual throughput estimate was 3,000: (Personal communication, Noemi Lois, email dated 21 April 2021). [c] Equipment price quotation: Carleton Ltd (via email on 25 July 2016). [d] Equipment price quotation: Birmingham Optical (via email on 25 July 2016). [e] Equipment price quotation: Topcon Ireland Medical (via email on 23 April 2021)

NHS: National Health Service

Reproduced with permission from Lois et al (2022).(1) This is an open access article distributed in accordance with the Creative Commons Attribution 4.0 Unported (CC BY 4.0) license, which permits others to copy, redistribute, remix, transform and build upon this work for any purpose, provided the original work is properly cited, a link to the licence is given, and indication of whether changes were made. See: https://creativecommons.org/licenses/by/4.0/. The table includes minor additions and formatting changes to the original table.

For peer review only - http://bmjopen.bmj.com/site/about/guidelines.xhtml

Supplemental Table A2: VisQol scores at baseline, 12 months and 24 months post-randomisation by VisQol dimension and trial group

Timepoint	VisQol Dimension	Subthreshold Micropulse laser (N = 133)		Standard threshold laser (N = 132)		Between-Group Difference (95% CI)	
		n	Unadjusted mean (SD)	n	Unadjusted mean (SD)		p-value
Baseline	Injure	130	0.953 (0.14)	116	0.967 (0.093)	-0.023 (-0.007 to 0.053)	0.141
	Cope	130	0.942 (0.109)	116	0.956 (0.119)	0.001 (-0.025 to 0.028)	0.915
	Friendships	130	0.915 (0.147)	116	0.940 (0.143)	0.003 (-0.031 to 0.037)	0.879
	Assistance	131	0.970 (0.093)	116	0.983 (0.080)	-0.001 (-0.023 to 0.02)	0.904
	Roles	131	0.927 (0.181)	116	0.961 (0.117)	0.025 (-0.007 to 0.057)	0.119
	Confidence	131	0.936 (0.158)	116	0.970 (0.075)	0.015 (-0.012 to 0.041)	0.269
12 months	Injure	130	0.975 (0.082)	109	0.950 (0.177)	-0.018 (-0.05 to 0.014)	0.272
	Cope	130	0.943 (0.105)	109	0.957 (0.121)	-0.007 (-0.036 to 0.021)	0.614
	Friendships	130	0.918 (0.141)	109	0.944 (0.143)	-0.008 (-0.045 to 0.028)	0.650
	Assistance	129	0.969 (0.117)	110	0.979 (0.086)	-0.007 (-0.030 to 0.015)	0.528
	Roles	129	0.953 (0.121)	110	0.966 (0.083)	-0.005 (-0.039 to 0.029)	0.769
	Confidence	129	0.950 (0.122)	110	0.963 (0.076)	-0.005 (-0.033 to 0.023)	0.710
24 months	Injure	113	0.985 (0.047)	112	0.955 (0.159)	0.004 (-0.029 to 0.037)	0.804
	Cope	112	0.963 (0.099)	111	0.956 (0.159)	0 (-0.030 to 0.028)	0.963
	Friendships	130	0.918 (0.127)	111	0.939 (0.136)	-0.005 (-0.042 to 0.032)	0.805
	Assistance	112	0.991 (0.043)	113	0.977 (0.138)	-0.002 (-0.0248 to 0.021)	0.880
	Roles	113	0.966 (0.113)	113	0.953 (0.138)	-0.012 (-0.047 to 0.022)	0.478
	Confidence	113	0.975 (0.046)	112	0.954 (0.121)	-0.009 (-0.037 to 0.020)	0.546

Injure: Likely to injure self; Cope: Coping with life demands; Friendships: Ability to have friendships; Assistance: Organising assistance; Roles: Difficult to fulfil roles; Confidence: Confidence to join activities.
CI: confidence interval.

For peer review only

Supplemental Table A3: NEI-VFQ-25 Subscale and Composite Scores in participants treated with micropulse subthreshold laser vs. standard threshold laser

Trial group by timepoint	Variable	Observations	Mean	Standard deviation	Minimum	Maximum
**Subthreshold Micropulse laser**						
Baseline	*NEI-VFQ-25 Composite Score*	*131*	*86.38*	*13.83*	*30.88*	*100*
	General Health	131	46.95	25.39	0.00	100
	General Vision	130	72.15	13.75	40.00	100
	Ocular Pain	131	85.02	20.65	0.00	100
	Near Activities	130	80.00	19.84	8.33	100
	Distance Activities	130	87.82	16.09	16.67	100
	Vision Social Function	130	94.62	13.56	37.50	100
	Vision Mental Health	131	82.16	19.95	18.75	100
	Vision Role Difficulties	130	83.85	23.11	0.00	100
	Vision Dependency	131	92.68	19.28	8.33	100
	Driving	92	92.84	12.08	33.33	100
	Color Vision	129	96.71	11.85	50.00	100
	Peripheral Vision	130	88.85	18.95	25.00	100
12 months	*NEI-VFQ-25 Composite Score*	*113*	*89.61*	*9.99*	*45.92*	*100*
	General Health	113	50.00	23.62	0.00	100
	General Vision	112	74.11	13.05	40.00	100
	Ocular Pain	113	89.82	17.40	12.50	100
	Near Activities	113	83.67	19.30	8.33	100
	Distance Activities	112	90.29	15.06	33.33	100
	Vision Social Function	113	96.68	8.35	50.00	100
	Vision Mental Health	113	86.06	15.38	18.75	100
	Vision Role Difficulties	112	86.27	20.95	0.00	100
	Vision Dependency	113	96.31	12.35	25.00	100
	Driving	77	94.53	9.57	50.00	100
	Color Vision	113	98.45	8.37	25.00	100
	Peripheral Vision	112	93.08	14.32	50.00	100
24 months	*NEI-VFQ-25 Composite Score*	*114*	*87.19*	*14.08*	*22.65*	*100*
	General Health	114	52.85	29.33	0.00	100
	General Vision	114	72.63	15.57	20.00	100
	Ocular Pain	114	88.38	16.61	25.00	100
	Near Activities	114	81.18	21.55	0.00	100
	Distance Activities	114	87.35	17.57	12.50	100
	Vision Social Function	113	95.24	12.87	25.00	100
	Vision Mental Health	113	83.13	20.22	0.00	100
	Vision Role Difficulties	113	85.07	24.31	0.00	100
	Vision Dependency	111	93.09	19.72	0.00	100
	Driving	81	91.82	13.57	16.67	100
	Color Vision	110	97.73	9.90	50.00	100
	Peripheral Vision	113	88.50	20.60	25.00	100
**Standard threshold laser**						

For peer review only

Baseline	*NEI-VFQ-25 Composite Score*	*130*	*87.00*	*12.73*	*44.63*	*100*
	General Health	130	51.92	25.22	0.00	100
	General Vision	130	72.92	15.52	40.00	100
	Ocular Pain	130	85.38	17.82	25.00	100
	Near Activities	130	80.16	19.33	25.00	100
	Distance Activities	130	89.01	14.17	41.67	100
	Vision Social Function	130	93.94	13.68	25.00	100
	Vision Mental Health	130	80.87	20.62	6.25	100
	Vision Role Difficulties	129	82.75	26.48	0.00	100
	Vision Dependency	130	93.72	14.57	33.33	100
	Driving	91	95.05	9.38	50.00	100
	Color Vision	130	96.35	11.69	25.00	100
	Peripheral Vision	130	91.54	16.63	25.00	100
12 months	*NEI-VFQ-25 Composite Score*	*117*	*88.47*	*13.78*	*29.21*	*100*
	General Health	116	49.78	25.00	0.00	100
	General Vision	116	75.17	14.59	40.00	100
	Ocular Pain	117	88.25	15.25	37.50	100
	Near Activities	117	83.62	19.29	8.33	100
	Distance Activities	117	88.89	17.02	25.00	100
	Vision Social Function	116	94.29	14.22	25.00	100
	Vision Mental Health	117	86.38	18.99	6.25	100
	Vision Role Difficulties	117	85.26	24.27	0.00	100
	Vision Dependency	116	92.74	19.37	0.00	100
	Driving	81	93.26	14.65	33.33	100
	Color Vision	115	98.48	6.86	50.00	100
	Peripheral Vision	115	91.96	16.07	25.00	100
24 months	*NEI-VFQ-25 Composite Score*	*115*	*88.80*	*13.78*	*29.08*	*100*
	General Health	115	51.96	24.59	0.00	100
	General Vision	114	74.39	14.94	20.00	100
	Ocular Pain	115	89.35	15.55	37.50	100
	Near Activities	115	82.79	20.03	16.67	100
	Distance Activities	115	89.53	17.41	16.67	100
	Vision Social Function	115	94.57	13.05	12.50	100
	Vision Mental Health	115	85.43	21.26	0.00	100
	Vision Role Difficulties	114	86.73	21.76	0.00	100
	Vision Dependency	114	94.01	19.03	0.00	100
	Driving	78	96.42	7.35	58.33	100
	Color Vision	115	96.52	11.89	25.00	100
	Peripheral Vision	115	91.96	16.74	25.00	00

Reproduced with permission from Lois et al (2022).(1) This is an open access article distributed in accordance with the Creative Commons Attribution 4.0 Unported (CC BY 4.0) license, which permits others to copy, redistribute, remix, transform and build upon this work for any purpose, provided the original work is properly cited, a link to the licence is given, and indication of whether changes were made. See: https://creativecommons.org/licenses/by/4.0/. The table includes minor additions and formatting changes to the original table.

For peer review only

Supplemental Table A4: Frequency of laser treatments and use of anti-VEGFs or steroids as rescue treatment over the 24-month follow-up period

	Subthreshold Micropulse Laser	Standard Threshold Laser	Difference (95% CI)	P-value
**Number of laser treatments used from baseline to month 24 in study eye [a b]**	2.37 (0.11) n=133	1.89 (0.11) n=132	0.48 (0.18, 0.79)	0.002
**Number of patients with at least one steroid injection in study eye (as additional treatment) from baseline to month 24[c]**	0 (0.0%)	1 (0.8%)		
**Number of patients receiving at least one anti-VEGF treatment (as additional treatment) from baseline to month 24[c]**	24 (18.1%) n=133	28 (21.2%) n=132	OR: 0.78 (0.42 – 1.45)	0.44
			% point difference: -2.8 (-13.1 – 7.5)	0.59
**Number of anti-VEGF treatments (as additional treatment) from baseline to month 24[a d]**	0.80 (0.23) n=133	1.30 (0.23) n=132	-0.50 (-1.14 – 0.14)	0.13
**Number of anti-VEGF treatments (as additional treatment) from baseline to month 24 [d e]**				
**1-2**	4 (16.7%) n=24	7 (25.0%) n=28		
**3-4**	10 (41.7%) n=24	7 (25.0%) n=28		
**5-10**	10 (41.7%) n=24	9 (32.1%) n=28		
**>10**	0 (0.0%) n=24	5 (17.9%) n=28		

[a] Mean (SE) presented for continuous outcomes [b] Number of laser treatments, were analysed using linear regression with adjustment for baseline BCVA and minimisation variables. [c] number of patients receiving at least one additional treatment (defined as at least one anti-VEGF or steroid), were analysed using logistic regression models with adjustment for the minimisation variables. [d] Number of steroid injections and number of anti-VEGF treatments (as mean and number (%) in categories), were analysed using linear regression with adjustment for minimisation variables. [e]n (%) based on no. of patients receiving anti VEGF treatments.

anti-VEGF: anti-vascular endothelial growth factor; CI: confidence interval; OR: odds ratio; RR: risk ratio

References

1. Lois N, Campbell C, Waugh N, Azuara-Blanco A, Maredza M, Mistry H, et al. DIAbetic Macular Oedema aNd Diode Subthreshold micropulse laser (DIAMONDS): A pragmatic, multicentre, allocation concealed, double-masked prospective, randomised, non-inferiority, clinical trial. Health Technology Assessment. 2022;26(50):1-86.

CHEERS Checklist

Topic	No.	Item	Location where item is reported
**Title**			
	1	Identify the study as an economic evaluation and specify the interventions being compared.	1
**Abstract**			
	2	Provide a structured summary that highlights context, key methods, results, and alternative analyses.	4
**Introduction**			
Background and objectives	3	Give the context for the study, the study question, and its practical relevance for decision making in policy or practice.	6
**Methods**			
Health economic analysis plan	4	Indicate whether a health economic analysis plan was developed and where available.	6
Study population	5	Describe characteristics of the study population (such as age range, demographics, socioeconomic, or clinical characteristics).	6
Setting and location	6	Provide relevant contextual information that may influence findings.	6
Comparators	7	Describe the interventions or strategies being compared and why chosen.	6
Perspective	8	State the perspective(s) adopted by the study and why chosen.	6
Time horizon	9	State the time horizon for the study and why appropriate.	6
Discount rate	10	Report the discount rate(s) and reason chosen.	7
Selection of outcomes	11	Describe what outcomes were used as the measure(s) of benefit(s) and harm(s).	8-9
Measurement of outcomes	12	Describe how outcomes used to capture benefit(s) and harm(s) were measured.	8-9
Valuation of outcomes	13	Describe the population and methods used to measure and value outcomes.	8-9

For peer review only - http://bmjopen.bmj.com/site/about/guidelines.xhtml

Topic	No.	Item	Location where item is reported
Measurement and valuation of resources and costs	14	Describe how costs were valued.	8
Currency, price date, and conversion	15	Report the dates of the estimated resource quantities and unit costs, plus the currency and year of conversion.	7
Rationale and description of model	16	If modelling is used, describe in detail and why used. Report if the model is publicly available and where it can be accessed.	N/A
Analytics and assumptions	17	Describe any methods for analysing or statistically transforming data, any extrapolation methods, and approaches for validating any model used.	9
Characterising heterogeneity	18	Describe any methods used for estimating how the results of the study vary for subgroups.	N/A
Characterising distributional effects	19	Describe how impacts are distributed across different individuals or adjustments made to reflect priority populations.	N/A
Characterising uncertainty	20	Describe methods to characterise any sources of uncertainty in the analysis.	10
Approach to engagement with patients and others affected by the study	21	Describe any approaches to engage patients or service recipients, the general public, communities, or stakeholders (such as clinicians or payers) in the design of the study.	N/A
Results			
Study parameters	22	Report all analytic inputs (such as values, ranges, references) including uncertainty or distributional assumptions.	1 (Suppl A1)
Summary of main results	23	Report the mean values for the main categories of costs and outcomes of interest and summarise them in the most appropriate overall measure.	10-13
Effect of uncertainty	24	Describe how uncertainty about analytic judgments, inputs, or projections affect findings. Report the effect of choice of discount rate and time horizon, if applicable.	13
Effect of engagement with patients and others affected by the study	25	Report on any difference patient/service recipient, general public, community, or stakeholder involvement made to the approach or findings of the study	N/A
Discussion			

Topic	No.	Item	Location where item is reported
**Study findings, limitations, generalisability, and current knowledge**	26	Report key findings, limitations, ethical or equity considerations not captured, and how these could affect patients, policy, or practice.	13-14
**Other relevant information**			
**Source of funding**	27	Describe how the study was funded and any role of the funder in the identification, design, conduct, and reporting of the analysis	2
**Conflicts of interest**	28	Report authors conflicts of interest according to journal or International Committee of Medical Journal Editors requirements.	2

From: Husereau D, Drummond M, Augustovski F, et al. Consolidated Health Economic Evaluation Reporting Standards 2022 (CHEERS 2022) Explanation and Elaboration: A Report of the ISPOR CHEERS II Good Practices Task Force. Value Health 2022;25. doi:10.1016/j.jval.2021.10.008

For peer review only

For peer review only - http://bmjopen.bmj.com/site/about/guidelines.xhtml
